# Viscous remanent magnetization authentication method for archaeological artifacts

**DOI:** 10.1073/pnas.2620397123

**Published:** 2026-09-03

**Authors:** Yoav Vaknin, Brendan Cych, Jeffrey S. Gee, Lisa Tauxe

**Affiliations:** ^a^ Geosciences Research Division, Scripps Institution of Oceanography, University of California San Diego, La Jolla, CA 92093-0220; ^b^ Department of Earth, Ocean and Ecological Sciences, University of Liverpool, Liverpool L69 3GP, United Kingdom

**Keywords:** viscous remanent magnetization, archaeomagnetism, authentication, numerical modeling, rock magnetism

## Abstract

Museums and public collections often contain archaeological clay artifacts of unknown origin. Such artifacts, if proven to be authentic, may contribute significantly to our understanding of ancient societies. However, the authenticity of these artifacts is usually highly debated, resulting in their being disregarded in historical interpretations. Several laboratory methods have been developed in order to authenticate such objects but none have been proven infallible. Here we present a method for distinguishing between authentic and fake clay artifacts by considering the Viscous Remanent Magnetization (VRM) acquired by the artifacts. Clay objects tend to acquire a strong magnetic signal termed Thermal Remanent Magnetization (TRM) upon heating and cooling in kilns during their manufacturing process. After being removed from the orientation in which their TRM was acquired, these objects were exposed to an ambient geomagnetic field in a different direction. Over archaeological time scales, this results in the acquisition of a significant VRM. We propose a laboratory protocol for identifying this ancient VRM. We validated the protocol using micromagnetic modeling of single domain particles and by laboratory experiments carried out on 19 ancient and 26 modern objects, made from different types of clay and fired using different firing techniques. Both the experiments and the simulations indicate that the existence of a significant VRM component removed at temperatures higher than a cutoff temperature of 112 ^°^C is indicative of ancient objects while its removal at lower temperatures is indicative of modern objects. We successfully applied this method to objects of debated authenticity.

## Significance

This study introduces a magnetic method which can distinguish between fake and authentic archaeological clay objects. The findings of the method are useful for museum curators who wish to display only authentic artifacts and for archaeologists and historians, studying the societies which created them. It can also help law enforcement authorities in their effort to eliminate illicit antiquities trade, which involves looting archaeological sites and forgery. From a geophysical aspect, the combination of computer-based models and laboratory experiments can significantly improve Viscous Remanent Magnetization dating, a method which has been used over the last seven decades. This method can become more widely used, thanks to these improvements, and applied to resolve many archaeological and geological issues.

Museums, universities, and national archaeological institutions often have substantial collections of clay archaeological artifacts whose provenance and authenticity are uncertain. Due to their debated authenticity, such artifacts are frequently excluded from exhibition and from scholarly interpretation. For fired clay, the most widely used authentication method is Thermoluminescence (1, 2). Despite its effectiveness, there are means by which forgers can create a fake object that will seem authentic using this method (3, 4) and its use sometimes leads to debate among scholars (5–7). Another method which is sometimes used for authentication is petrography (8, 9), which identifies the types of clay that the object consists of. However, since forgers can foil this method by simply using the local clays that were used in antiquity, it sometimes leads to debated authenticity as well (10). To fight illicit antiquities trade, law enforcement authorities are in need of reliable authentication methods. For example, in a famous forgery trial, a collector and an antiquity dealer were accused of forging the ossuary of James, brother of Jesus, and several biblical related objects. After a seven year trial, in 2012 the primary defendants were acquitted of the major forgery charges. Although experts concluded that some of the objects, including clay objects (8), were likely forged, the court ruled that the forgery had not been proven beyond reasonable doubt (11).

In this paper, we address the authentication issue through a geophysical (paleomagnetic) methodology, treating fired clay as a magnetic recorder. We evaluate whether its remanence components, their stability, and time–temperature characteristics align with prolonged exposure to the Earth’s magnetic field consistent with the presumed dating. During manufacture of clay artifacts, the process of heating and cooling in a kiln imparts a thermal remanent magnetization (TRM), a strong and relatively stable magnetic signal that records the geomagnetic field at the time of firing. After deposition and burial, prolonged exposure to the ambient geomagnetic field in a different orientation from that in the kiln leads to the acquisition of a viscous remanent magnetization (VRM). In most paleomagnetic experiments this component is treated as noise which should be erased in order to identify the magnetic signal of interest in rocks or archaeological artifacts. Here we present a tool based on stepwise thermal demagnetization to isolate the TRM and VRM components carried by fired-clay artifacts and then estimate the time over which the VRM was acquired using physics-based relaxation modeling for single-domain particles (12, 13).

At the end of the nineteenth century, Folgerhaiter led one of the first efforts in VRM research. His study was based on archaeomagnetism, assessing the change in magnetization in Roman bricks according to the orientation in which they were placed after firing (14). The basic theory of single domain VRM acquisition was proposed half a century later by Néel (15, 16). Despite significant VRM studies that followed (17–24), Néel theory is still widely used regarding single domain VRM.

Over the last seven decades, VRM dating has been applied to rocks in order to estimate the time of displacement (25–31). However, it has only been applied several times in archaeology and only to rocks used as building materials (32–36). To our knowledge, until the current research, VRM dating has never been applied to fired clay. In this study, we apply micromagnetic modeling, using the Single Domain Comprehensive Calculator (SDCC) (12, 13). The blocking-temperature/relaxation-time relationships produced from SDCC have demonstrated clear improvements over the so-called “Pullaiah curves” (37) derived from classical Néel theory (15, 16). As predicted by the SDCC, our experiments show that detection of an “old” VRM is consistent with an ancient firing and long residence time, whereas the absence of an old VRM is consistent with recent manufacture, reheating, or modern remagnetization.

## Materials and Methods

We applied both micromagnetic modeling and laboratory experiments in order to validate the VRM authentication method.

### Micromagnetic Modeling.

For micromagnetic modeling we used the SDCC software package (12, 13) to simulate the VRM acquisition under “ancient” and “modern” conditions. Both types of simulated samples consisted of the same range of magnetic particle sizes and shapes (*SI Appendix*, Fig. S1) and acquired their simulated TRM under the same conditions (*SI Appendix*, Fig. S3): “heating” to 597 ^°^C and “cooling” in an ambient field of 44 μT (the modern field in the Levant). Then they both were “placed” in an ambient field of 44 μT, perpendicular to the direction of their TRM. The object was “placed” in this field for 0.5, 10, 100, 1,000, and 2,500 y. We consider the 1,000 and 2,500 results to be applicable for “ancient” objects whereas the modern objects would be the simulations for 100 or fewer years. Then, we simulated thermal demagnetization of the samples in a step-wise fashion, in 10 ^°^C steps from 50 ^°^C to 200 ^°^C. For further details see: *SI Appendix*, *Extended Methods* and Fig. S23.

### First-Order Reversal Curve (FORC).

Micromagnetic modeling requires assumptions about the size and shape distributions of the ferromagnetic particles in the samples. To ensure that the simulation results are relevant to our samples, we used a Lakeshore 8604 vibrating sample magnetometer (VSM) in order to measure first-order reversal curves (FORC) (38). We used FORCme v1.0.1 software package (39) for the FORC processing. See *SI Appendix*, *Extended Methods* for details. Based on the results of our simulations, we carried out experiments on a collection of samples that were known to be ancient or known to be modern as well as several whose ages were unknown.

### Thermal Demagnetization of Ancient and Modern Artifacts.

**Fig. 1.**
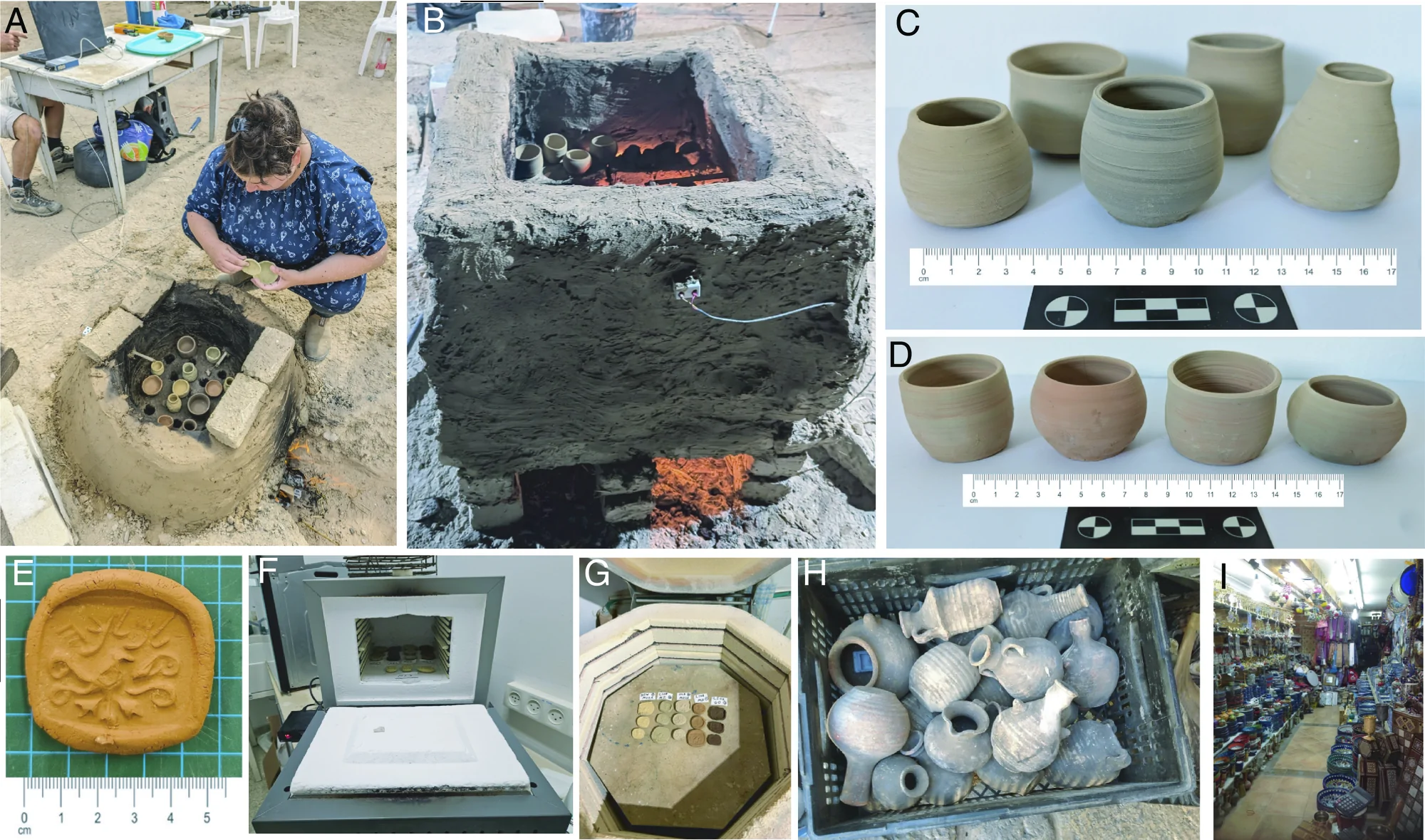
Modern pottery used for this research. (*A*–*D*) Pottery vessels created from five different types of clay by Bar-Sovik et al. (42) and used for this research as well. Photos courtesy of Lior Bar-Sovik. (*A*) Lior Bar-Sovik placing vessels in one of the two traditional kilns. (*B*) Firing vessels in the second traditional kiln. (*C*–*D*) Nine representative vessels. (*E*) A “fake” clay seal impression prepared as part of this research. The seal was made by Rachel Pelta. (*F* and *G*) Two of the four electric ovens used for firing the seal impressions. (*H*) Pottery vessels sold as souvenirs in the old city of Jerusalem. (*I*) One of the souvenir shops in which we purchased souvenir pottery vessels.

We used 45 samples which had been proven to be ancient or modern: 15 ancient Royal Judean Storage Jars (40) and four ancient pottery sherds (41) which had been unearthed in excavations, six modern pottery vessels fired in two different traditional kilns (Fig. 1*A*–*D*) (42), 16 clay seal impressions fired in modern ceramic kilns prepared for this study (Fig. 1*E*–*G*) and four modern pottery vessels sold as souvenirs for tourists in Jerusalem (Fig. 1 *H* and *I*). We also applied the VRM removal method to three artifacts of questionable authenticity which had been confiscated by the Israel Antiquities Authority due to illegal antiquities trade.

One specimen from each sample was glued in a square alumina crucible, 2.2×2.2×2 cm in size, which had a magnetic moment weaker than $5\cdot 10^{-11}$ Am^2^. The specimens were wrapped in silica fiber wool and glued using potassium silica glue (KASIL). The experiments were conducted in the Paleomagnetic Laboratory at Scripps Institution of Oceanography, University of California, San Diego, using a 2-G cryogenic magnetometer and a thermal demagnetizer. After measuring the initial magnetic moments of the specimens, we demagnetized them thermally using the same steps as in the micromagnetic modeling from 50 ^°^C to 200 ^°^C (*SI Appendix*, *Extended Methods* and Figs. S3 and S18). In some cases the process continued to higher steps (225 ^°^C, 250 ^°^C, 275 ^°^C, 300 ^°^C, 350 ^°^C, 400 ^°^C, 440 ^°^C, 480 ^°^C, 520 ^°^C, 560 ^°^C, and 600 ^°^C) until at least 20% of the initial magnetic moment had been removed. At every step, the specimens were kept at the desired temperature for 30 min before cooling.

We calculated the VRM removal temperature (VRT) using a Python code called VRM Removal Temperature Calculator (VRTC). The VRTC uses the demagnetization data starting from the initial moment and ending at the first step in which the remaining moment is weaker than 80% of the initial moment. For every demagnetization step, the Deviation Angle (DANG) (43, 44) is calculated using a group of measurements consisting of that step and the four following steps and is the angle between the component direction and the origin. Both the moment and the DANG functions are smoothed using a Savitzky–Golay (45) filter. The VRTC then calculates the VRM removal temperature by two methods: i) The temperature at which the magnetic moment curves sharply and decays persistently thereafter to the origin, marked VRT_M_. ii) The temperature at which the DANG drops significantly and stays under a defined threshold thereafter, marked VRT_D_ (*SI Appendix*, *Extended Methods*). Finally, we take the average of the VRM temperatures calculated by these two methods as the VRT.

## Results

**Fig. 2.**
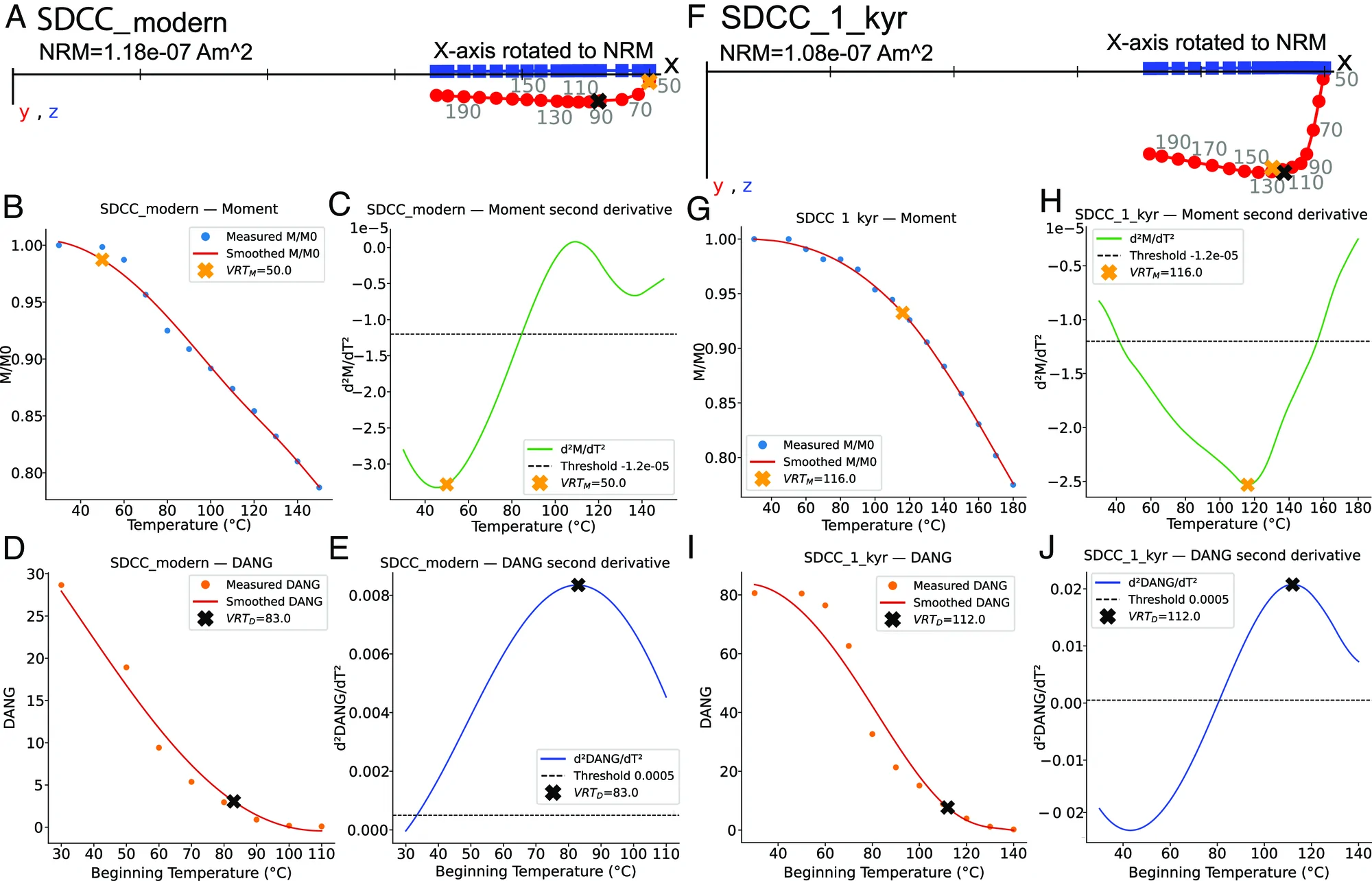
Simulation results. (*A*–*E*) “Modern” object. (*A*) Zijderveld (46) diagram presenting thermal demagnetization. (*B*) The magnetic moment, normalized to the initial moment, remaining after every thermal demagnetization step (blue dots) and a curve representing the data after smoothing by a Savitzky–Golay (45) filter (red line). (*C*) The second derivative of the curve presented in (*B*). The VRM removal temperature according to the moment (VRT_M_) is marked by the orange X here and in (*A* and *B*). (*D*) The DANG calculated from every starting point (orange dots- see text for details) and a curve representing the data after smoothing by a Savitzky–Golay (45) filter (red line). (*E*) The second derivative of the curve presented in (*D*). The VRM removal temperature according to the DANG (VRT_D_) is marked by the black X here and in (*A* and *D*). (*F*–*J*) Same as A-E but for an “ancient” object. The VRM component of this simulated object is removed at a higher temperature than that of the modern object in (*A*–*E*).

**Fig. 3.**
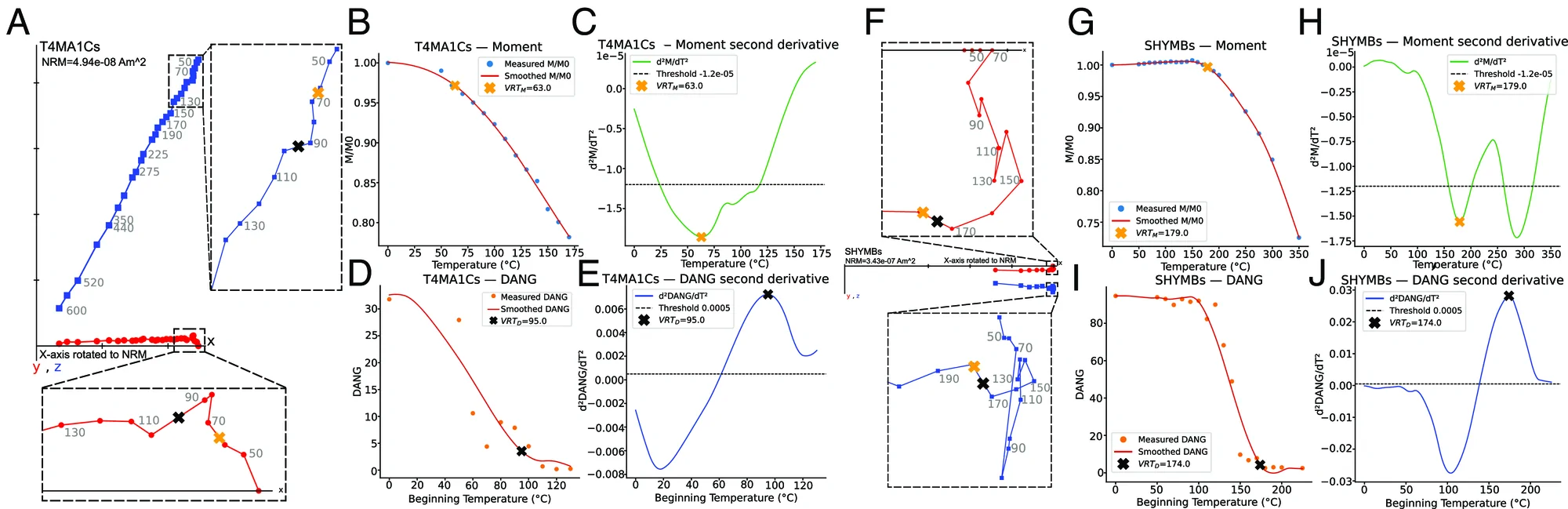
Representative results of laboratory thermal demagnetization. Subfigures same as in Fig. 2, with the addition of expanded views of each of the two components in (*A* and *F*). (*A*–*E*) A modern specimen. (*F*–*J*) An ancient specimen.

**Fig. 4.**
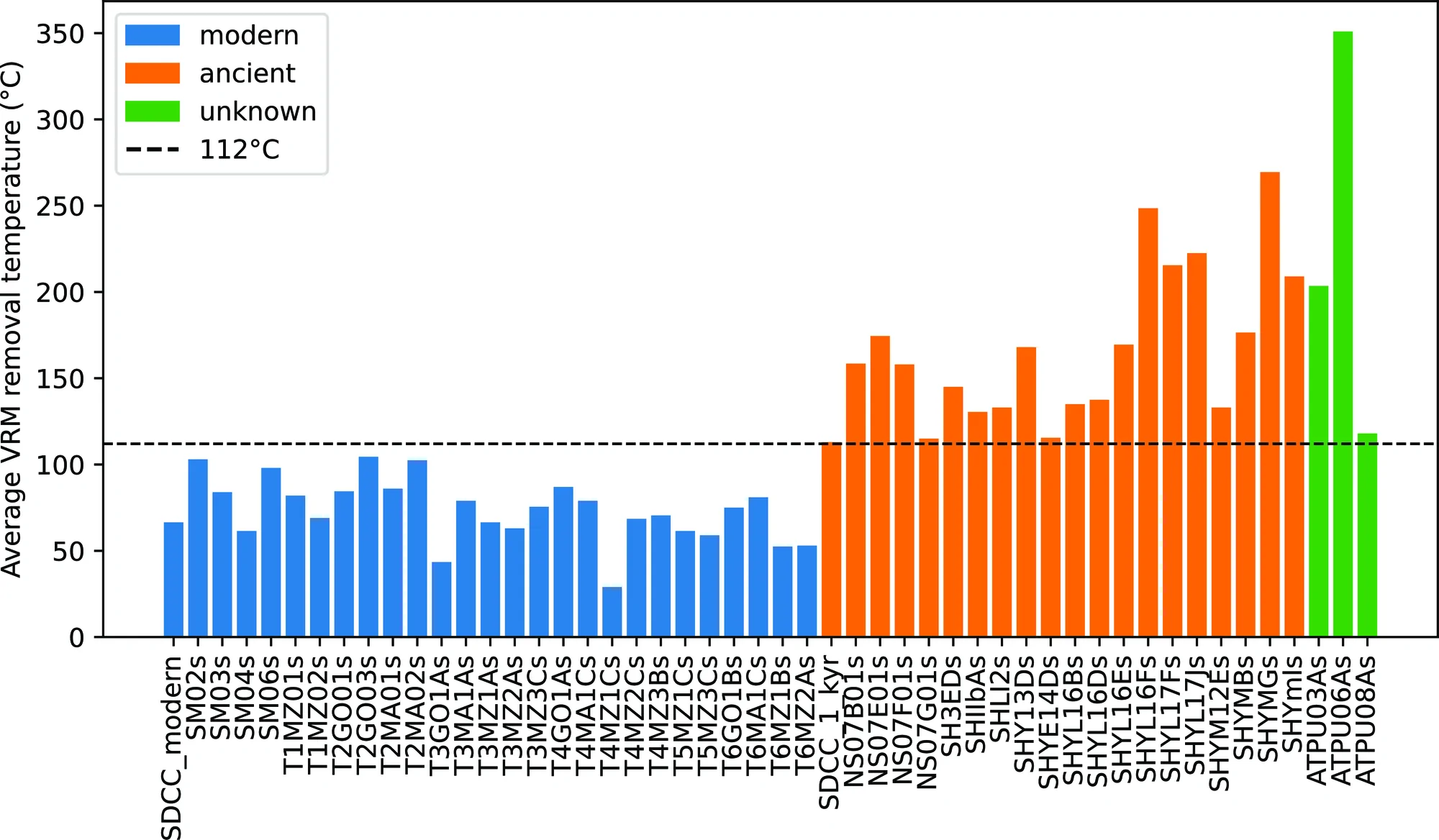
VRM Removal Temperature (VRT) results of all 48 actual specimens and two simulated specimens. The results are based on the VRTC python code (the average of the VRT_D_ and the VRT_M_). The dashed black line marks the 112 ^°^C threshold, which is the VRT_D_ of the 1,000-year-old simulated specimen.

The micromagnetic model simulations (Fig. 2) and the laboratory measurements on the artifacts (Fig. 3) showed very similar results (Fig. 4): Modern artifacts have a small VRM component, if any, and it is removed well below 112 ^°^C. Ancient artifacts have a much more significant VRM component and it is completely removed only above 112 ^°^C. The difference in VRM removal temperature (VRT), with a 112 ^°^C threshold, can be used as a method for authenticating archaeological clay artifacts which are supposed to be, according to their typology, older than 1,000 y.

### Micromagnetic Modeling.

We plot the simulated results of the thermal demagnetization experiments as Zijderveld (46) diagrams in Fig. 2 *A* and *F*. The VRTC results are presented in Fig. 2*B*–*E* (“modern”) and *G*–*J* (“ancient”). In the latter case, the VRTC yields a VRT_M_ of 116 ^°^C and VRT_D_ of 112 ^°^C. Therefore, we consider 112 ^°^C as the threshold for objects older than 1,000 y. Since the simulated data lack experimental noise, it is clear from the Zijderveld diagram (Fig. 2*F*) that a minor VRM component exists until ∼130 ^°^C, but it is too small for the VRTC to identify it. Because there is an exponential relationship between the removal temperature and time, there is little difference between the results for 1,000 y of aging as opposed to, say, 2,500 y (*SI Appendix*, *Extended Results* and Fig. S23). In the “modern” simulation the VRT_M_ is 50 ^°^C and the VRT_D_ is 83 ^°^C (Fig. 2*A*–*E*). The effect of particle shape on VRM removal temperature is presented in *SI Appendix*, Fig. S24 and *Extended Results*.

### Thermal Demagnetization.

All 26 modern specimens (Fig. 3*A*–*E* and *SI Appendix*, Table S1) yielded VRTs below the 112 ^°^C threshold and all 19 ancient specimens (Fig. 3*F*–*J* and *SI Appendix*, Table S2) yielded VRTs above this threshold, as expected (Fig. 4). In three of these 45 specimens, one modern and two ancient, there is a contradiction between the VRT_D_ and the VRT_M_ (*SI Appendix*, Figs. S3 and S4). One of the three is a modern specimen (T2GO03s) that yielded a VRT_D_ which is higher than the threshold but its VRT_M_ and the average are not. The ancient specimen SHYE14Ds yielded a VRT_D_ of 136 ^°^C and a VRT_M_ of 95 ^°^C. Although the average (VRT = 115.5 ^°^C) is above the 112 ^°^C threshold, one should take special notice in such cases. The difference between the VRM and TRM can be seen at 110 to 120 ^°^C in one projection of the vector (red zoom-in in *SI Appendix*, Fig. S21*A*) but not in the other (blue zoom-in in *SI Appendix*, Fig. S21*A*). We suggest that in this case the VRM was recorded, probably by chance (*SI Appendix*, *Extended Results*), in an orientation similar to that of the TRM. Therefore, the TRM and VRM components are in very similar directions and our method is limited in its ability to distinguish between them. Four other ancient specimens (NS07B01s, NS07E01s, NS07F01s, and NS07G01s) were chosen for this research in order to examine this possibility. The original magnetic signals of these pottery sherds, recorded during their manufacture, were completely overprinted by a signal recorded during a conflagration event. After this secondary heating, these sherds remained for thousands of years in an orientation close to that in which they had been heated (41). However, three of the four yielded conclusive VRTs (*SI Appendix*, Table S2), demonstrating that even a slight change in orientation can yield a significant VRM, identifiable by the VRTC. Only one of the four (NS07E01s) yielded contradicting VRT_M_ and VRT_D_ results due to the similar directions of the TRM and VRM.

### First-Order Reversal Curve (FORC).

According to the FORC (38) measurements, the vast majority of the specimens contain a dominant single domain component (*SI Appendix*, Figs. S8–S11 and *Extended Results*). This supports the validity of thermal demagnetization as a tool for authentication for clay objects, as demonstrated by the micromagnetic modeling. The hysteresis measurements are shown in *SI Appendix*, Figs. S4–S7 and the FORC diagrams are in *SI Appendix*, Figs. S8–S11. Here we also include the “FORC density profile plots” (*SI Appendix*, Figs. S12–S16) which are FORC densities against the two FORC axes, Bc and Bu (*SI Appendix*, *Extended Results*).

The hysteresis measurements help constrain the expected unblocking temperatures for the finest grains in our samples. Of particular importance are the FORC density plots (*SI Appendix*, Figs. S12–S16). These show strong peaks in FORC densities between 2 and 16 mT for all samples. The micromagnetic modeling of particles with coercivities between 2 and 40 mT should have unblocking temperatures ranging from 60 to 95 ^°^C (*SI Appendix*, Fig. S17) strongly supporting our use of VRM removal temperatures in verifying the authenticity of archaeological clay artifacts.

### Applications.

**Fig. 5.**
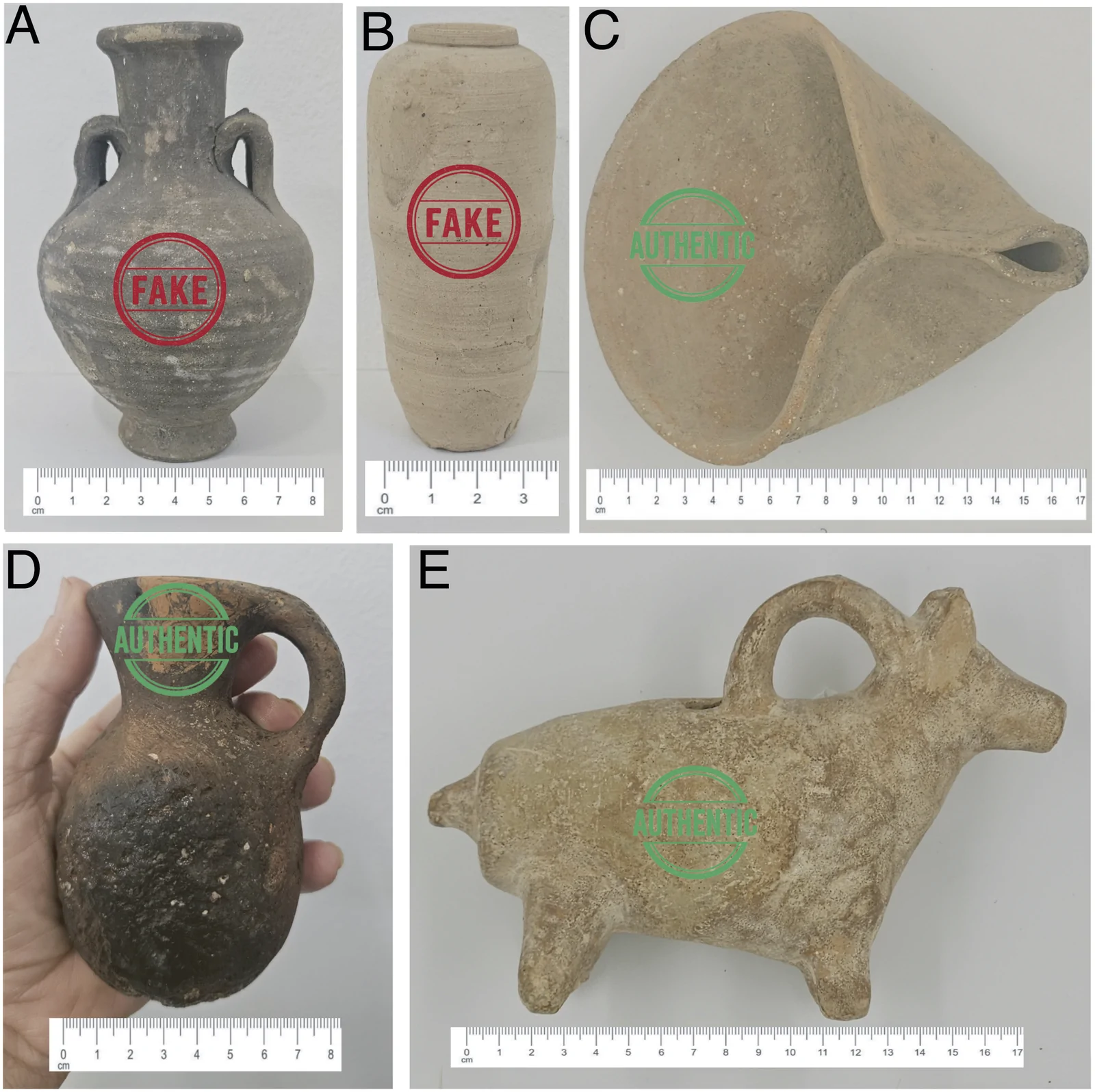
Representative studied artifacts. (*A* and *B*) Artifacts purchased from the souvenir market, known to be modern. (*A*) SM02. (*B*) SM06. (*C*–*E*) Artifacts received from the Antiquities Theft Prevention Unit (ATPU) of the Israel Antiquities Authority (IAA). All three objects appear to be authentic. (*C*) ATPU08. (*D*) ATPU06. Notice the black color on the *Bottom* of the juglet (*D*), suggesting it was fired again after its production. (*E*) ATPU03.

We applied our method to three artifacts received from the Antiquities Theft Prevention Unit (ATPU) of the Israel Antiquities Authority (Fig. 5*C*–*E*). Our results are consistent with the idea that all three artifacts are authentic (Fig. 4 and *SI Appendix*, Table S3). The VRT of ATPU06 (351 ^°^C) is clearly too high to represent the temperature at which the VRM was removed. It seems that this artifact was heated after its initial production to 350 ^°^C, thus partially erasing the initial magnetization recorded in the kiln (*SI Appendix*, Fig. S22). This secondary firing could have occurred in antiquity, perhaps during a conflagration, which is common in the archaeological record (47). This possibility is supported by the black color on part of this juglet (Fig. 5*D*), which is unlikely to be the result of the firing in the kiln. The partial TRM seems to be overprinted by a VRM which is removed at 200 to 250 ^°^C.

## Discussion and Conclusions

It has been many years since VRM dating was first suggested (25) and yet, it is not widely used. In some cases, VRM dating of geological materials tends to yield older estimated ages than other dating methods (27, 31, 48). In this paper we showed that VRM dating can be a reliable tool for the authentication of clay archaeological artifacts. There are several possible reasons for the success of VRM dating for this application. First, we relied on micromagnetic modeling which has been shown to be a significant improvement on the traditional Néel theory approach for estimating relaxation time–temperature curves (12, 13) (*SI Appendix*, Fig. S24). Second, although VRM was discovered through the research of fired clay bricks (14), this is the first use of VRM dating on fired clay. It is well known that fired clay, often dominated by low coercivity particles (*SI Appendix*, Figs. S12–S16), is a good recorder of the ambient field at the time of firing (42). In this study we found that in the case of fired clay the typical strong and unified signal (TRM) is especially easy to distinguish from the VRM that is recorded after the firing. Furthermore, typical rocks used for VRM dating acquired their initial magnetization on geological time scales, much earlier than typical pottery, thus increasing the probability of exposure to different types of magnetic overprints. Also, typical geological materials are rarely single domain so estimating the expected VRT is challenging. Finally, the VRM of shifted rocks was acquired over very long time periods as well and the time- resolution of VRM dating decreases logarithmically with increased time of VRM acquisition (27) (*SI Appendix*, Fig. S23). All these reasons make it more straightforward to apply VRM dating to fired pottery than to rocks. Furthermore, the method presented here is a relatively “easy” application of VRM dating, due to the large difference in VRM acquisition, percentage-wise, between an ancient artifact and a modern forgery. According to our results, when dealing with an artifact of unknown authenticity, if its VRT is >112 ^°^C the artifact is most likely more than 1,000 years old and if the VRT is significantly smaller than 112 ^°^C, the artifact is most likely modern. For authentic artifacts younger than 1,000 y and/or for forgeries made decades or even several centuries ago, our method is limited and perhaps requires a different threshold (*SI Appendix*, *Extended Results* and Fig. S23). In cases where VRT_D_ and VRT_M_ yield contradicting results or if both yield border-line results (VRT ∼ 112 ^°^C) our method cannot deliver a decisive conclusion. Such a rare case may occur when an ancient clay artifact was placed in an orientation very similar to that in which it had been fired and remained in this orientation over archaeological time periods. This might occur by chance or if it was refired during a destruction and remained in the same orientation during postdeposition. In such cases, other authentication methods can help reach a more conclusive result. In general, the suggested method is not intended to replace other authentication methods. On the contrary, it can and should be combined with other methods. For instance, if an authentic ancient object is crushed into dust and mixed with modern binding agents, it can seem authentic according to Thermoluminescence, but our method will identify it as a forgery, given that it will not have a significant TRM. Other methods, such as petrography, can identify these modern binding agents. Furthermore, a modern forgery that has been exposed to artificial irradiation can seem authentic using Thermoluminescence (3, 4), but identified as a forgery by the method suggested here. The addition of methods, like the one presented here, to the authentication toolbox has the potential of not only authenticating artifacts of debated authenticity, but also making the future task of forgery more complicated and, hopefully, inexpedient enough to discourage potential forgers. The addition of methods can also contribute to the ongoing effort of law enforcement agencies around the world to prevent illicit antiquities trade, which involves forgery and archaeological looting.

## Supplementary Material

Appendix 01 (PDF)

## Data Availability

All data from this paper are available here: https://earthref.org/MagIC/20694 (49). The python code used for analyzing the data in the MagIC dataset is available here: https://github.com/ltauxe/VRM-removal-temperature-notebook/ and PmagPy (50), available at: https://github.com/PmagPy/PmagPy. Hysteresis curves were analyzed with version 1.0.1 of the FORCme software of (39) available at: https://github.com/Institute-for-Rock-Magnetism/FORCme.

## References

[1] G. C. Kennedy, Dating by thermoluminescence. Archaeology **13**, 147–148 (1960).

[2] M. J. Aitken, *Thermoluminescence Dating/M. J. Aitken* (Studies in archaeological science, Academic Press, London, 1985).

[3] D. Stoneham, M. Stoneham, Beating the Forger: Authenticating Ceramic Antiquities. Contemp. Phys. **51**, 397–411 (2010).

[4] J. Li , A preliminary investigation on the identification of artificial irradiation in thermoluminescence pre-dose dating of ancient Chinese porcelain. Crystals **15**, 503 (2025).

[5] H. McKerrell, V. Mejdahl, H. Henri François, G. Portal, Thermoluminescence and Glozel. Antiquity **48**, 265–274 (1974).

[6] M. J. Aitken, J. Huxtable, Thermoluminescence and Glozel. A plea for caution. Antiquity **49**, 223–226 (1975).

[7] H. V. McKerrell, V. Mejdahl, H. François, G. Portal, Thermoluminescence and Glozel: A plea for patience. Antiquity **49**, 267–272 (1975).

[8] Y. Goren, E. Arie, The authenticity of the bullae of Berekhyahu Son of Neriyahu the Scribe. Bull. Am. Sch. Orient. Res. **372**, 147–158 (2014).

[9] S. Ahituv , A seal impression of ‘Shema Servant of Jeroboam’. Tel Aviv **50**, 216–230 (2023).

[10] R. Deutsch, A. Lemaire, G. Barkay, P. Gert van der Veen, D. Itzhak, “The bulla of “Shema, servant of Jeroboam” an embarassing forgery” in *Gabriel, Tell This Man the Meaning of His Vision (Daniel, 8:16): Studies in Archaeology, Epigraphy, Iconography and the Biblical World in Honor of Gabriel Barkay on the Occasion of his 80th Birthday (22 June 2024)*, R. Deutsch, A. Lemaire, Eds. (Archaeological Center Publications, 2024), pp. 136–149.

[11] State of Israel v. Oded Golan and Robert Deutsch, Criminal Case 482/04, Delivered by Judge Aharon Farkash in the Jerusalem District Court on March 14, 2012.

[12] B. Cych, G. Paterson, L. Nagy, W. Williams, First principles understanding of single domain magnetizations - Part I: The single domain comprehensive calculator (SDCC) open source library. Geophys. J. Int. **241**, 2034–2048 (2025).

[13] B. Cych, G. A. Paterson, L. Nagy, W. Williams, L. Tauxe, First principles understanding of single domain magnetizations - Part II: Non-ideal magnetic behavior in ideal single domain titanomagnetite. Geophys. J. Int. **245**, ggag090 (2026).

[14] G. Folgerhaiter, Sur le variations seculaires de l’inclinaison magnetique dans antiquite. J. Phys. **8**, 5–16 (1899).

[15] L. Néel, Théorie du traînage magnétique des ferromagnétiques en grains fins avec applications aux terres cuites. Ann. Geophys. **5**, 99–136 (1949).

[16] L. Néel, Influence des fluctuations thermiques sur l’aimantation de grains ferromagnétiques très fins. Compt. R. Hebd. Seances Acad. Sci. **228**, 664–666 (1949).

[17] D. Walton, Time-temperature relations in the magnetization of assemblies of single domain grains. Nature **286**, 245–247 (1980).

[18] D. Walton, Viscous magnetization. Nature **305**, 616–619 (1983).

[19] D. J. Dunlop, Viscous magnetization of 0.04-100 *mu*m magnetites. Geophys. J. R. Astron. Soc. **74**, 667–687 (1983).

[20] D. Walton, D. J. Dunlop, The magnetization of a random assembly of interacting moments. Solid State Commun. **53**, 359–362 (1985).

[21] S. L. Halgedahl, Experiments to investigate the origin of anomalously elevated unblocking temperatures. J. Geophys. Res. Solid Earth **98**, 22443–22460 (1993).

[22] A. R. Muxworthy, W. Williams, Observations of viscous magnetization in multidomain magnetite. J. Geophys. Res. Solid Earth **111**, e2005JB003902 (2006).

[23] Y. Yu, L. Tauxe, Acquisition of viscous remanent magnetization. Phys. Earth Planet. Inter. **159**, 32–42 (2006).

[24] R. J. Enkin, D. J. Dunlop, The demagnetization temperature necessary to remove viscous remanent magnetization. Geophys. Res. Lett. **15**, 514–517 (1988).

[25] K. M. Creer, The remanent magnetization of unstable Keuper Marls. Philos. Trans. R. Soc. Lond. Ser. A Math. Phys. Sci. **250**, 130–143 (1957).

[26] F. Rimbert, “Contribution à l’étude de l’action de champs alternatifs sur les aimantationsrémanentes des roches. Applications géophysiques,” PhD thesis, Université de Paris (1959).

[27] J. G. Crider, D. M. Globokar, R. F. Burmester, B. A. Housen, Unblocking temperatures of viscous remanent magnetism in displaced granitic boulders, Icicle Creek glacial moraines (Washington, USA). Geophys. Res. Lett. **42**, 10,647–10,654 (2015).

[28] T. A. Berndt, A. R. Muxworthy, Dating Icelandic glacial floods using a new viscous remanent magnetization protocol. Geology **45**, 339–342 (2017).

[29] T. Sato , Two-step movement of tsunami boulders unveiled by modified viscous remanent magnetization and radiocarbon dating. Sci. Rep. **12**, 13011 (2022). 35906266 10.1038/s41598-022-17048-8 PMC9338091

[30] T. Sato , Ancient tsunami records in the viscous remanent magnetization of reworked boulders in the kingdom of Tonga. Geophys. Res. Lett. **51**, e2024GL110932 (2024).

[31] A. R. Muxworthy, J. R. Williams, D. Heslop, Testing the use of viscous remanent magnetisation to date flood events. Front. Earth Sci. **3**, 1 (2015).

[32] F. Heller, H. Markert, The age of viscous remanent magnetization of Hadrian’s Wall (Northern England)*. Geophys. J. Int. **31**, 395–406 (1973).

[33] G. J. Borradaile, An 1800-year archeological experiment in remagnetization. *Geophys. Res. Lett.* **23**, 1585–1588 (1996).

[34] G. J. Borradaile, M. Brann, Remagnetization dating of Roman and Mediaeval masonry. J. Archaeol. Sci. **24**, 813–824 (1997).

[35] G. J. Borradaile, Viscous remanent magnetization of high thermal stability in limestone. Geol. Soc. Lond. Special Publ. **151**, 27–42 (1999).

[36] G. J. Borradaile, Viscous magnetization, archaeology and Bayesian statistics of small samples from Israel and England. Geophys. Res. Lett. **30**, 2003GL016977 (2003).

[37] G. Pullaiah, E. Irving, K. Buchan, D. Dunlop, Magnetization changes caused by burial and uplift. Earth Planet. Sci. Lett. **28**, 133–143 (1975).

[38] A. P. Roberts, C. R. Pike, K. L. Verosub, First-order reversal curve diagrams: A new tool for characterizing the magnetic properties of natural samples. J. Geophys. Res. Solid Earth **105**, 28461–28475 (2000).

[39] M. Brown, FORCme: Application of machine code and adaptive smoothing within python for the rapid processing of First Order Reversal Curves. Zendo. 10.5281/zenodo.19636066. Accessed 6 May 2026.

[40] E. Hassul *et al*., Geomagnetic field intensity during the first millennium BCE From Royal Judean Storage Jars: Constraining the duration of the Levantine Iron Age Anomaly. *Geochem. Geophys. Geosyst.* **25**, e2023GC011263 (2024).

[41] Y. Vaknin , Pre-excavation identification and dating of Iron Age Destruction Events. Archaeol. Prospect. **32**, 1021–1030 (2025).

[42] L. Bar-Sovik *et al*., Testing the accuracy of paleointensity estimates using experimental pottery assemblages. *J. Geophys. Res. Solid Earth* **131**, e2025JB033568 (2026).

[43] L. Tauxe, H. Staudigel, Strength of the geomagnetic field in the Cretaceous Normal Superchron: New data from submarine basaltic glass of the Troodos Ophiolite. Geochem. Geophys. Geosyst. **5**, 2003GC000635 (2004).

[44] H. Tanaka, T. Kobayashi, Paleomagnetism of the late quaternary Ontake volcano, Japan: Directions, intensities, and excursions. Earth Planets Space **55**, 189–202 (2003).

[45] A. Savitzky, M. J. E. Golay, Smoothing and differentiation of data by simplified least squares procedures. Anal. Chem. **36**, 1627–1639 (1964).

[46] J. D. A. Zijderveld, A. C. Demagnetization of Rocks: Analysis of Results, Methods in Paleomagnetism (Chapman and Hall, 1967).

[47] Y. Vaknin , Reconstructing biblical military campaigns using geomagnetic field data. Proc. Natl. Acad. Sci. U.S.A. **119**, e2209117119 (2022). 36279453 10.1073/pnas.2209117119 PMC9636932

[48] T. Sato , Paleomagnetism reveals the emplacement age of tsunamigenic coral boulders on Ishigaki Island, Japan. Geology **42**, 603–606 (2014).

[49] Y. Vaknin, B. Cych, J. S. Gee, L. Tauxe, Viscous remanent magnetization (VRM) authentication method for archaeological artifacts. Magnetics Information Consortium (MaGIC). https://earthref.org/MagIC/20694. Deposited 27 July 2026. 10.1073/pnas.2620397123 PMC13552657 42691086

[50] L. Tauxe , PmagPy: Software package for paleomagnetic data analysis and a bridge to the Magnetics Information Consortium (MagIC) Database. Geochem. Geophys. Geosyst. **17**, 2450–2463 (2016).

