# Supplementary material for: Viscous remanent magnetization authentication method for archaeological artifacts
Source: Proc Natl Acad Sci U S A. 2026 Sep 3;123(36):e2620397123. doi: 10.1073/pnas.2620397123 (PMC13552657; doi:10.1073/pnas.2620397123)
Supplement: Supplementary file 1 [file pnas.2620397123.sapp.pdf]

PNAS

Supporting Information for

Viscous Remanent Magnetization (VRM) Authentication Method for Archaeological Artifacts

Yoav Vaknin, Brendan Cych, Jeffrey S. Gee and Lisa Tauxe

Corresponding Author: Lisa Tauxe
E-mail: ltauxe@ucsd.edu

This PDF file includes:

Supporting text
Figs. S1 to S24
Tables S1 to S4
SI References

Supporting Information Text

Extended Methods. We used the Single Domain Comprehensive Calculator (SDCC) to simulate paleomagnetic experiments in archaeological samples containing single domain (SD) particles (1, 2). This package combines micromagnetic single domain energy barrier calculations with a kinetic model, which extends classic Stoner-Wohlfarth-Néel theory to account for magnetocrystalline anisotropy in particles with different shapes. The particle size and shape distribution for both the 'ancient' and 'modern' simulated artifacts are presented in Fig. S1. This distribution was chosen to ensure that particles with a wide range of shapes made a reasonable contribution to the magnetization, which is shown in Fig. S2. This is important because particles of different shapes will have their VRM removed at slightly different temperature ranges, leading to a small "curvature" in the Zijderveld plot (2, see Fig. 4). The choice of shape and size distribution for the model is supported by our own First-Order Reversal Curve (FORC) measurements, carried out on the actual archaeological and modern specimens used in this study (see Extended Results below). The FORC results are consistent with a dominant single-domain-like population in our own material, an empirical, sample-specific line of evidence independent of the grain-size distribution assumed in the model. This is also broadly consistent with the wider literature on archaeological ceramics. Atkinson and King (3) surveyed a worldwide collection of archaeological pottery sherds (3400 BC-1910 AD) and found that magnetic particle sizes were "skewed to smaller sizes, with sherds mostly having a large superparamagnetic or a stable single domain fraction". In general, they claim that "The magnetic mineralogy of ancient ceramics is often suited to archaeointensity determinations (the magnitude of the ancient geomagnetic field) because of the high fraction of stable single domain particles". They further concluded that the grain size distribution in ancient ceramics "often straddles the superparamagnetic–stable single domain boundary, implying a sensitivity to the acquisition of viscous remanence", directly supporting the physical basis of our approach. We also note a more complex picture in the literature. Kostadinova-Avramova and Kovacheva (4) examined synthetic fired clays and found, via Day-plot and Lowrie–Fuller analysis, a substantial multi-domain-like signature persisting even after firing to 700°C, with grain-size estimates in the micron range. We believe this most likely reflects the difference between bulk domain-state proxies (which capture the total magnetic mineral assemblage, including material that may contribute little to a stable remanence) and the specific sub-population of grains that controls TRM/VRM acquisition, which our FORC data suggest is fine-grained in our samples. However, we acknowledge this indicates real variability across clay sources and firing conditions, which can limit the applicability of our method in some cases. One methodological difference may help explain this discrepancy. The synthetic samples of Kostadinova-Avramova and Kovacheva were prepared directly from fresh, wet clay taken straight from the deposit, pushed through an aluminum mold and cut into cubic samples, with no crushing, sieving, or water-based purification applied. Potters, in antiquity as well as today, typically process raw clay before use, commonly by crushing and dry-sieving it, and in many traditions by levigation, a water-based settling technique that mechanically separates finer clay-sized particles from coarser sand-, silt-, and rock-fragment material (5). We ourselves used crushed and sieved clay when preparing experimental pottery for this study, as described by Bar-Sovik et al. (6): "The natural clays were crushed and sieved to produce a homogeneous, workable material." Because such processing preferentially removes coarser material, plausibly including larger detrital magnetic grains before the clay is shaped and fired, it offers one possible explanation for why the unprocessed synthetic samples of Kostadinova-Avramova and Kovacheva might carry a coarser magnetic grain-size population than genuine archaeological pottery. We are not aware of any study that has directly measured the effect of clay processing on magnetic grain size, so we present this as a plausible hypothesis rather than a demonstrated mechanism.

The simulation modeled the experiment as a sequence of temperature-time-field steps that incorporated thermocouple data from the pottery firing experiment using traditional kilns (6) and from the Scripps paleomagnetic ovens (see Figure S3). We also used the SDCC to simulate a hysteresis loop for a handful of these same particles to investigate the range of predicted coercivities for these particles to compare with hysteresis data.

To constrain the distribution of coercivities in our samples, we carried out FORC experiments on representative specimens. The FORC measurements were carried out using a Lakeshore 8604 vibrating sample magnetometer (VSM). We first crushed each sample using a mortar and pestle. In order to prevent the material from shifting during the vibrations of the VSM, we compressed the crushed material into capsules with cotton on both sides of the capsule. Each FORC consists of 600 equally spaced curves using the following parameters: $0 < B_c < 100$ mT, -50 mT$< B_u < 50$ mT, Saturation field=1 T and averaging time=0.15 s. In order to process the FORC data we used FORCme v1.0.1 software package (7). This software package does not require the user to guess what the most appropriate smoothing factor is for the specific data. For the purposes of this paper, we chose a smoothing factor of 0.5.

For VRM removal, we used the thermal demagnetizer in the Scripps Paleomagnetic Laboratory. This instrument includes five type E thermocouples. Each of these thermocouples has an offset which has been optimized for temperatures in the range of $500 - 600$°C, for the common paleomagnetic experiments. This is done by thermally demagnetizing a set of standard samples to minimize the lateral variations in temperature at these high temperatures. For the experiment described here, we require relatively low heating temperatures with high precision. To assess this, we first measured the accuracy and precision of the thermal demagnetizer when heating to 50°C, 60°C, 100°C, 150°C and 200°C. Each of the two Scripps thermal demagnetizers has a central 18" oven and two flanking 6" ovens, used to minimize lateral temperature variation within the 12" central portion that contains the samples. Each of these two flanking 6" ovens has its own controlling thermocouple (green and orange lines in Fig. S18). An additional thermocouple is placed in the middle of the 18" oven, and thus best represents the temperature of the samples (blue line in Fig. S18). Two additional monitoring thermocouples are located about 1.5" from the ends of the 18" oven and thus their readings do not represent the actual temperature of the samples (dashed gray lines in Fig. S18). Therefore, small deviations (either high or low) of these monitoring thermocouples do not indicate deviations of the sample temperature.

The accuracy of type E thermocouples is 1.7 to 2.0°C over the range of temperatures that we used in the current experiment. Both the initial tests and the actual experiments show that the specimens placed in the 12" central portion of the ovens were heated to the desired temperature ±2.5°C (Fig. S18).

The VRTC code workflow is as follows:

1. Imports a measurement file in the MagIC format (https://earthref.org/MagIC). Measurements should normally consist of at least 17 data points per specimen: the NRM and demagnetization steps until more than 20% of the initial moment is removed (for most specimens 200°C is sufficient).
2. Limits the data to the range before the magnetic moment drops below 80% of the initial moment. The moment referenced in the VRTC, and in the 80%-of-NRM threshold, is the raw measured moment magnitude at each demagnetization step, not the vector-difference-sum (VDS) moment.
3. For every demagnetization step, calculates DANG (8, 9) values using the pmag.domean() function from PmagPy (10) package (available at: https://github.com/PmagPy/PmagPy), based on a group consisting of the current step and the four following steps. The DANG is the angle between the best fitting line through the selected data and the direction of the vector from the the center of mass of the selected data to the origin.
4. Adds to the DANG and normalized magnetic moment data points at every 1° interval, by linear interpolation between every two measured points. This is necessary in order to apply Savitzky-Golay filtering.
5. Smooths normalized magnetic moment curve and DANG curve with Savitzky-Golay filtering (11), with a window length of 121. The Savitzky-Golay filter is applied using the savgol filter function imported from SciPy library in Python (12).
5. Selects VRM removal temperature based on local maxima in DANG second derivative which is above a threshold of $5e^{-4}$ and DANG under a threshold of 17°. Since the DANG is the angle between the direction of the component and the origin, the drop in the DANG means that in the current and the following steps, the demagnetization removes the main magnetic signal, the TRM. In other words, at this point and on the VRM component in the remaining magnetic signal is not significant.
6. Selects VRM removal temperature based on moment second derivative minima under a threshold of $-1.2\mathrm{e}^{-5}$ with persistent negative first derivative and DANG under a threshold of 17°. Since the VRM component is usually in a different direction from the TRM, its demagnetization usually does not result in a significant drop in the total moment. Therefore, such a drop marks the point where the VRM component in the remaining magnetic signal is not significant.
7. Takes average of the two VRM removal temperatures.
8. Plots results.

The VRM component itself can appear noisy in the Zijderveld diagrams, likely reflecting the overprinting of several distinct VRM components acquired under different conditions over time. Once the VRM is fully removed, the data typically settle into a clean, linear trajectory converging to the origin, and we considered this a reasonable stopping point for identifying the VRM removal temperature. To test this directly, and challenge the choice of the 80% cutoff, we reran our VRTC on the dataset using progressively relaxed cutoffs: 70%, 60% and 50% of the initial NRM remaining, instead of the original 80%. For most specimens, there are only slight differences in the VRT, not influencing their classification as ancient or modern (relative to the 112°C threshold). For five specimens, changing the cutoff influenced their classification and they are presented in Table S4. At the original 80% remaining cutoff, every specimen's classification matches its known ancient/modern designation. Relaxing the cutoff to include more of the NRM leaves most specimens' classifications unchanged, but where it does change, the effect usually runs in one direction: a known-modern specimen is misclassified as ancient and only once the reverse. At 70%, two specimens change appreciably: SHYE14Ds (115.5°C → 102.0°C, ancient → modern) and SM03s (84.0°C → 169.0°C, modern → ancient). SHYE14Ds is already discussed in the manuscript as a borderline case, where the VRM was likely recorded, by chance, in a direction similar to the TRM; this is a known limitation of the method and relaxing the cutoff makes it worse. SM03s is a genuine false positive introduced by relaxing the cutoff. At 60% and 50%, the problem gets worse, with additional known-modern specimens (T6GO1Bs and T6MA1Cs at 60%; T4GO1As at 50%) also seeming ancient. We believe this is because, at these more relaxed cutoffs, significant drops in moment due to reasons other than VRM removal, most likely the Curie temperature of a subset of the magnetic minerals present, become part of the data window and are mistaken for the VRM signature.

Extended Materials. We used 26 pottery artifacts which we knew were modern before the experiments (Table S1). Four of these modern artifacts were purchased in several shops (Fig. 1H-I) in the Souvenir Market in the old city of Jerusalem (sample names begin with SM for Souvenir Market). The shop owners sell these artifacts as souvenirs and not as ancient pottery. Furthermore, the prices of these artifacts (∼8USD each) and the fact that there are many almost identical ones (Fig. 1H) clearly indicate that these are indeed modern artifacts. The names of all other modern 22 pottery samples begin with the letter 'T' followed by a number (1-6) representing one of the six kilns used to fire every object. Kilns T1 (Fig. 1A) and T2 (Fig. 1B) were two different types of traditional kilns constructed by Bar-Sovik et al. (6) in order to fire modern pottery vessels (Fig. 1C-D) in a manner similar to the ancient ones. They prepared these vessels from local clays which they collected in different locations in the southern Levant. In the four other kilns (T3-T6) we fired seal impressions which we created using a seal made by Rachel Pelta (Fig. 1E). Kilns T3 and T4 are standard electric ceramic kilns used in two modern ceramic workshops in Jerusalem. In kiln T3, a J230 Kiln made by L and L Kiln MFG Inc., we fired the seal impressions to 950°C. In kiln T4 (Fig. 1G), a KM822 kiln made by Skutt Ceramic Products Inc., we fired the seal impressions to 990°C. Kilns T5 and T6 are electric laboratory kilns smaller than T3 and T4. In kiln T5, a F48010 Muffle Furnace made by Thermolyne, we fired the seal impressions to 990°C. In kiln T6, a PA36B furnace made by Bifa, we fired the seal impressions to 810°C. Before firing in all six kilns, the

pottery vessels or seal impressions were dried at room temperature for at least several days. The temperature in the different kilns was raised gradually over 9-13 hours, as commonly done when firing ceramics in order to avoid cracks. The ceramics were removed from the different kilns when their temperature decreased below 30°C.

Bar-Sovik et al. (6) collected six types of clay in different locations in the southern Levant. For this research we used three of these types of clay: Golan (GO), Mamshit (MA) and Motza (MZ1). The advantage of these clays is that we know precisely where they were collected. However, we assume that forgers might purchase local clays instead of collecting and sifting their own clays. Therefore, in addition to these three types of clay and only for the seal impressions which were fired in kilns T3-T6 did we use two other types of clay that are sold for the use of potters. Both these types of clay originate, according to the manufacturers, from the Motza formation. One of these types (marked here as MZ2) was sold several decades ago by "Moshe-ceramic supply store" in Netanya. According to the shop owners it came from the vicinity of Motza, a neighborhood in western Jerusalem. The second type (marked here as MZ3) is sold today and according to the suppliers, it comes from the Motza formation in the area of the Hebron mountains. For the type of clay used for each sample see Table S1.

In addition to the modern samples, we used 19 ancient pottery samples, all of which had been unearthed in excavations (Table S2). Fifteen of these samples are stamped handles (thus sample names begin with SH) of Royal Judean Storage Jars (13). The other four samples are ancient pottery sherds exposed at Tel Nisa, an archaeological mound in the Beth-Shean Valley (14). These sherds were re-fired during a conflagration that destroyed the site during the Iron Age, $\sim$ 2900 years ago (14). They were part of a matrix of fired and collapsed bricks. This collapsed material was unearthed roughly in the same orientation in which it cooled down after this conflagration and thus all the samples taken from the pottery sherds and from the mudbrick material yielded directions close to that of the ambient geomagnetic field, with some scatter due to movement after cooling (14). These samples were chosen for the current research in order to challenge the suggested method.

In addition, we used three artifacts of questionable authenticity (Fig. 5C-E) which had been confiscated, because of the illegal antiquities trade, by the Antiquities Theft Prevention Unit (ATPU) of the Israel Antiquities Authority (Sample names beginning with ATPU). When preparing two of these samples we noticed that they consisted of two parts: the original pottery and a restored part. The restored parts were made of material that looked like pottery by color but after sampling them we saw that, unlike most pottery samples, they were homogeneous on the inside and they were clearly not pottery. We carried out AF demagnetization on samples from these restored parts and they carried no TRM. Therefore, we conclude that the restored parts were not made of fired clay. We assume that they are made of some kind of bonding material, such as Plaster of Paris, mixed with color to make it look like pottery.

Extended Results. In order to shed light on the magnetic mineralogy of the samples, we used FORC diagrams (15, 16). Although the interpretation of FORC diagrams is qualitative, it is increasingly used in order to identify the domain state. The starting point for creating FORC diagrams is the multiple hysteresis measurements (shown in Figs. S4 to S7). These so-called "first order reversal curves" (16) are the magnetizations (M) whereby each curve ranges from some Ba (the lowest field for the FORC) to Bb (the highest) measuring the magnetization $M(Ba, Bb)$. The two axes of the FORC diagram are $Bu = (Ba + Bb)/2$ and $Bc = (BbBa)/2$. FORC density (ρ) is defined as a mixed second derivative (see e.g. (16) for details). In a FORC diagram, the FORC distribution (ρ) is plotted with B_u on the y-axis and Bc on the x-axis.

Uniaxial SD particles will have the highest density along the $Bu = 0$ line (e.g. 17, sample SHLI2 in Fig. S8,). This behavior, known as the "central ridge", is present in the vast majority of our specimens (Figs. S8-S11). This central ridge generally has coercivity values lying between 0 and 40 mT, which is consistent with predicted values from the SDCC simulations (see Figure S17), and a peak coercivity of $\sim$ 10 mT, shown in profiles of the FORC distribution along the central ridge (Figs. S13 - S16), which is consistent with small particles. Although a small number of specimens exhibit a "spreading out" of the FORC distribution from the central ridge along the B_u axis (e.g. specimen ATPU06 in Fig. S8), which is indicative of Single-Vortex particles (18), the majority of our specimens do not exhibit any spreading out.

Another feature that is diagnostic of single-domain particles is a negative peak in the FORC distribution in a region close to the $B_c = 0$ axis with $B_u < 0$ (17, 19). This feature is subtle in most of the FORC diagrams, and is most easily seen by looking at FORC density profile plots, which are profiles across the FORC distribution, along the line $B_c = 5$ mT (Figs. S12 - S16). This behavior is not present in all specimens, but occurs frequently (e.g. specimen SHYL16B in Fig. S13).

To test the ability of the VRTC to identify authentic artifacts younger or older than 1000 years and forgeries older than 6 months, we ran a dedicated sensitivity analysis using the SDCC, varying the assumed VRM acquisition time and using the same grain-size/shape distribution as presented in Fig. S1. Regarding objects that acquired a VRM over more than 1000 years, we ran the SDCC with a 2500 year VRM acquisition time. The result is very similar to that of the 1000-year-old VRM. In both cases, the calculated $\mathrm{VRT_M}$ is 116°C. The $\mathrm{VRT_D}$ of the 2500-year-old object (114°C) is slightly higher than that of the 1000-year-old object (112°C). We also ran this simulation with a 100-year and a 10-year VRM acquisition time. We plotted the VRT (the average of the $\mathrm{VRT_M}$ and the $\mathrm{VRT_D}$) vs. acquisition time in Fig. S23. Notice that the time axis is logarithmic. Forgeries of pottery began more than 150 years ago (20). Figure S23 shows that the difference between a 500-year-old authentic object (VRT$\sim$109°C) and a 150-year-old forgery (VRT$\sim$100°C) is probably not significant enough in order to conclusively distinguish between them using the VRTC. However, if the forgery is 6 months old (VRT=66°C) than the VRTC will probably be able to distinguish between it and a 500-year-old authentic artifact, using a threshold lower than 112°C. In any case, the earlier the forgery and the younger the authentic artifact, the more limited our method is.

Some of the variability in VRTC will arise from the shape distribution of magnetic particles in the rock (1, 2). Figure S24 shows the thermoviscous stability of particles as a function of shape following Cych et al. (2). The point at which these curves cross the horizontal black line can be thought of as a proxy for the VRTC, although the reality is more complicated because

rocks contain mixtures of these particles. For particles with deviations from a perfect sphere or oblate disk >20%, the variation in this temperature is around ±10°C. In comparison with Fig. S23, this suggests that the precision of an age estimate made by the VRTC should be at least one order of magnitude in time. Additional outliers may be introduced by samples containing primarily non-single domain particles. Further research aiming to improve the precision of this method should focus on the effects of non-SD particles on VRTC, and better ways to obtain a quantitative estimate of the sample's magnetic particle distribution.

All data from this paper are available for the purposes of review here: https://earthref.org/MagIC/ 20695/25f824fc-7a51-4279-a6d3-c8c3433622ea. After publication, the URL will be: https://earthref.org/MagIC/20695. The python code used for analyzing the data in the MagIC dataset is available here: https://github.com/ltauxe/VRM-removal-temperature-notebook.

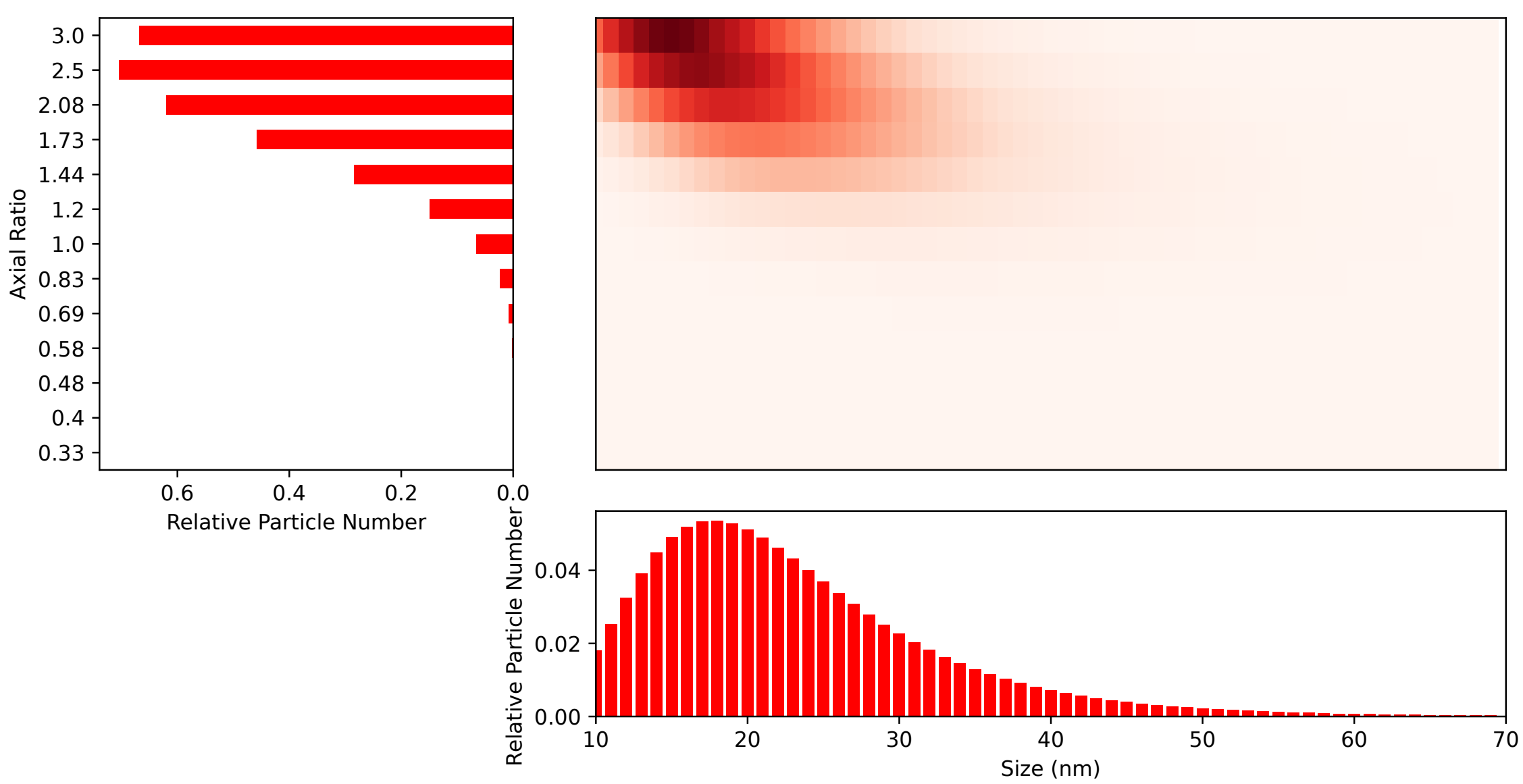

Fig. S1. The particle shape and size distribution (relative number of particles) of both the ancient and the modern simulated samples used for the SDCC micromagnetic modeling. All simulated particles were magnetite ellipsoids with the magnetocrystalline easy axis ($<1\,1\,1>$) aligned with the ellipsoid major axis. Axial ratios $>=1$ refer to the major/intermediate axis ratio, whereas axial ratios $<=1$ refer to the intermediate/minor axis ratio.

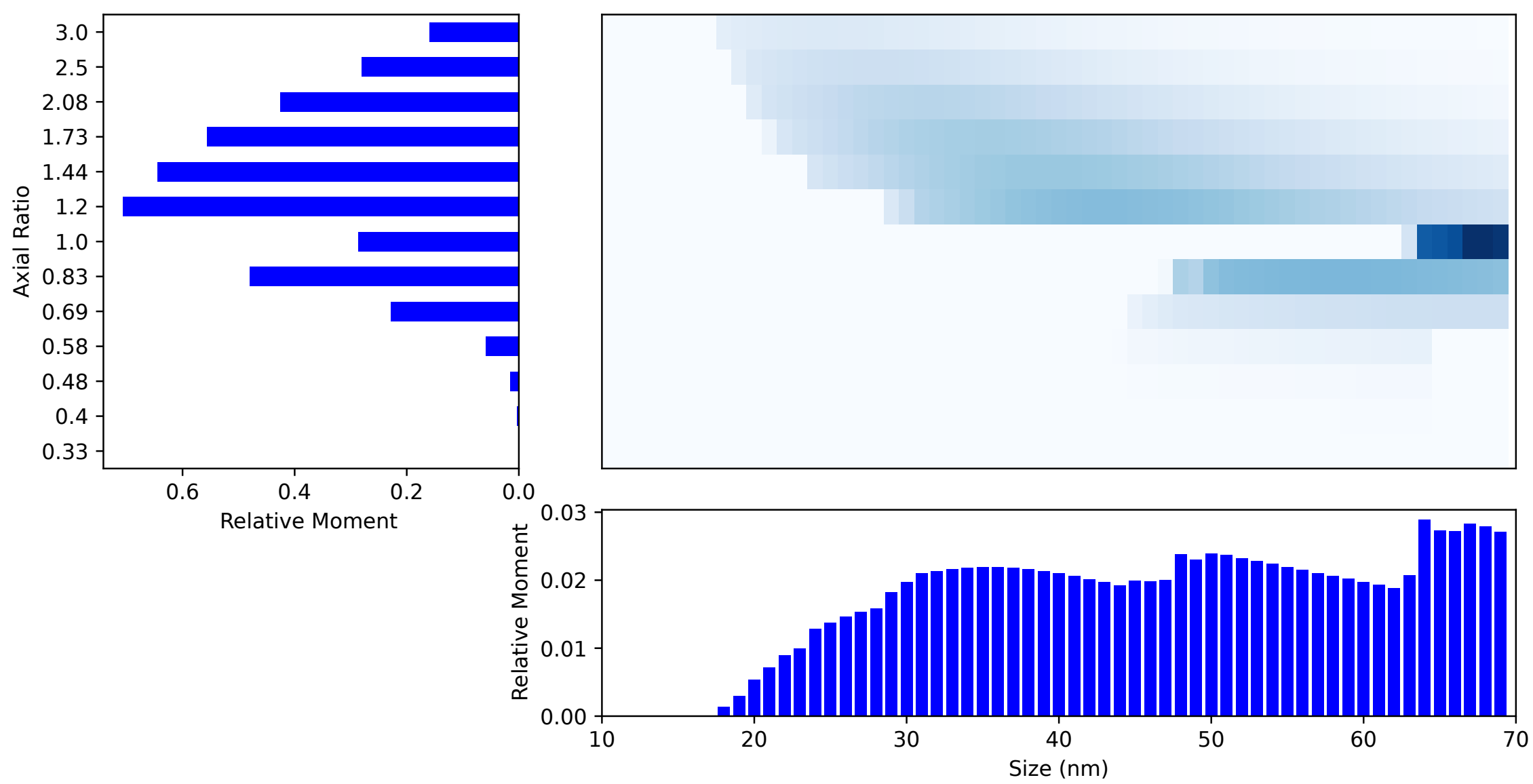

Fig. S2. Relative contribution to the NRM moment (for the simulation of a "modern" artifact) from the same distribution of particles in Fig. S1. Larger particles have much higher NRM magnitudes, despite being a much smaller fraction of the overall particles.

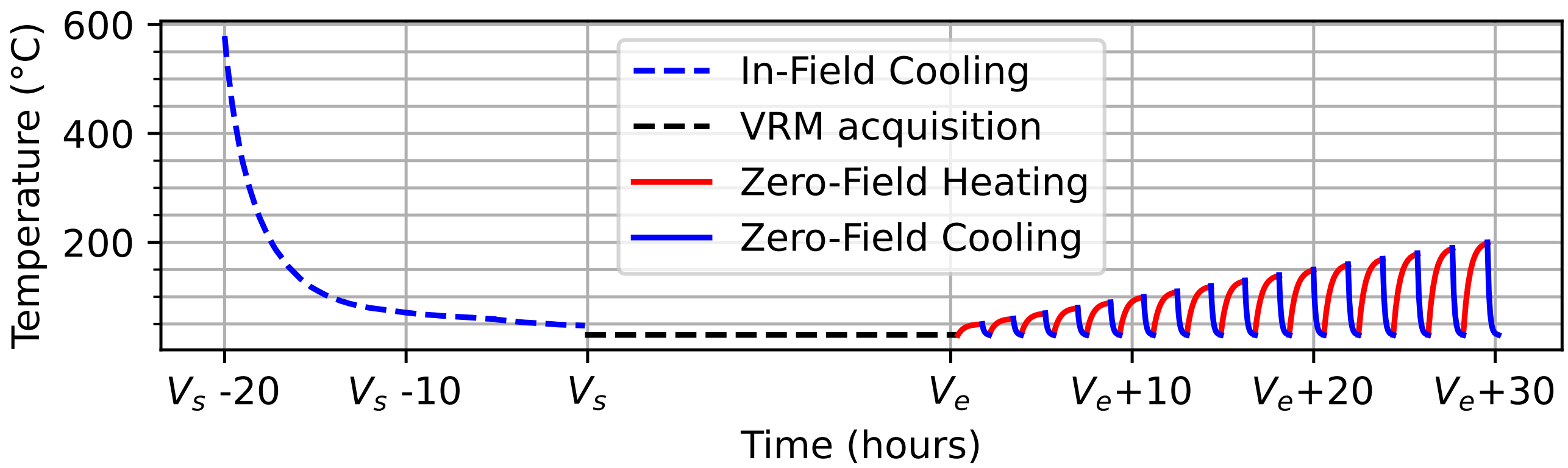

Fig. S3. Experimental protocol plotted as a function of temperature and time for both the simulations of "ancient" and "modern" pottery. Heating and cooling times are marked relative to the start of the VRM acquisition time V_s and the end of the VRM acquisition time V_e (not to scale in this figure). The VRM was acquired at 30°C.

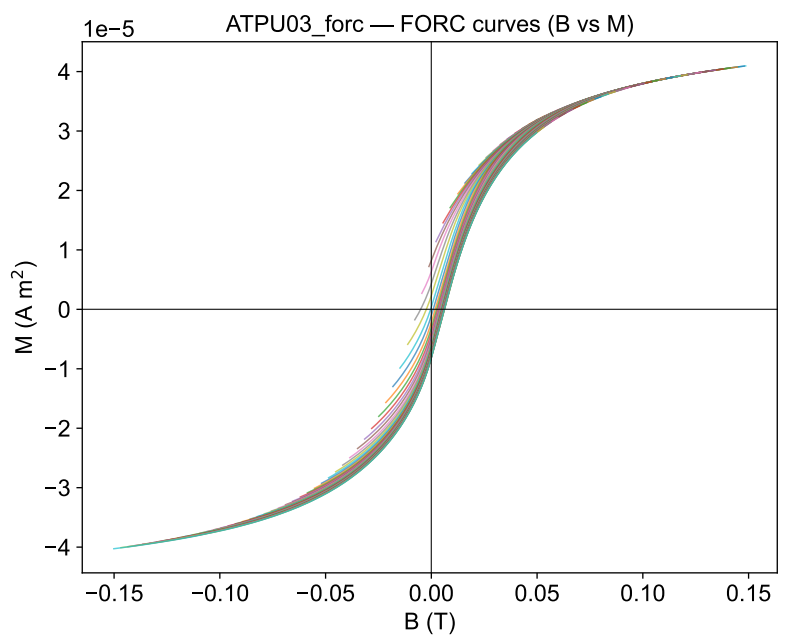

ATPU03_forc · FORC curves (B vs M).pdf

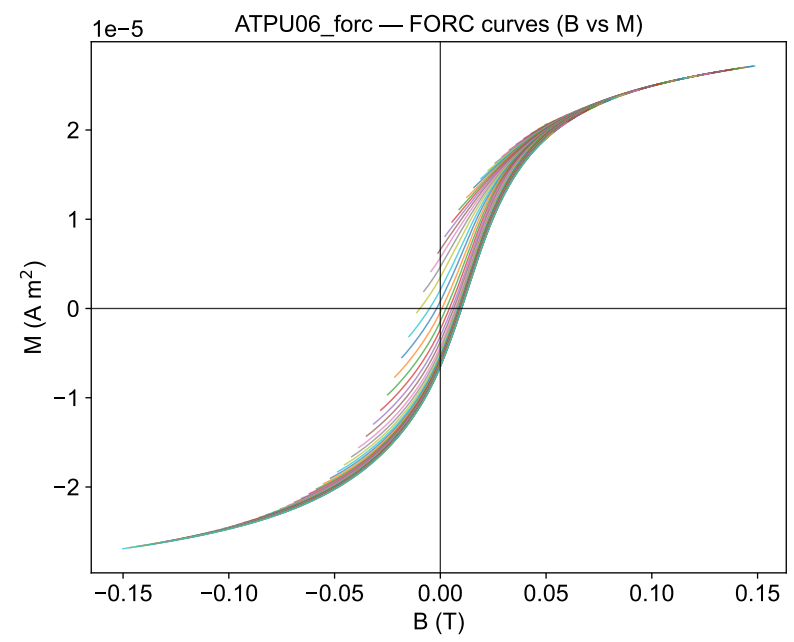

ATPU06_forc · FORC curves (B vs M).pdf

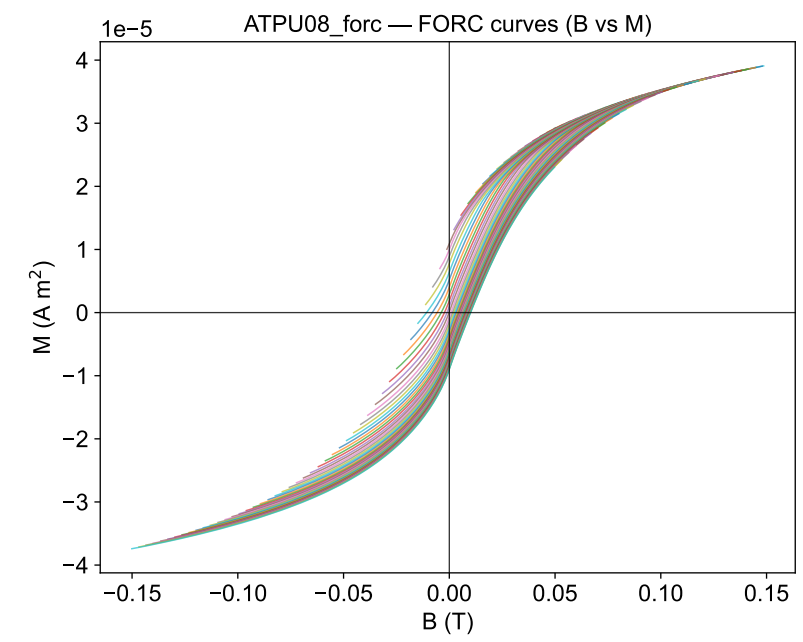

ATPU08_forc · FORC curves (B vs M).pdf

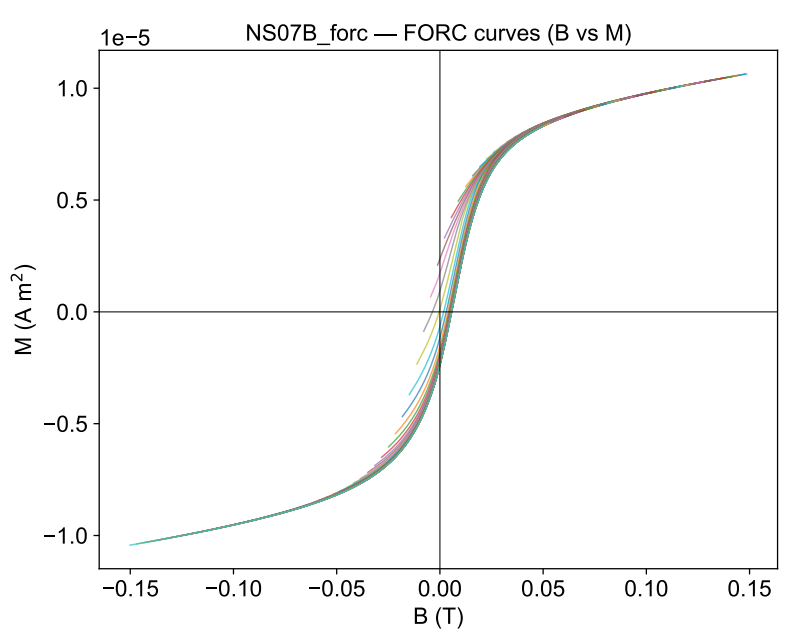

NS07B_forc · FORC curves (B vs M).pdf

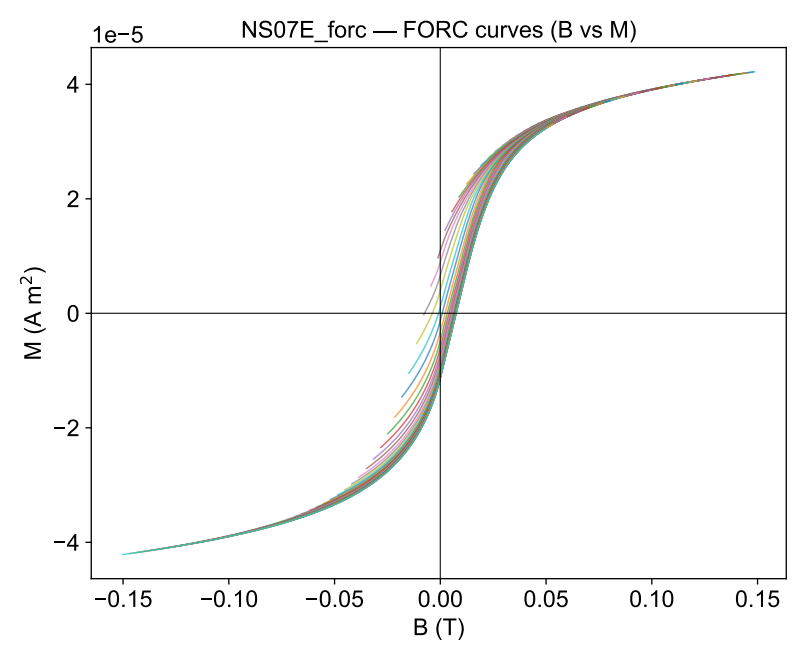

NS07E_forc · FORC curves (B vs M).pdf

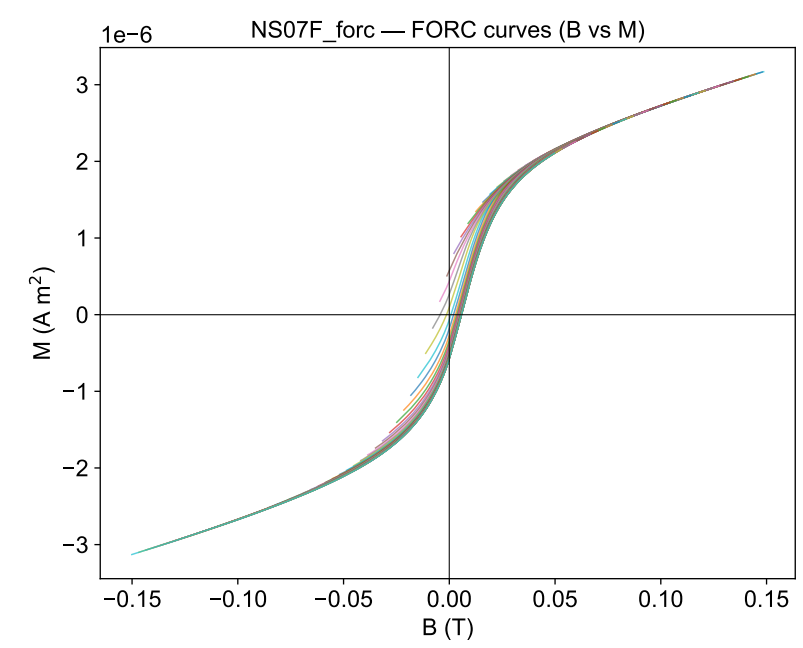

NS07F_forc · FORC curves (B vs M).pdf

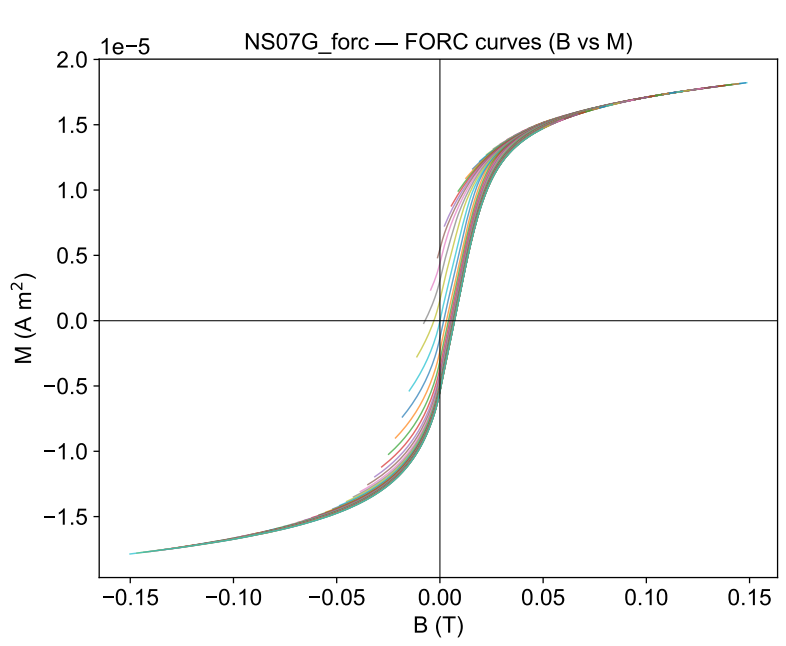

NS07G_forc · FORC curves (B vs M).pdf

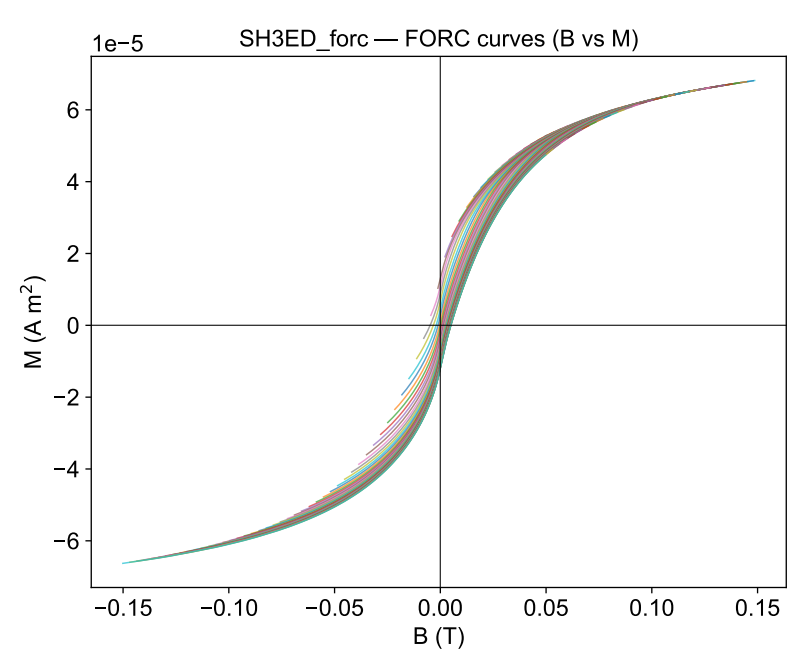

SH3ED_forc · FORC curves (B vs M).pdf

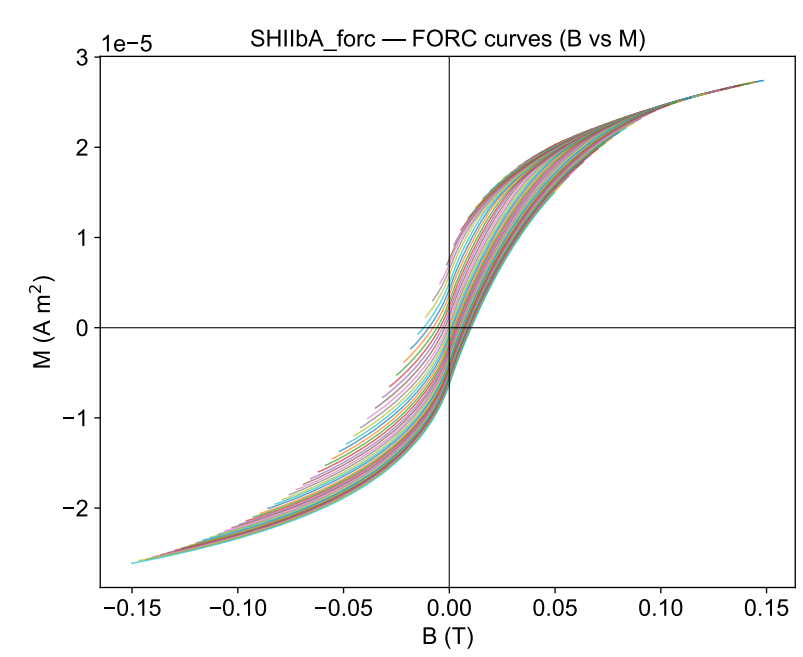

SHIIbA_forc · FORC curves (B vs M).pdf

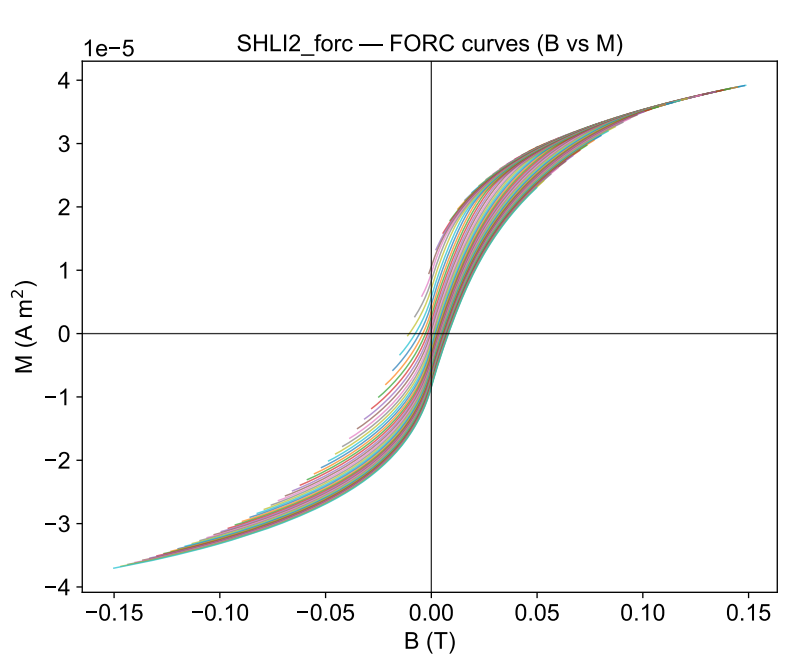

SHLI2_forc · FORC curves (B vs M).pdf

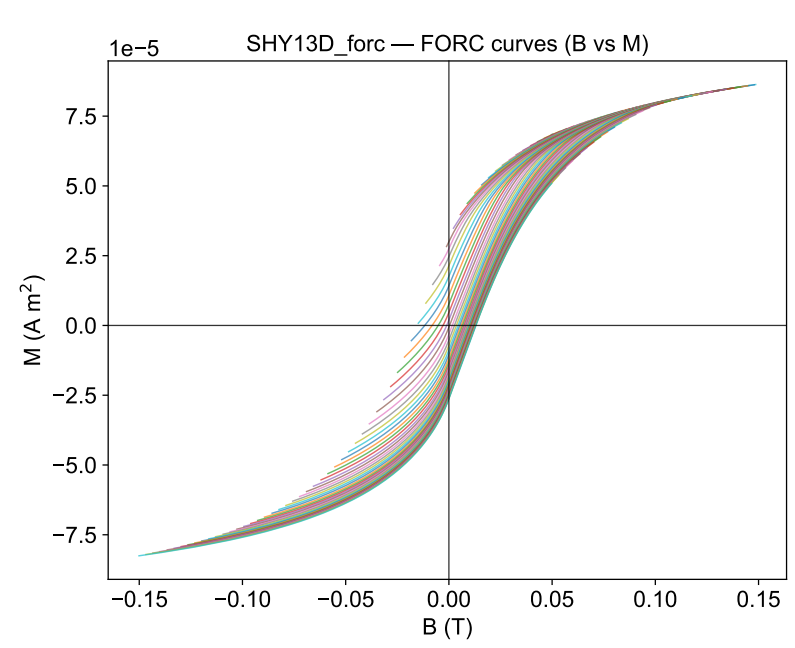

SHY13D_forc · FORC curves (B vs M).pdf

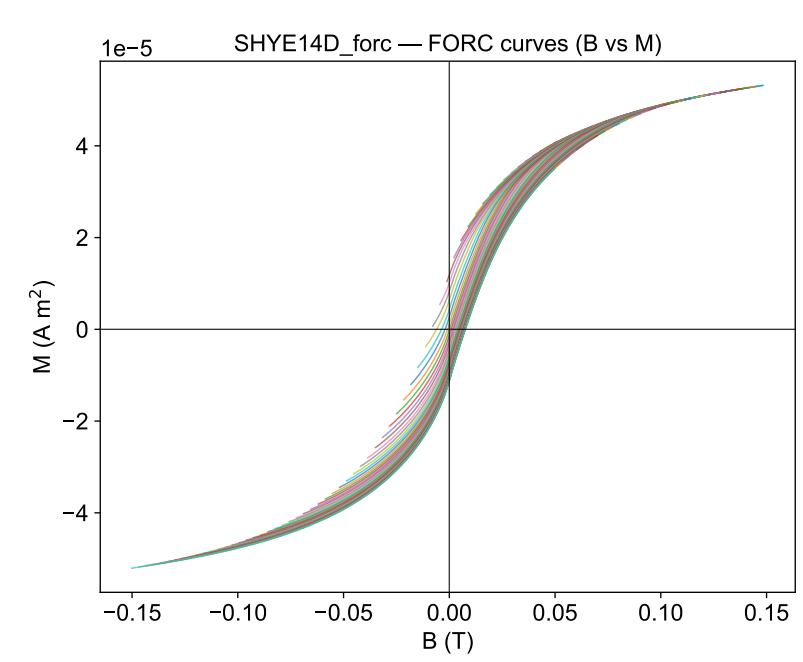

SHYE14D_forc · FORC curves (B vs M).pdf

Fig. S4. Hysteresis loops for ATPU03 (upper left) to SHYE14D (lower right). The horizontal axes are the applied field in the VSM in tesla and the vertical axes are the measured moment in Am^2. These plots were generated with the FORCme code (7).

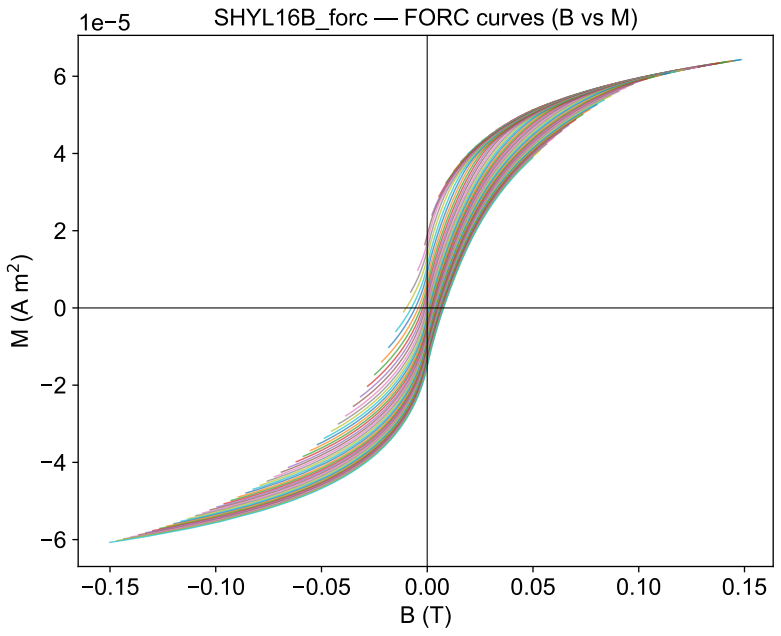

SHYL16B_forc · FORC curves (B vs M).pdf

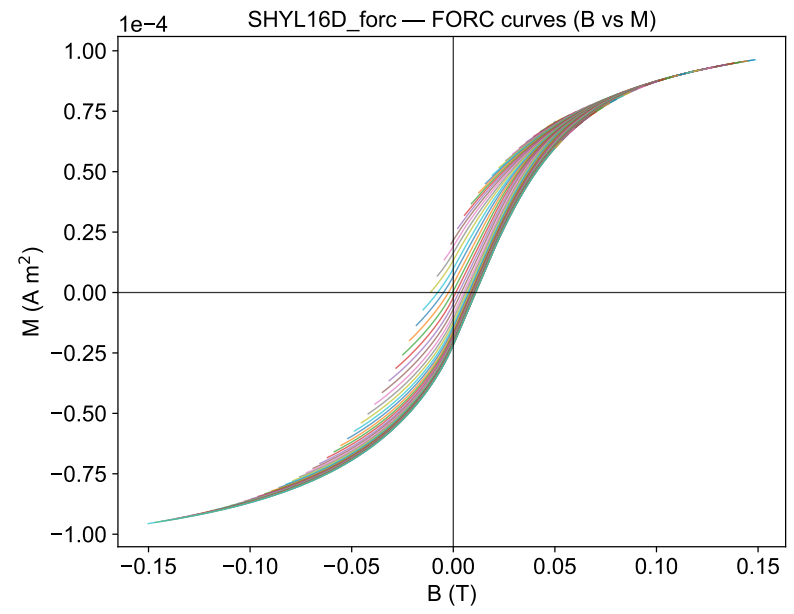

SHYL16D_forc · FORC curves (B vs M).pdf

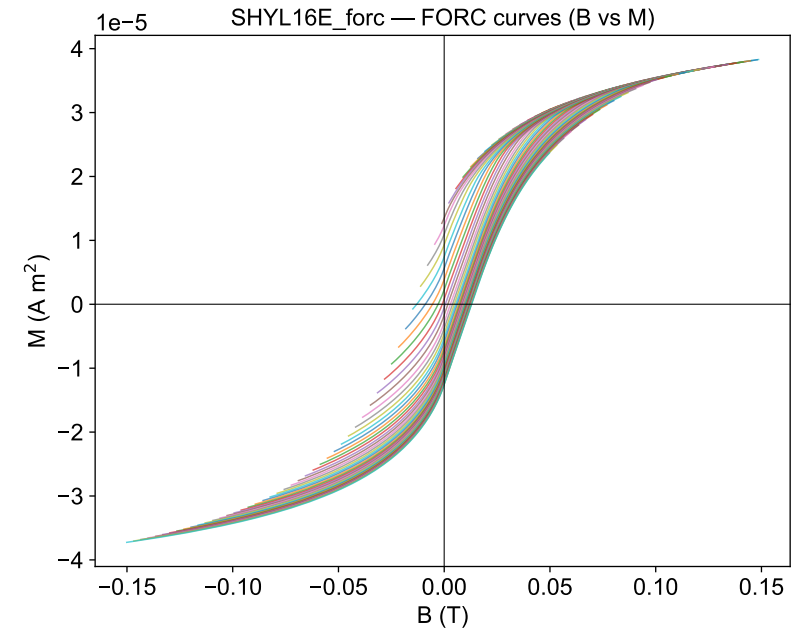

SHYL16E_forc · FORC curves (B vs M).pdf

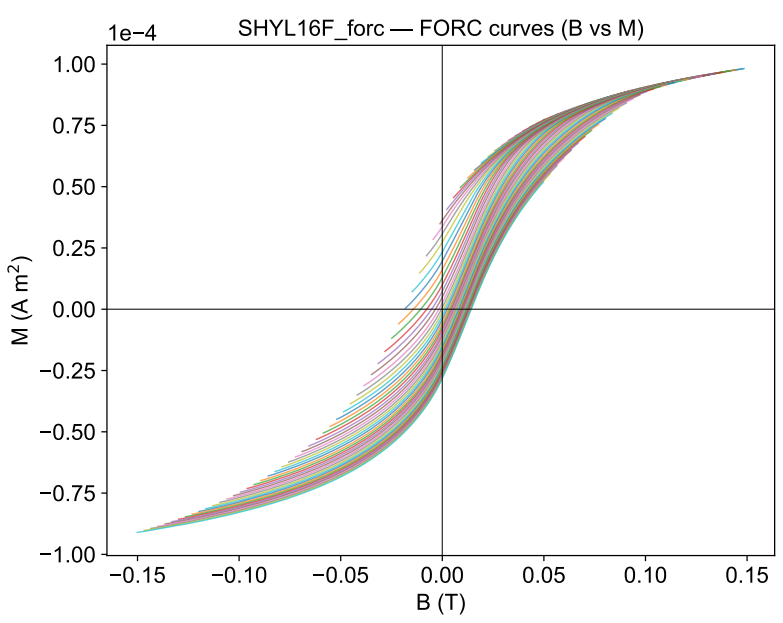

SHYL16F_forc · FORC curves (B vs M).pdf

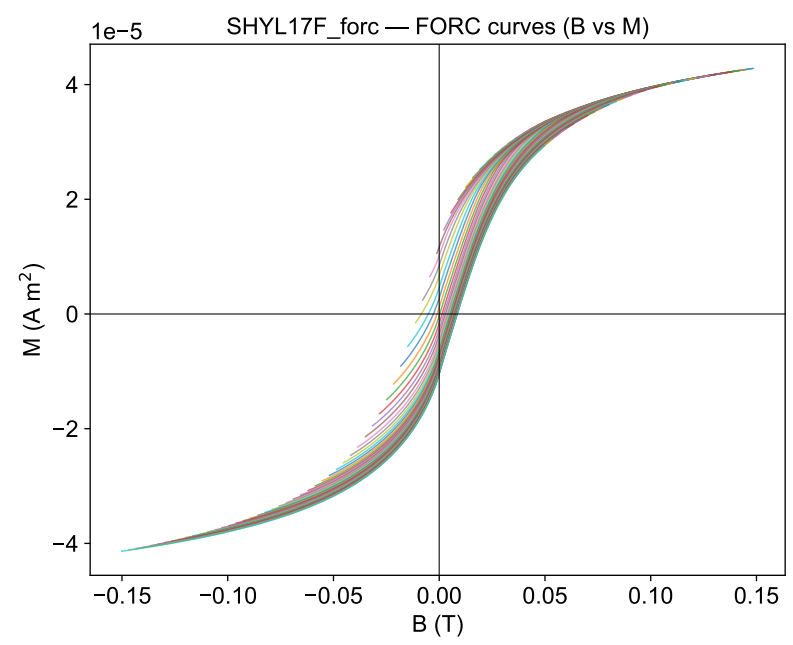

SHYL17F_forc · FORC curves (B vs M).pdf

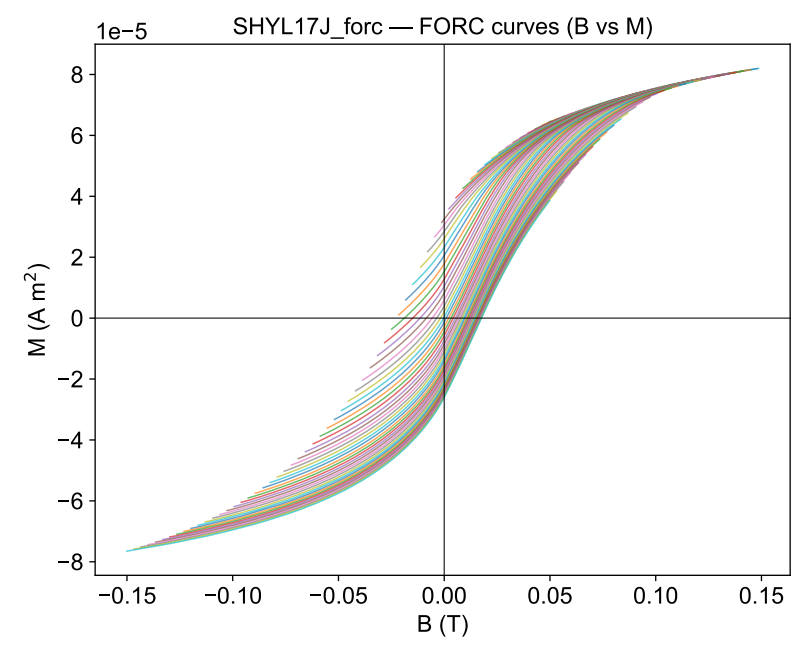

SHYL17J_forc · FORC curves (B vs M).pdf

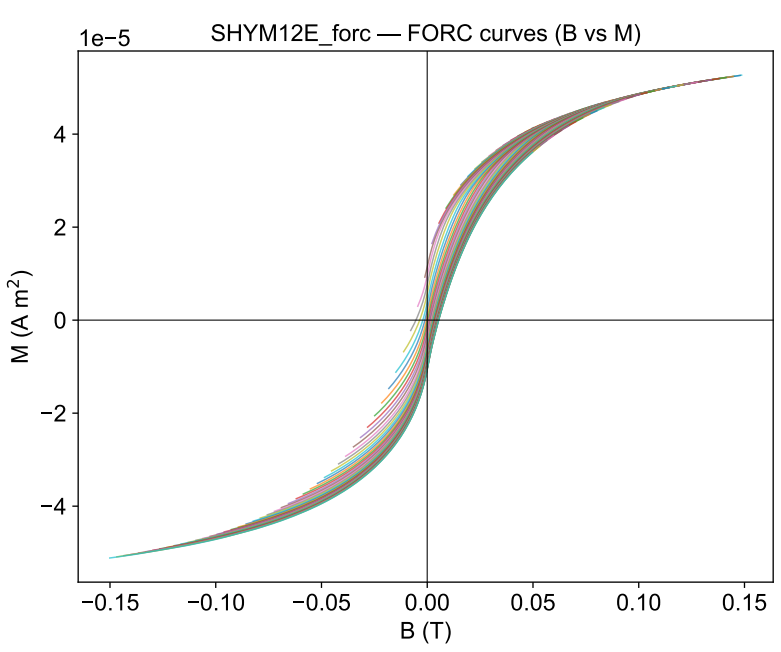

SHYM12E_forc · FORC curves (B vs M).pdf

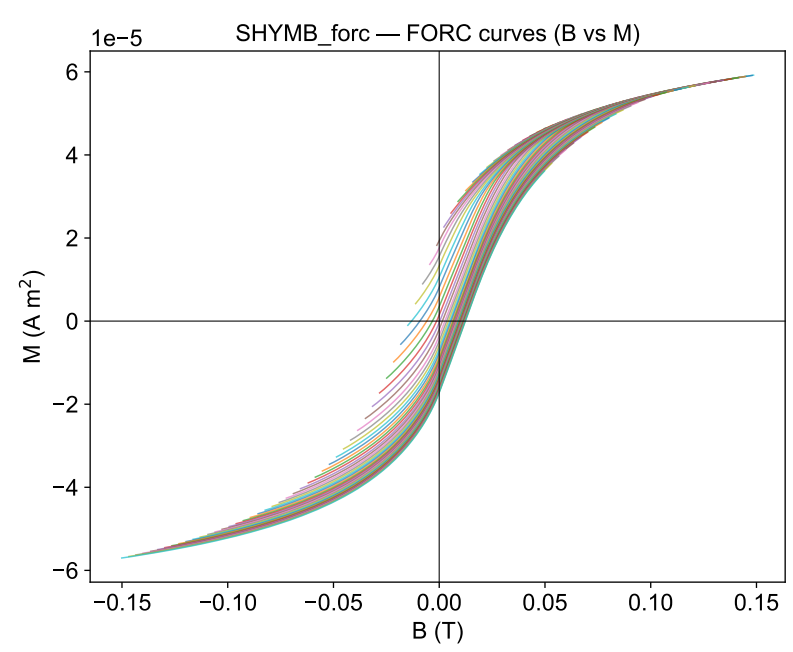

SHYMB_forc · FORC curves (B vs M).pdf

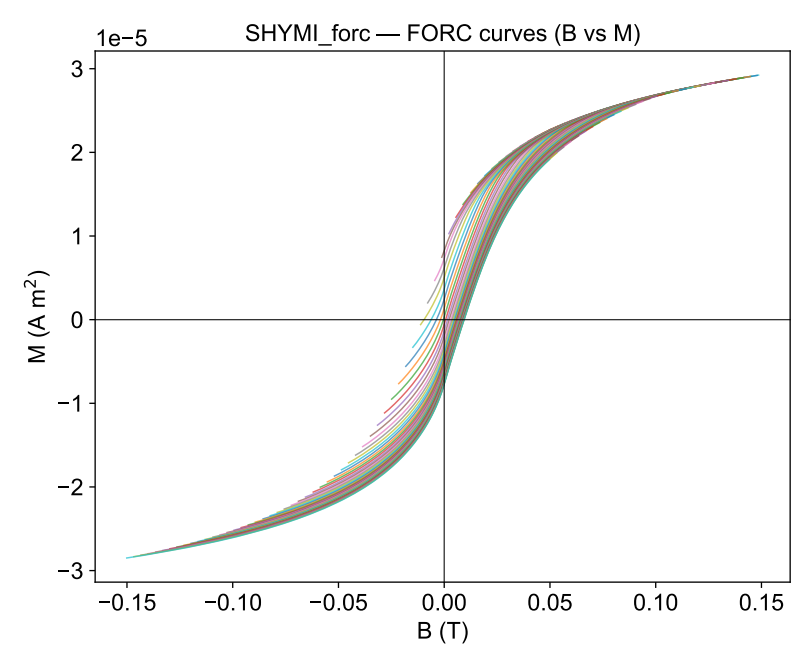

SHYMI_forc · FORC curves (B vs M).pdf

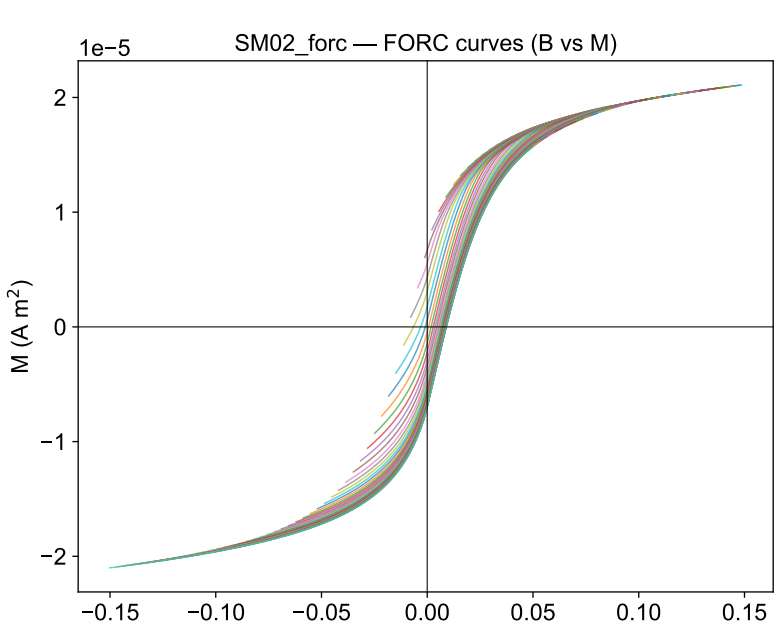

SM02_forc · FORC curves (B vs M).pdf

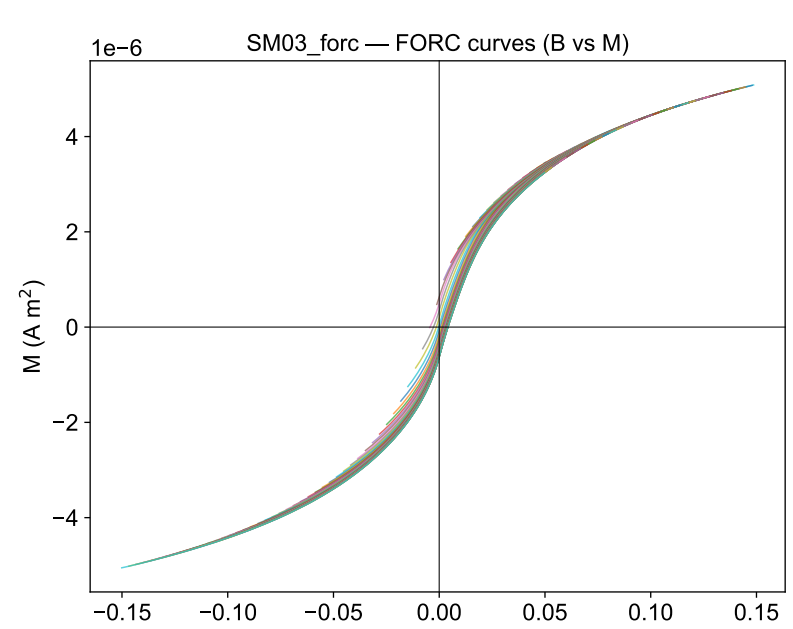

SM03_forc · FORC curves (B vs M).pdf

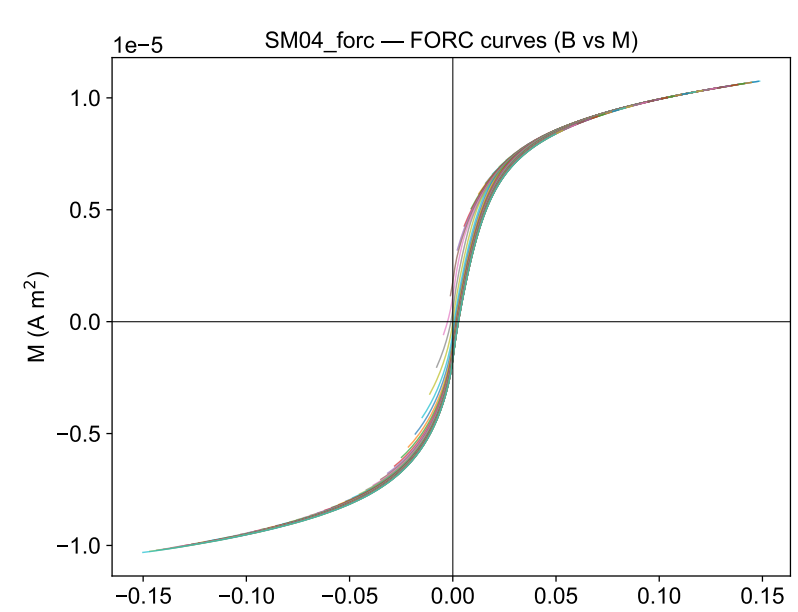

SM04_forc · FORC curves (B vs M).pdf

Fig. S5. Same as in S4 but for SHYL16B (upper left) to SM04 (lower right).

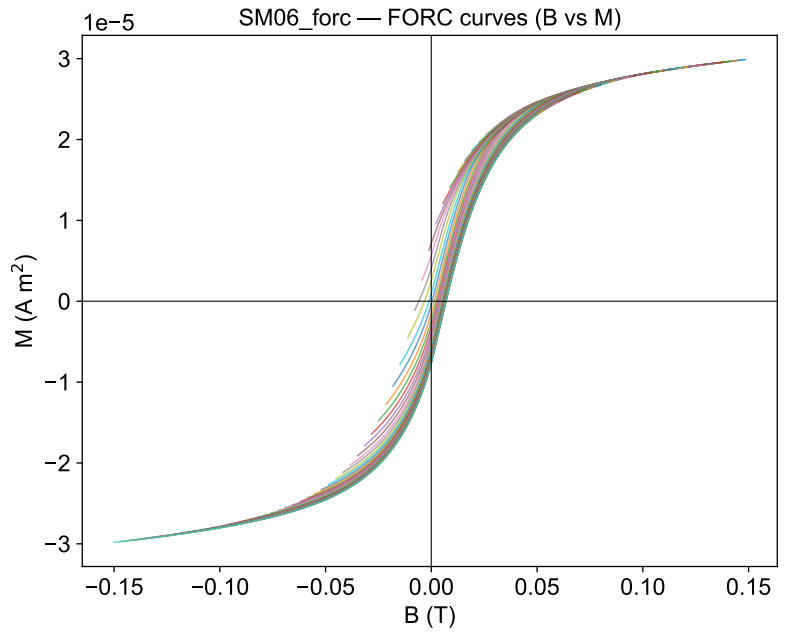

SM06_forc · FORC curves (B vs M).pdf

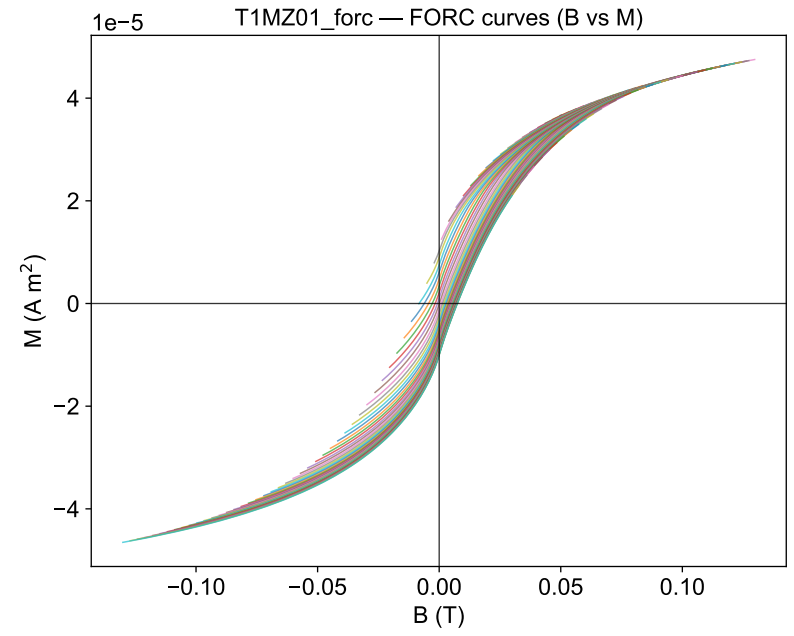

T1MZ01_forc · FORC curves (B vs M).pdf

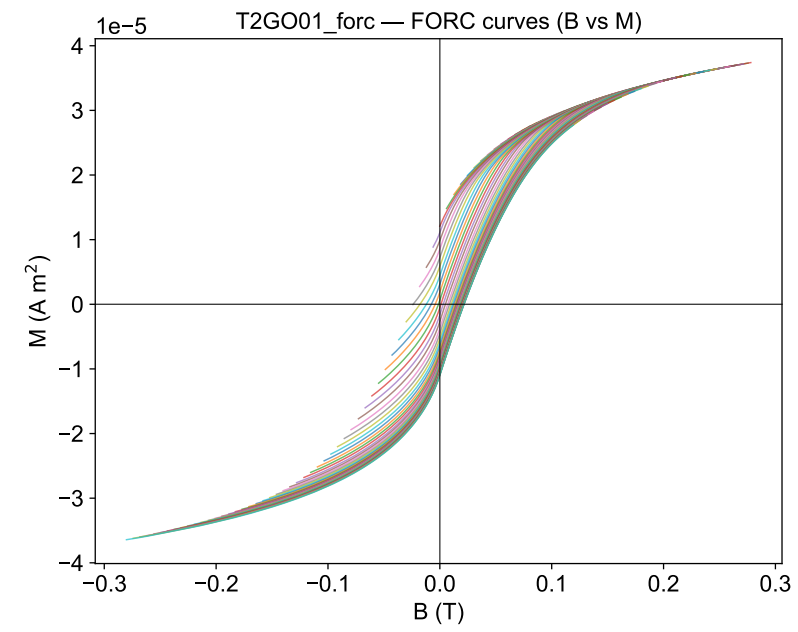

T2GO01_forc · FORC curves (B vs M).pdf

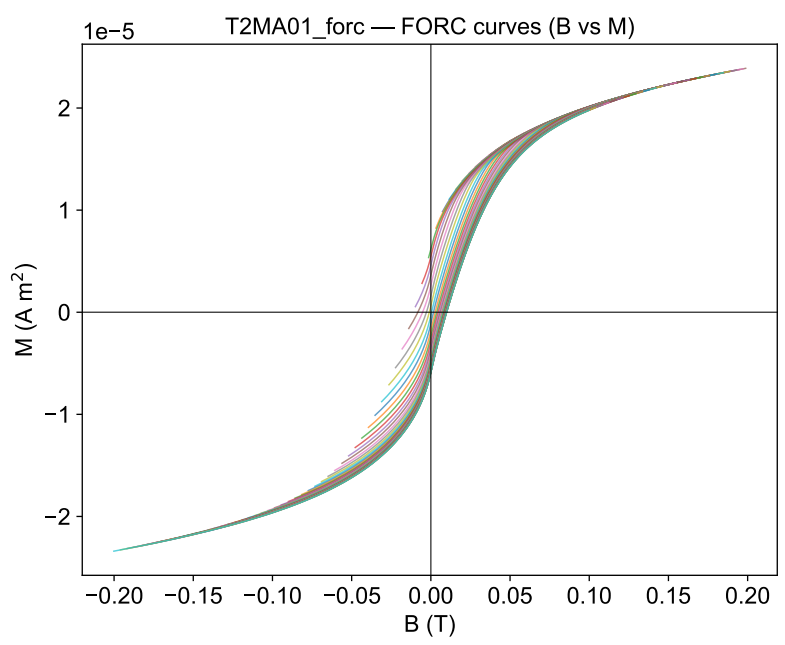

T2MA01_forc · FORC curves (B vs M).pdf

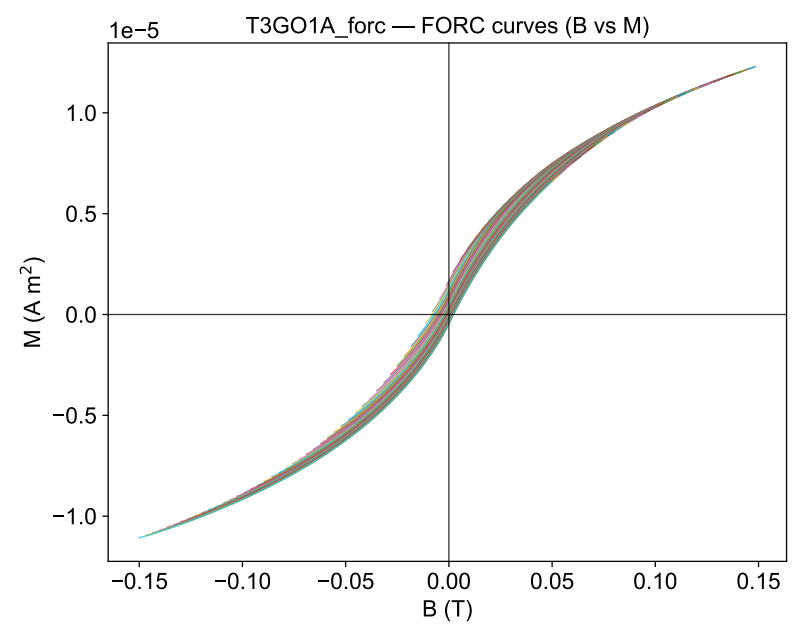

T3GO1A_forc · FORC curves (B vs M).pdf

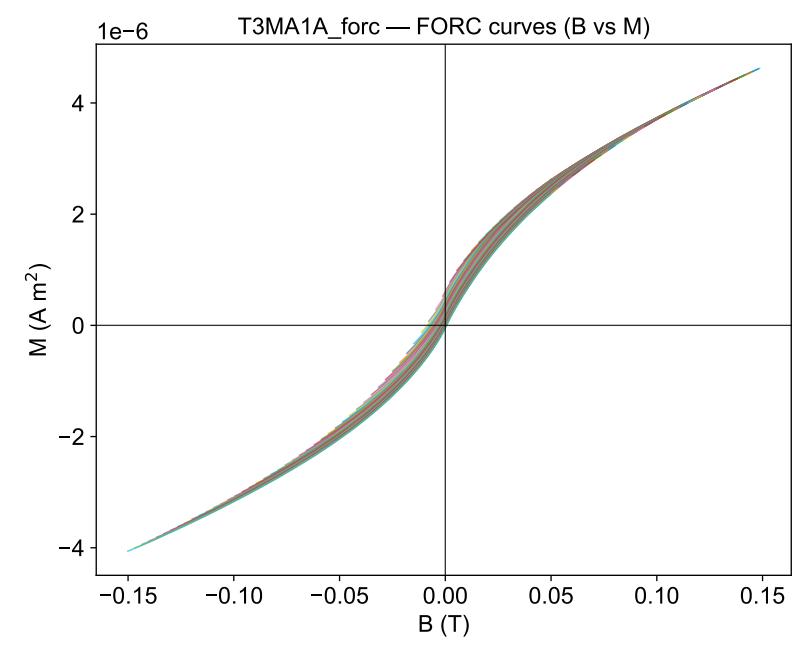

T3MA1A_forc · FORC curves (B vs M).pdf

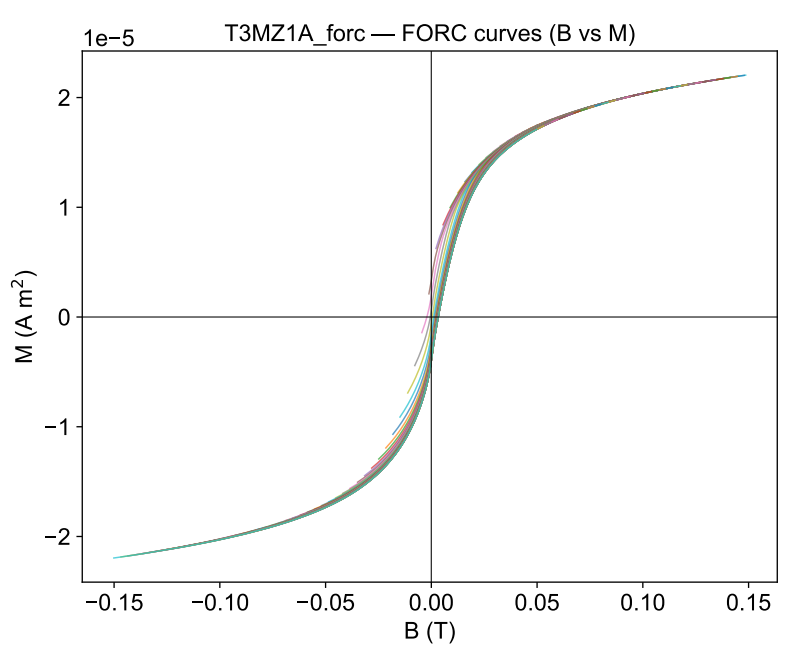

T3MZ1A_forc · FORC curves (B vs M).pdf

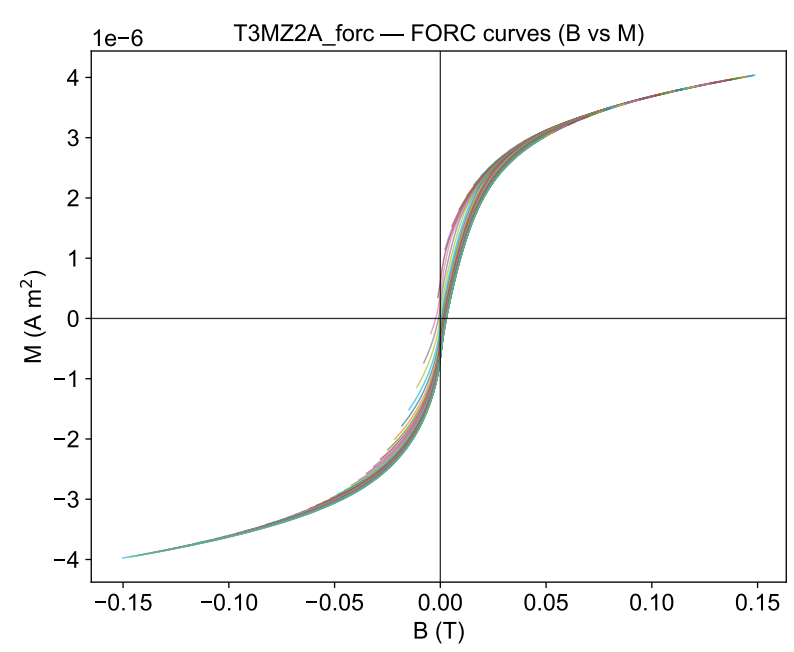

T3MZ2A_forc · FORC curves (B vs M).pdf

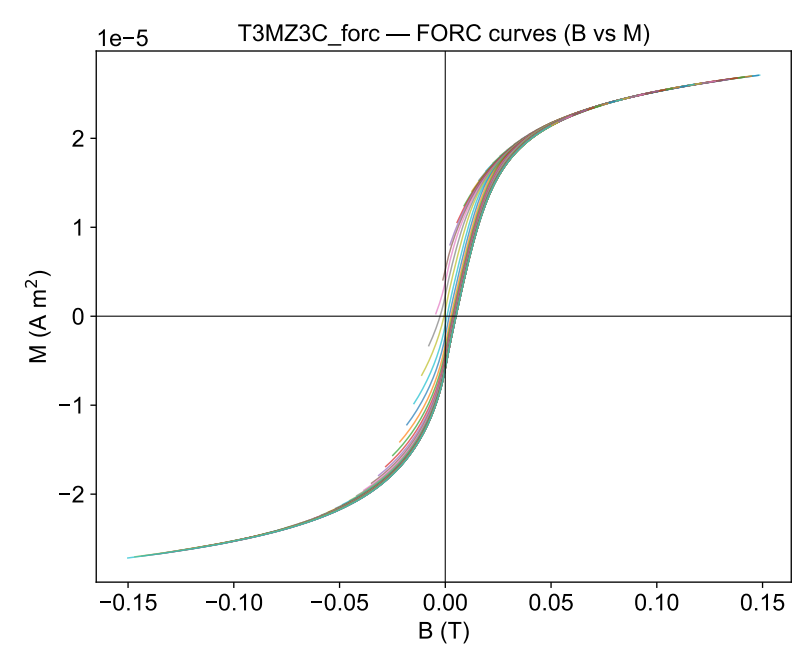

T3MZ3C_forc · FORC curves (B vs M).pdf

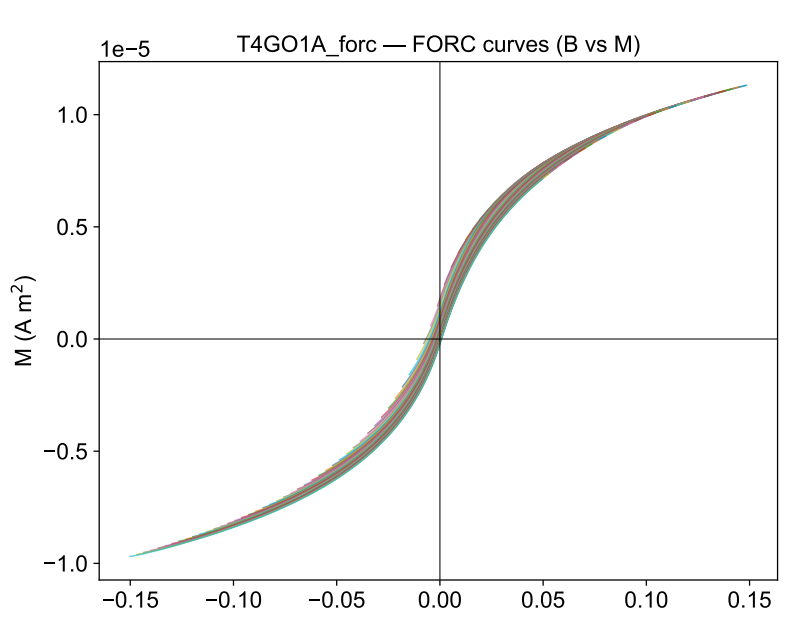

T4GO1A_forc · FORC curves (B vs M).pdf

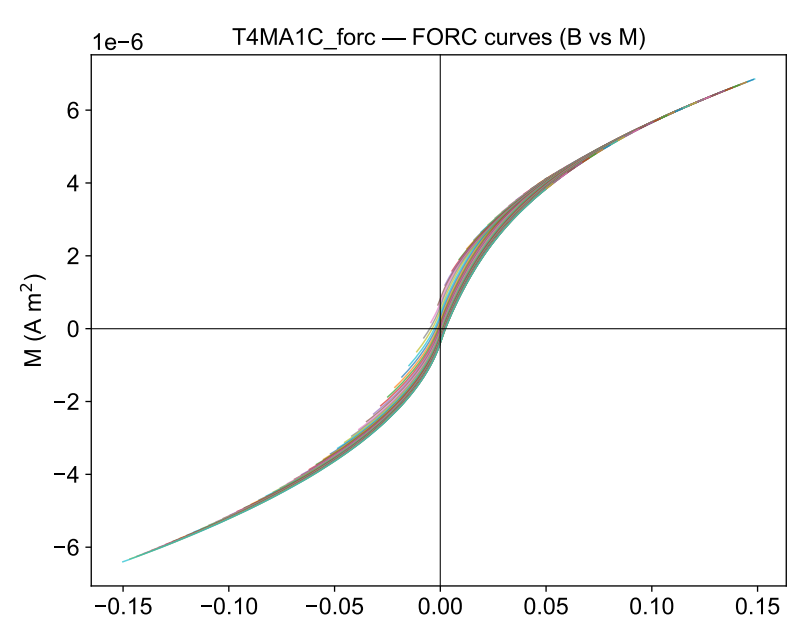

T4MA1C_forc · FORC curves (B vs M).pdf

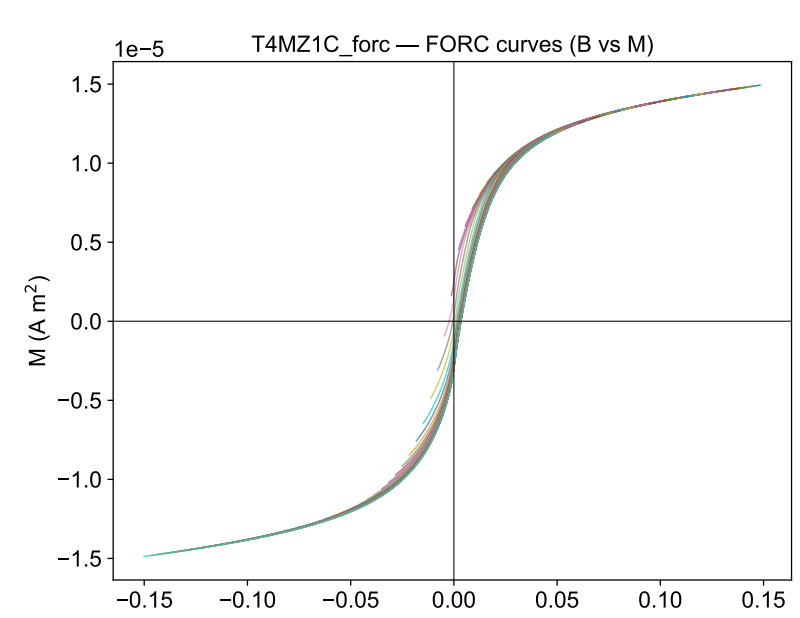

T4MZ1C_forc · FORC curves (B vs M).pdf

Fig. S6. Same as in S4 but for SM06 (upper left) to T4MZ1C (lower right).

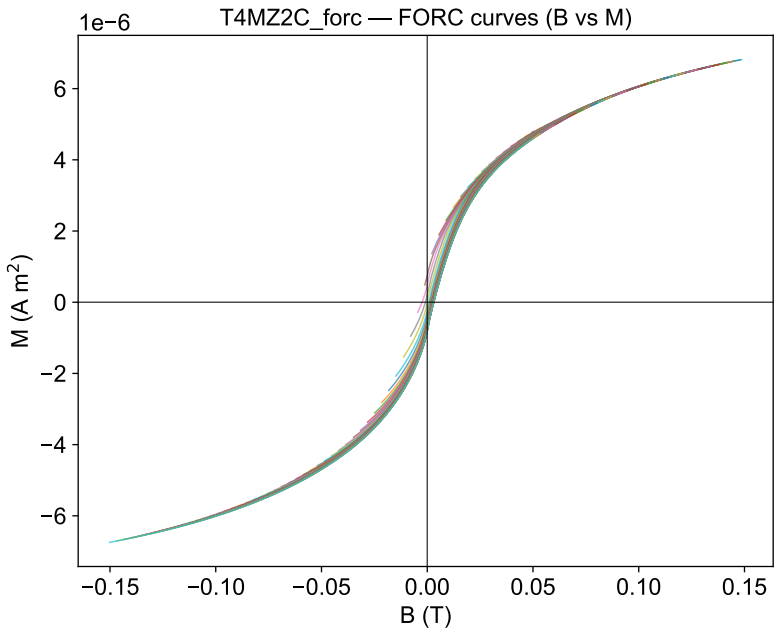

T4MZ2C_forc · FORC curves (B vs M).pdf

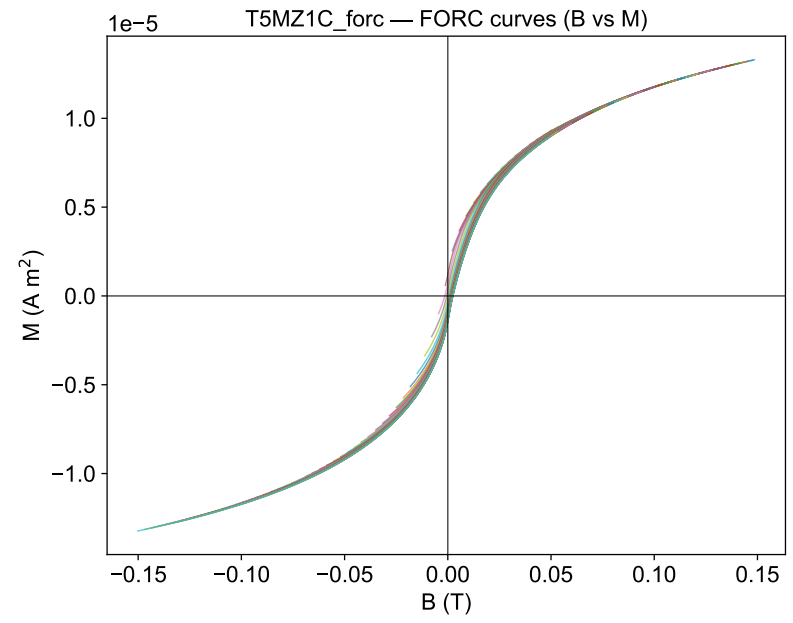

T5MZ1C_forc · FORC curves (B vs M).pdf

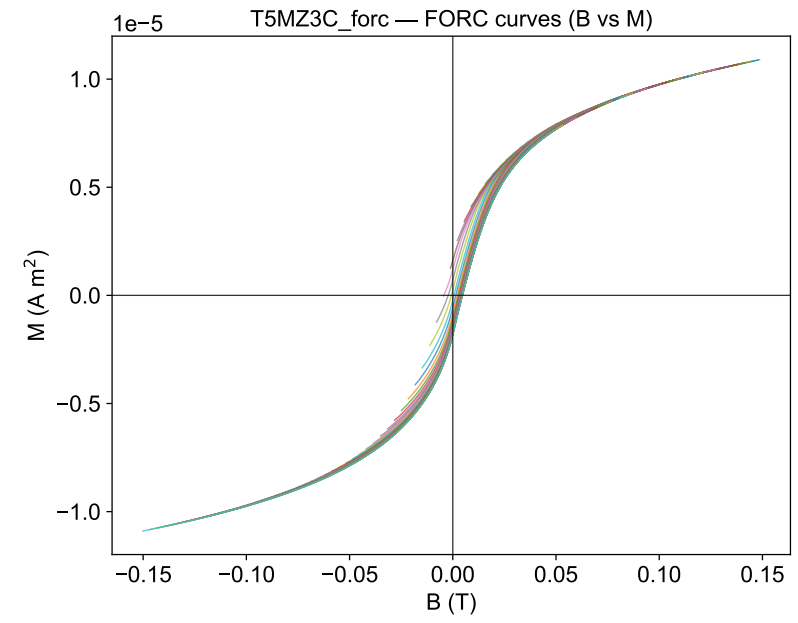

T5MZ3C_forc · FORC curves (B vs M).pdf

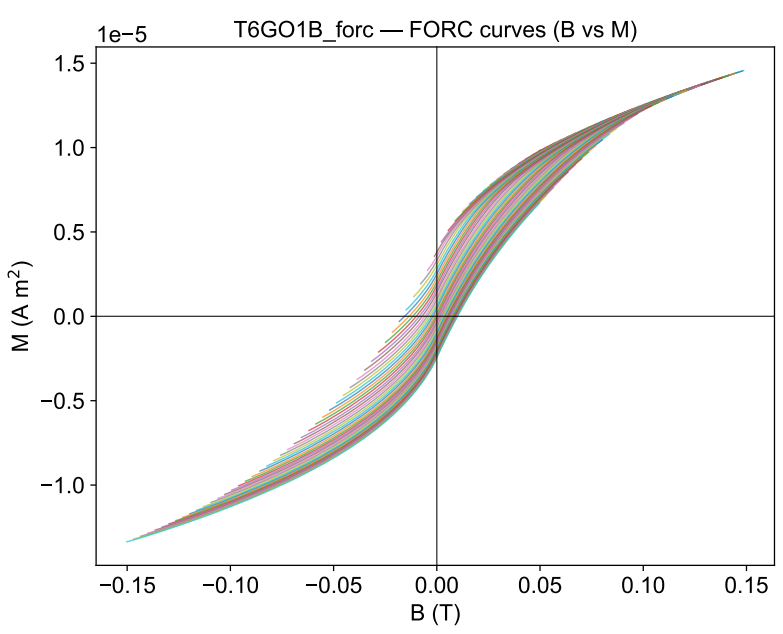

T6GO1B_forc · FORC curves (B vs M).pdf

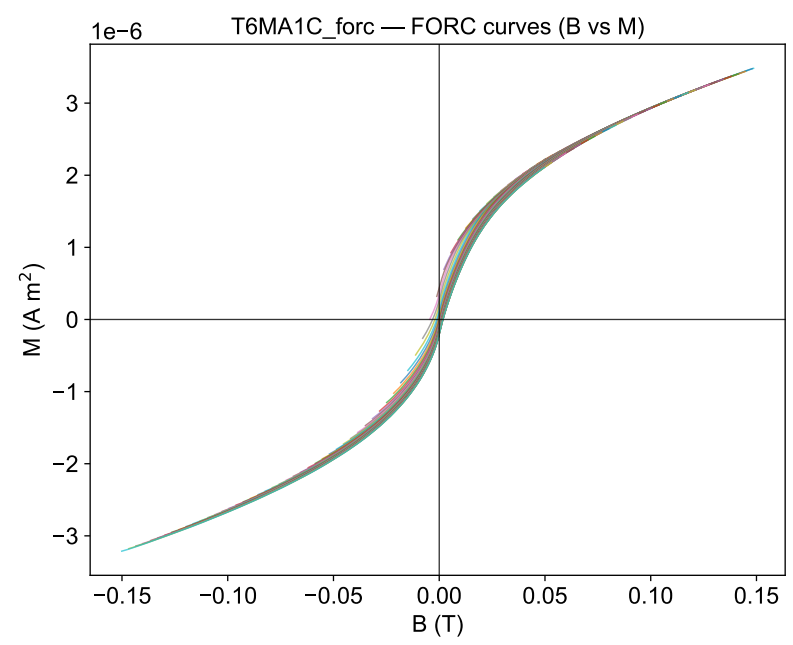

T6MA1C_forc · FORC curves (B vs M).pdf

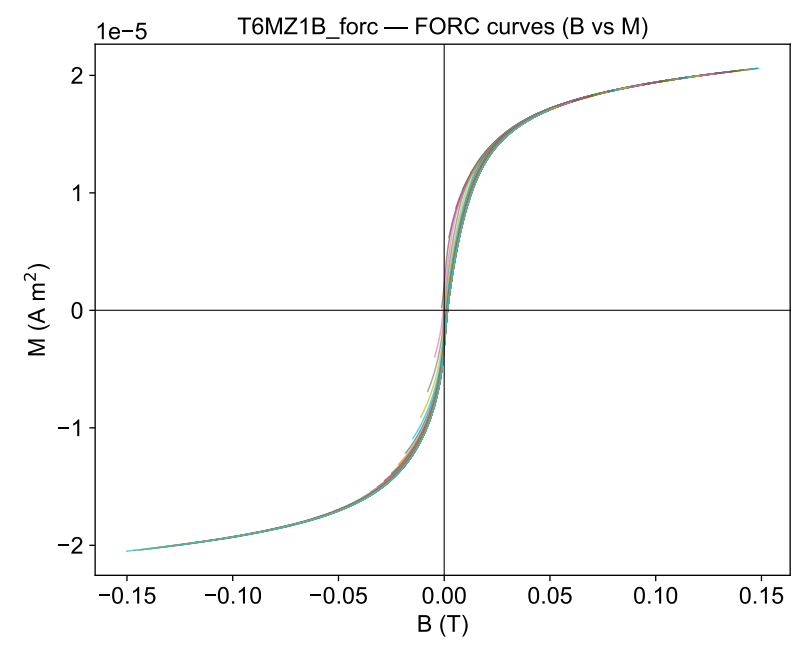

T6MZ1B_forc · FORC curves (B vs M).pdf

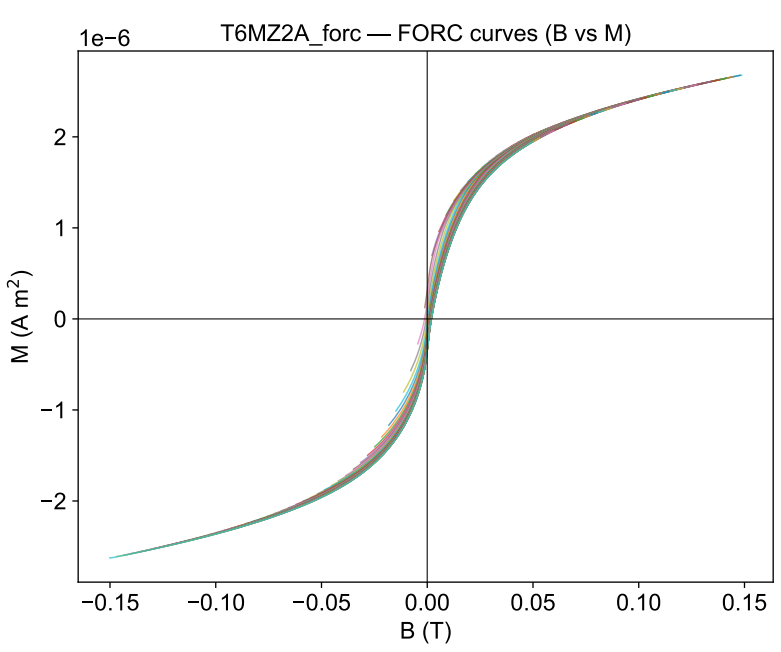

T6MZ2A_forc · FORC curves (B vs M).pdf

Fig. S7. Same as in S4 but for T4MZ2C (upper left) to T6MZ2A (lower left).

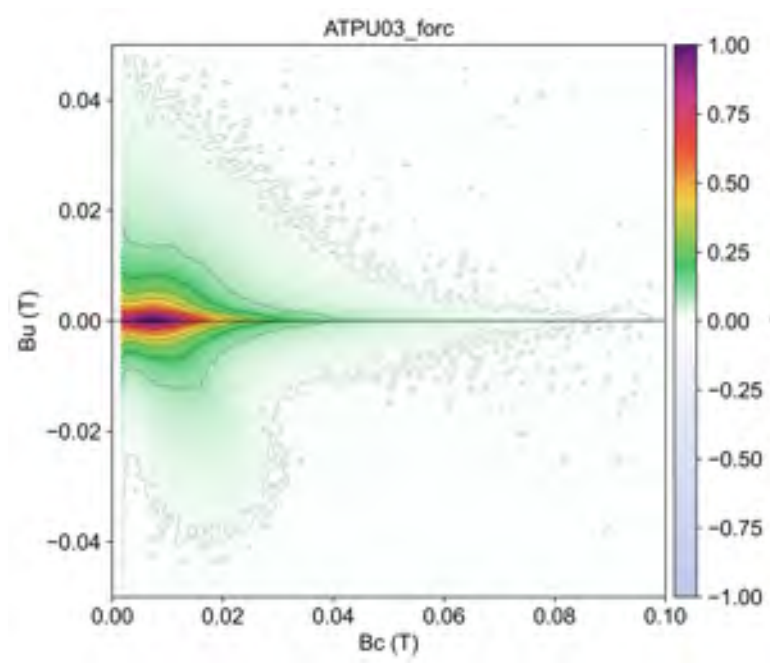

ATPU03_forc_FORC.pdf

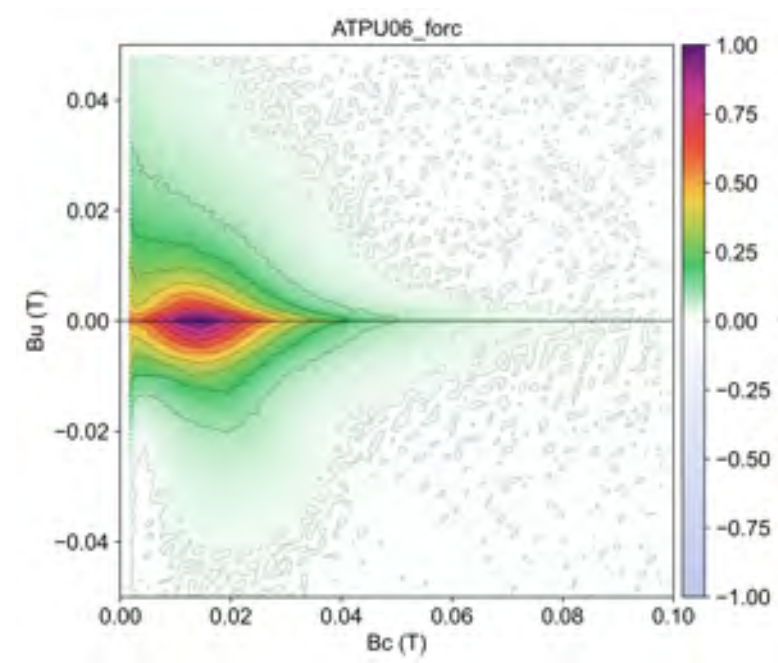

ATPU06_forc_FORC.pdf

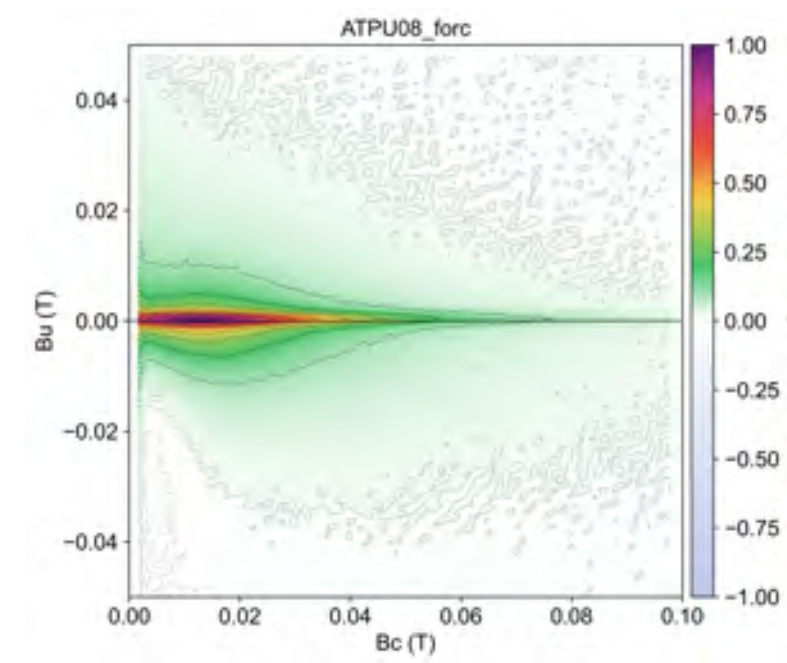

ATPU08_forc_FORC.pdf

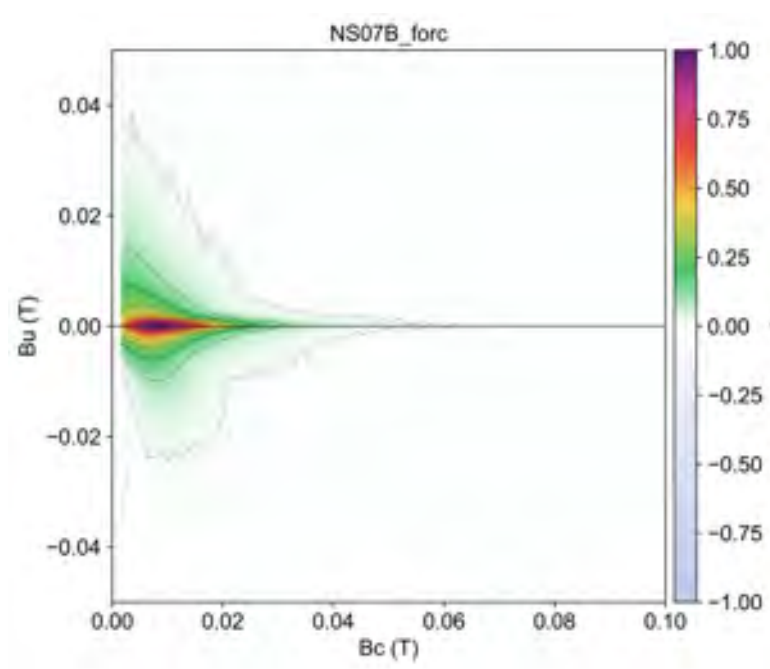

NS07B_forc_FORC.pdf

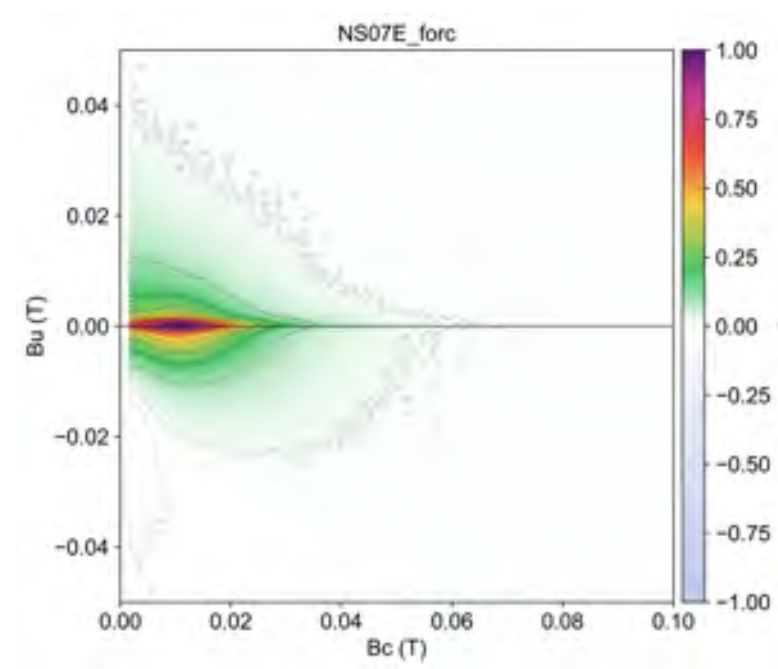

NS07E_forc_FORC.pdf

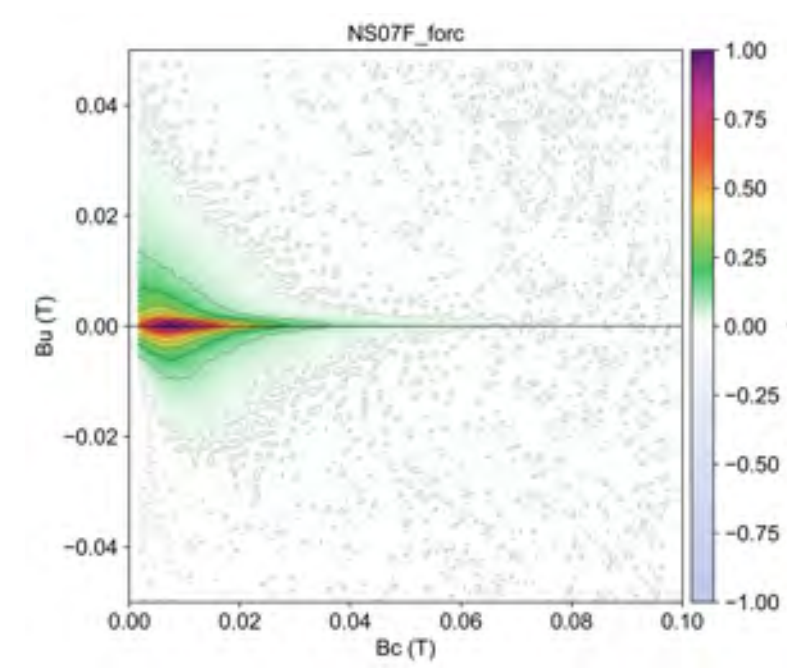

NS07F_forc_FORC.pdf

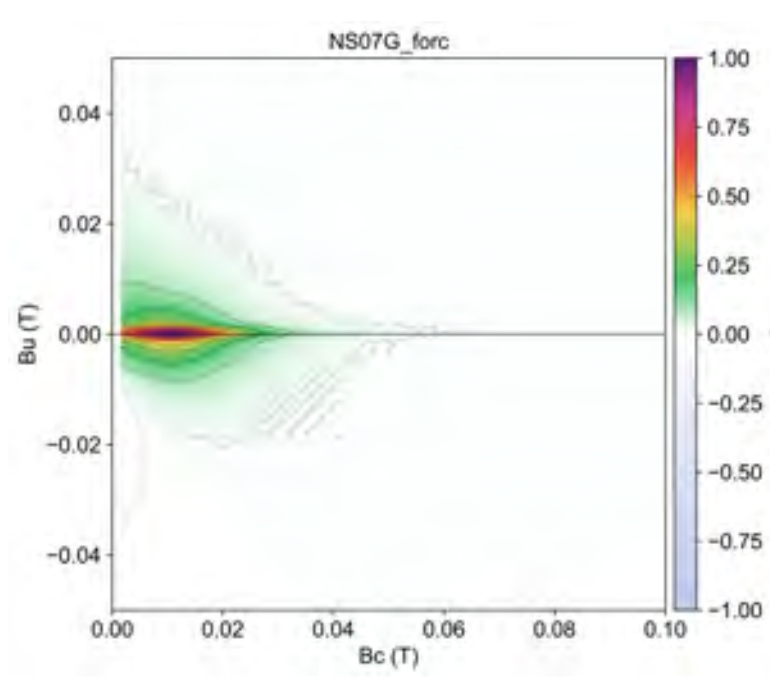

NS07G_forc_FORC.pdf

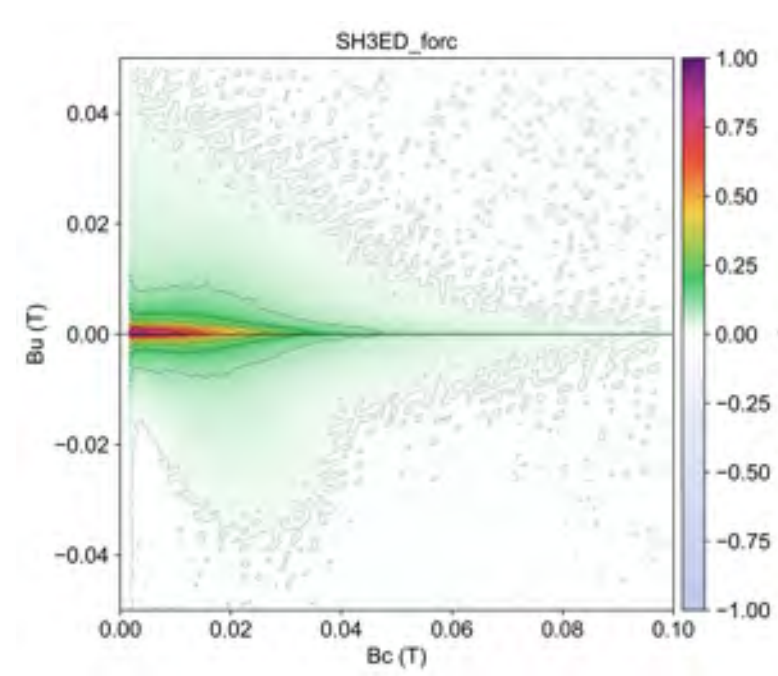

SH3ED_forc_FORC.pdf

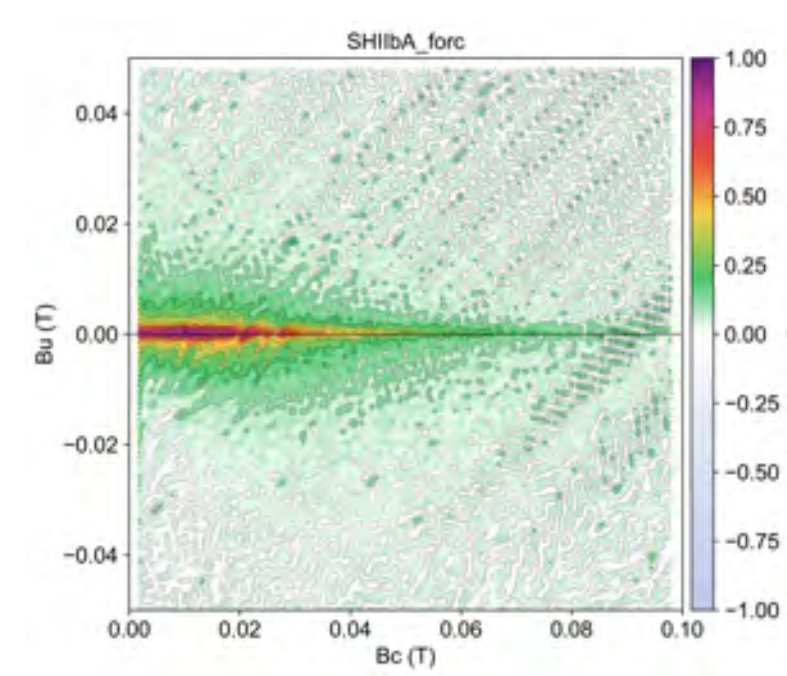

SHIIbA_forc_FORC.pdf

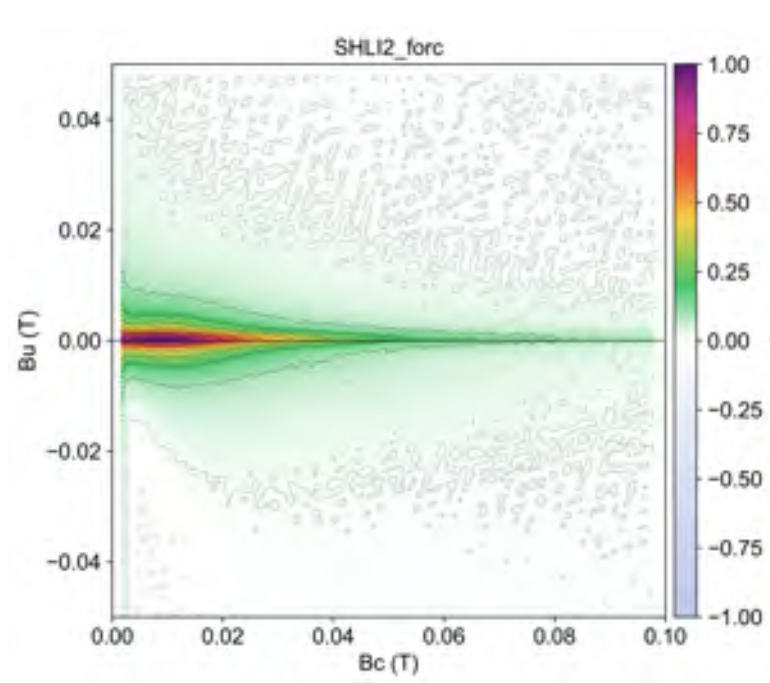

SHLI2_forc_FORC.pdf

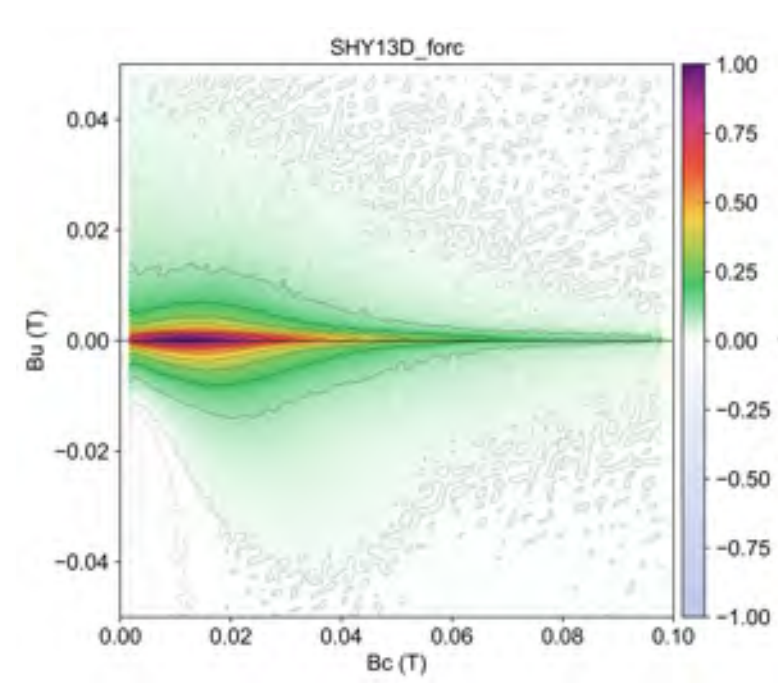

SHY13D_forc_FORC.pdf

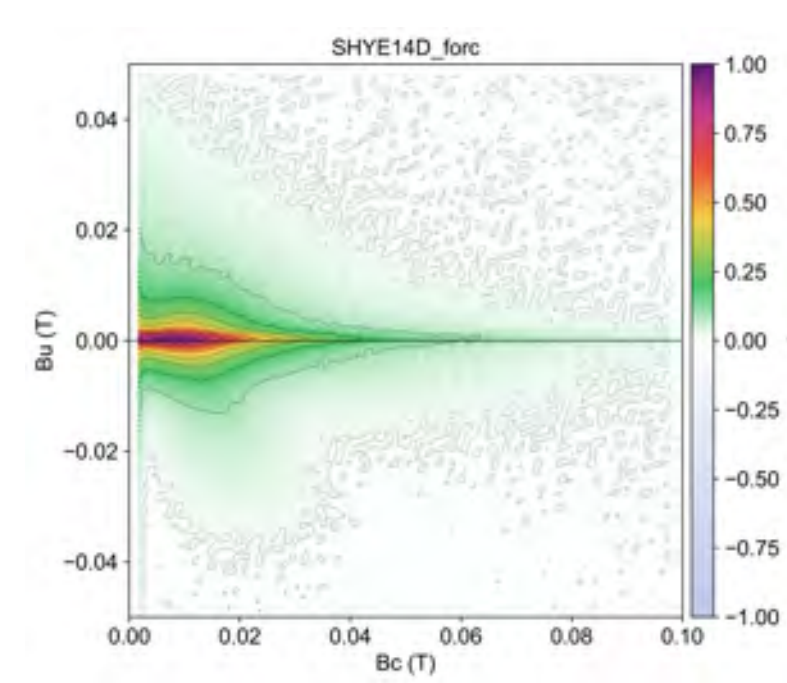

SHYE14D_forc_FORC.pdf

Fig. S8. FORC diagrams for the curves shown in Fig. S4. The FORCs were generated with the FORCme code (7) using a smoothing factor of 0.5.

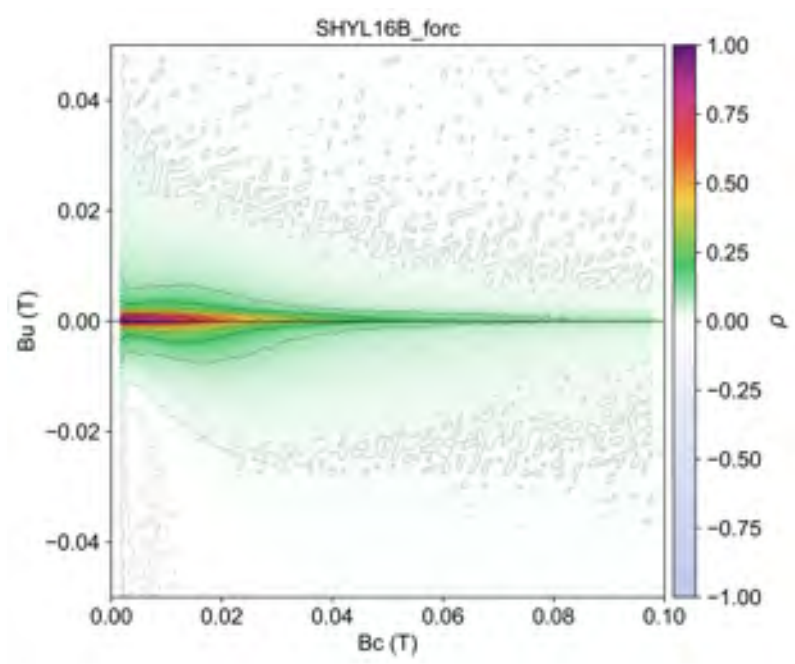

SHYL16B_forc_FORC.pdf

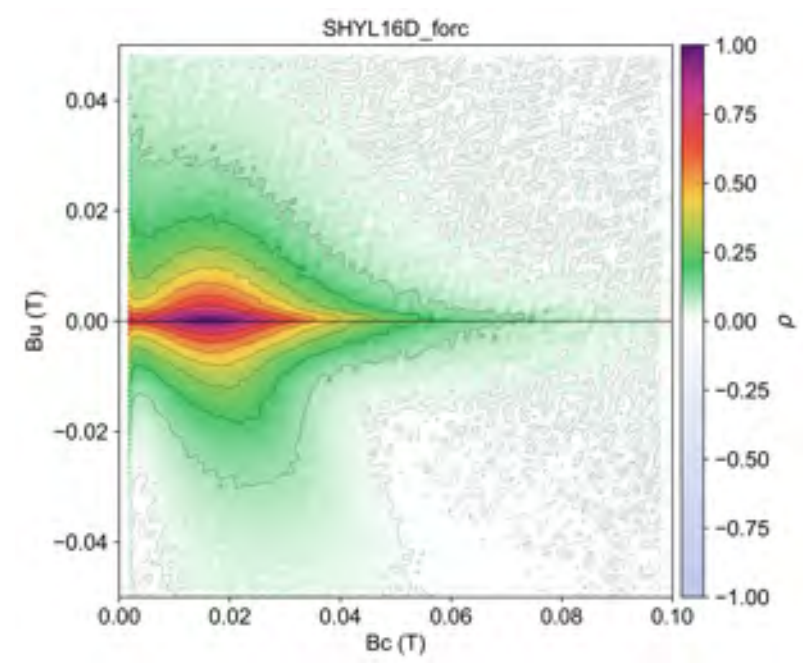

SHYL16D_forc_FORC.pdf

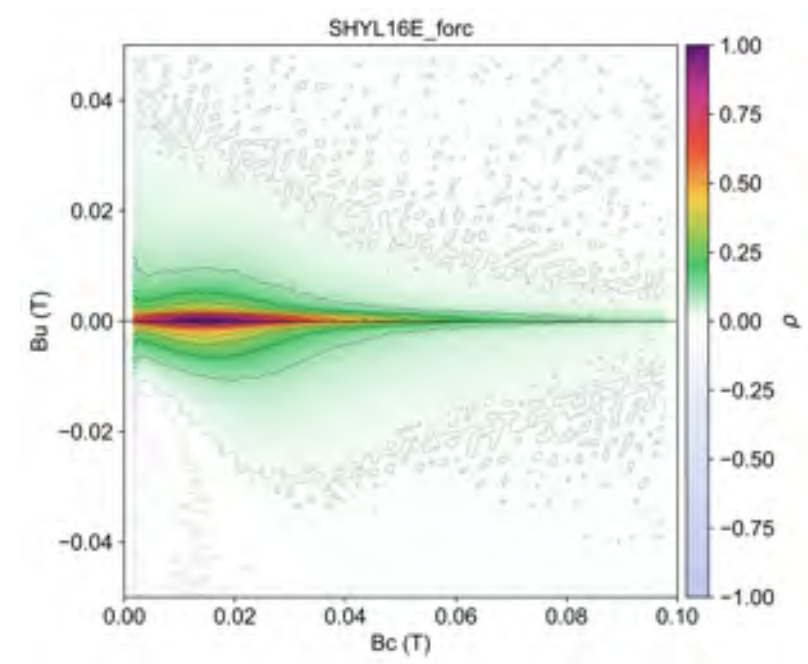

SHYL16E_forc_FORC.pdf

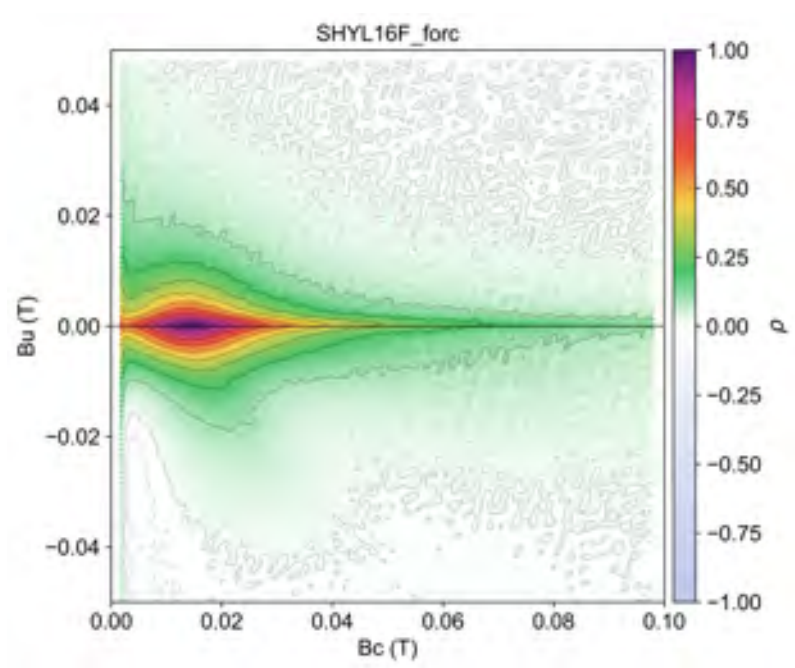

SHYL16F_forc_FORC.pdf

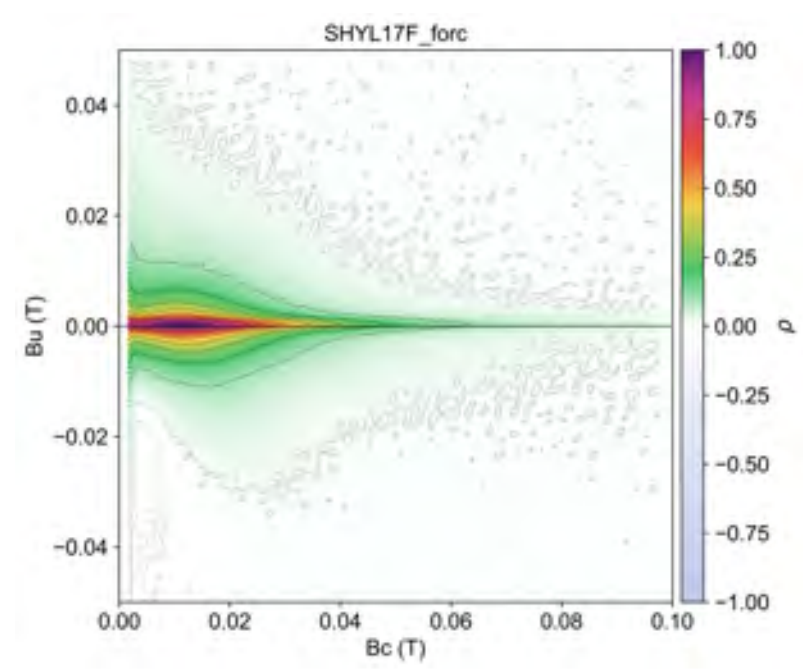

SHYL17F_forc_FORC.pdf

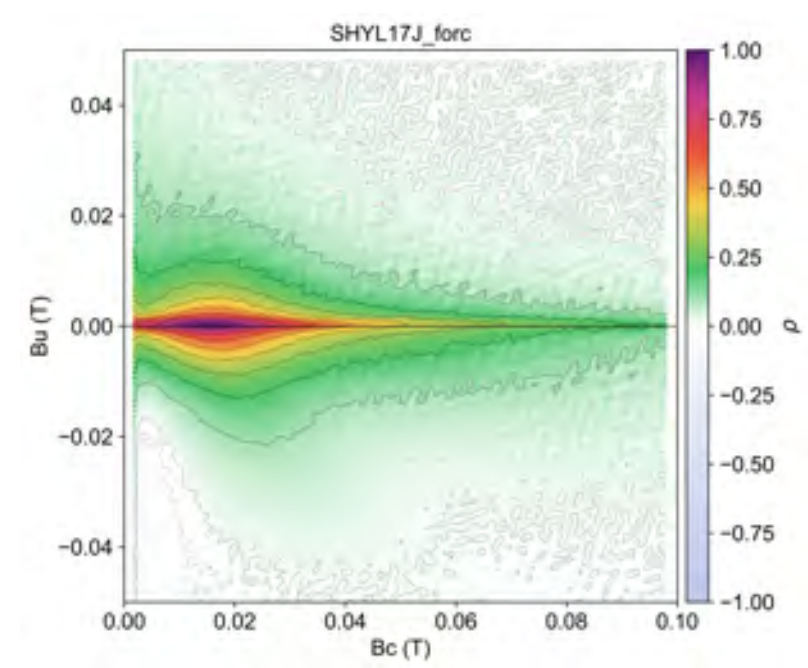

SHYL17J_forc_FORC.pdf

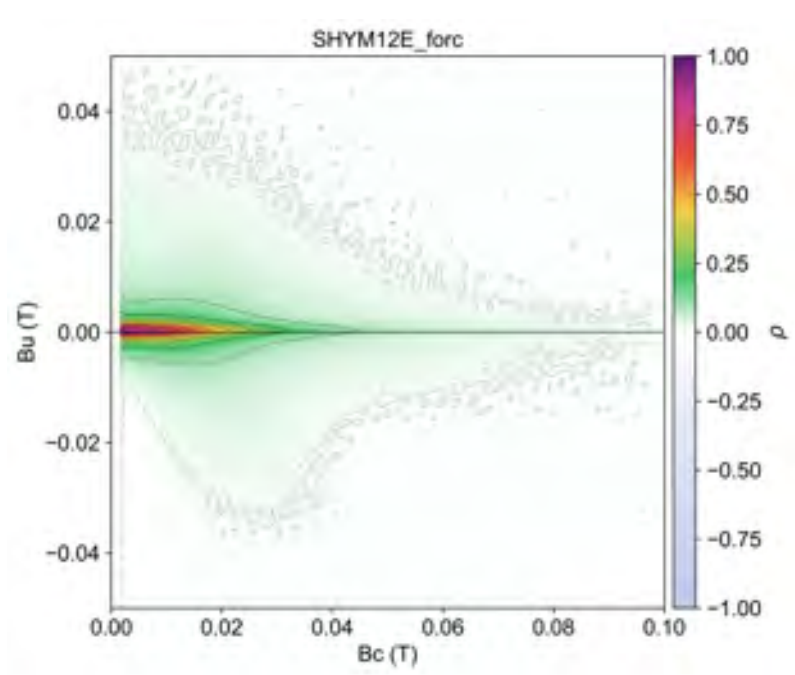

SHYM12E_forc_FORC.pdf

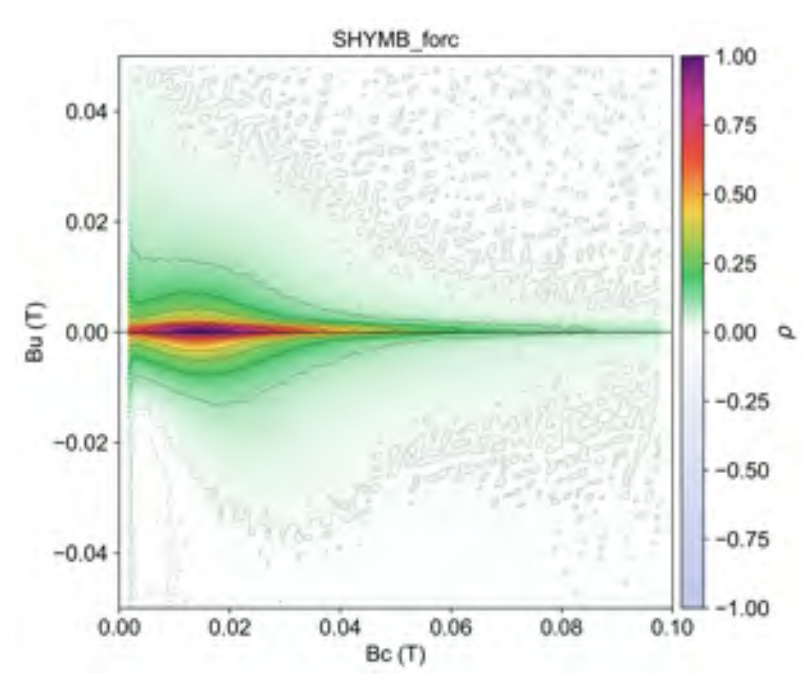

SHYMB_forc_FORC.pdf

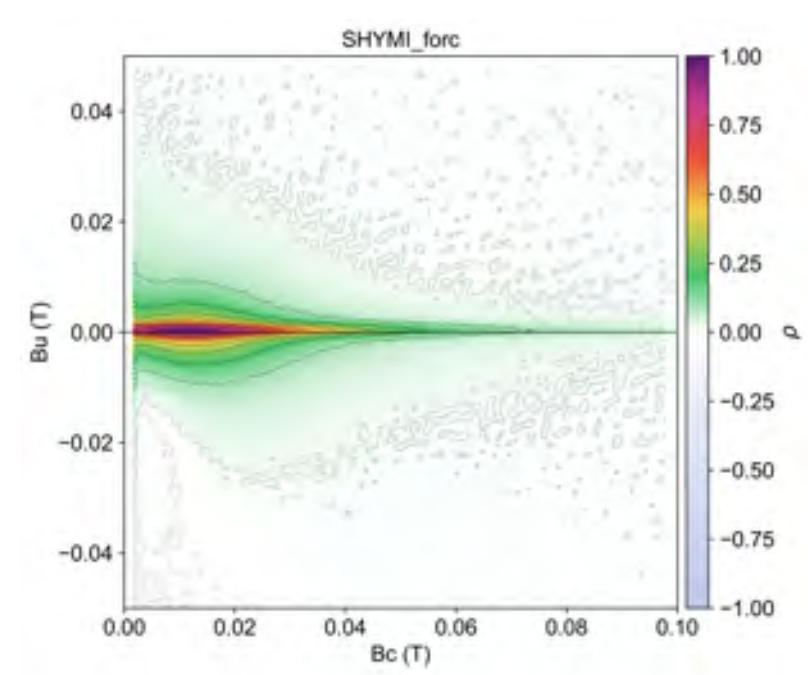

SHYMI_forc_FORC.pdf

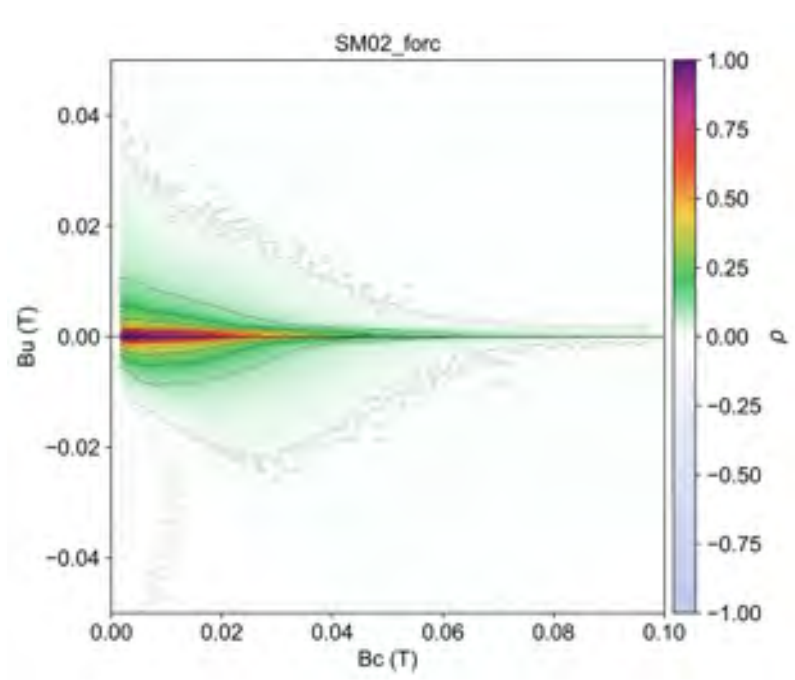

SM02_forc_FORC.pdf

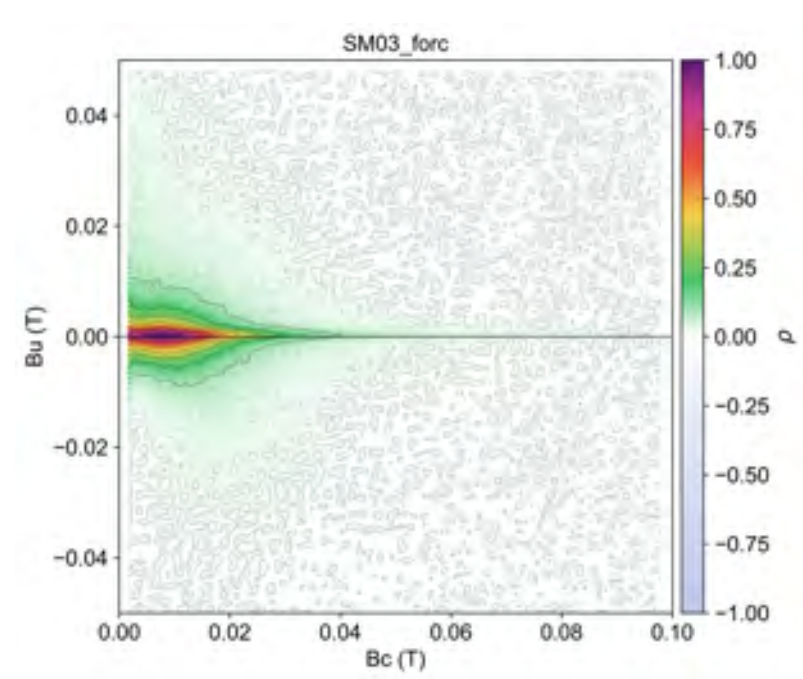

SM03_forc_FORC.pdf

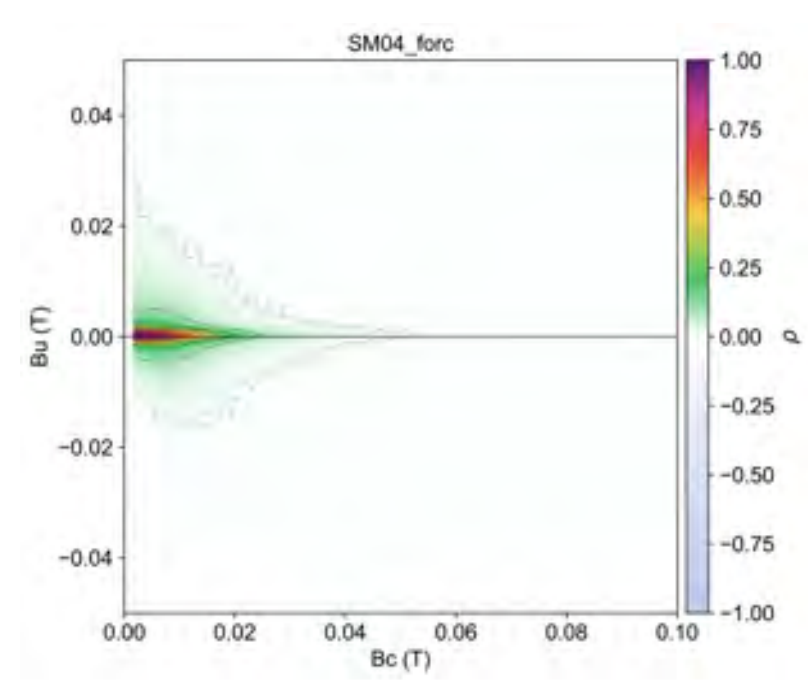

SM04_forc_FORC.pdf

Fig. S9. Same as in Fig. S8 but for the data shown in S5.

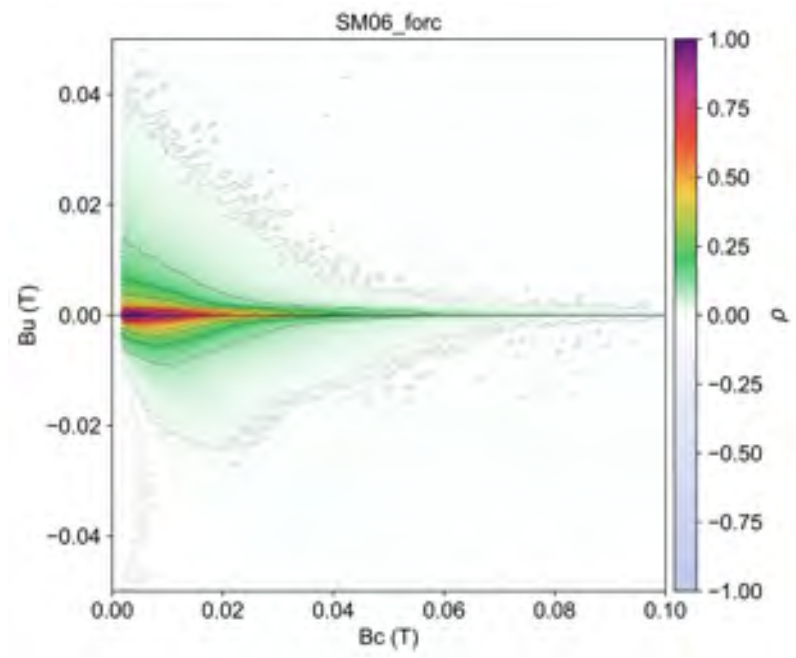

SM06_forc_FORC.pdf

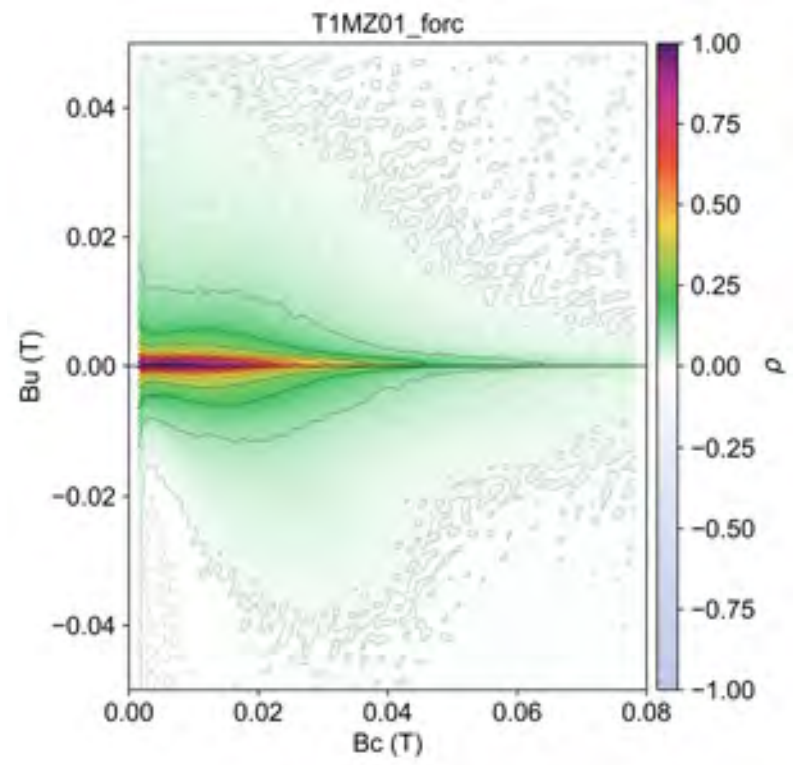

T1MZ01_forc_FORC.pdf

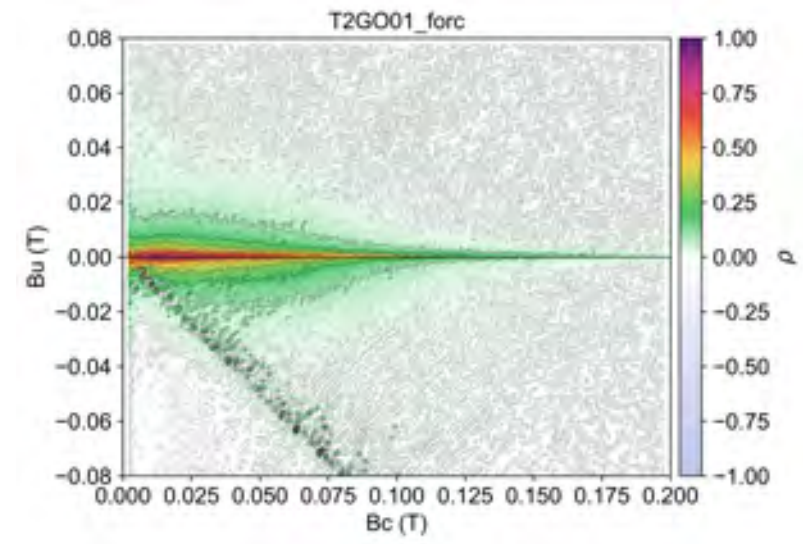

T2GO01_forc_FORC.pdf

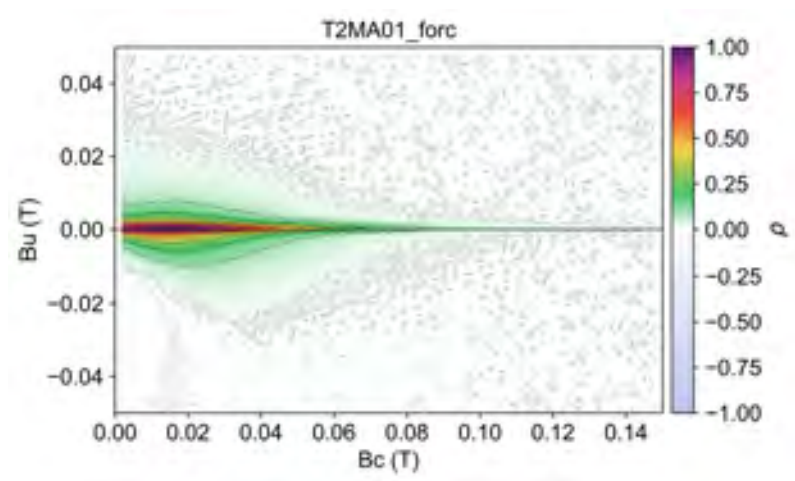

T2MA01_forc_FORC.pdf

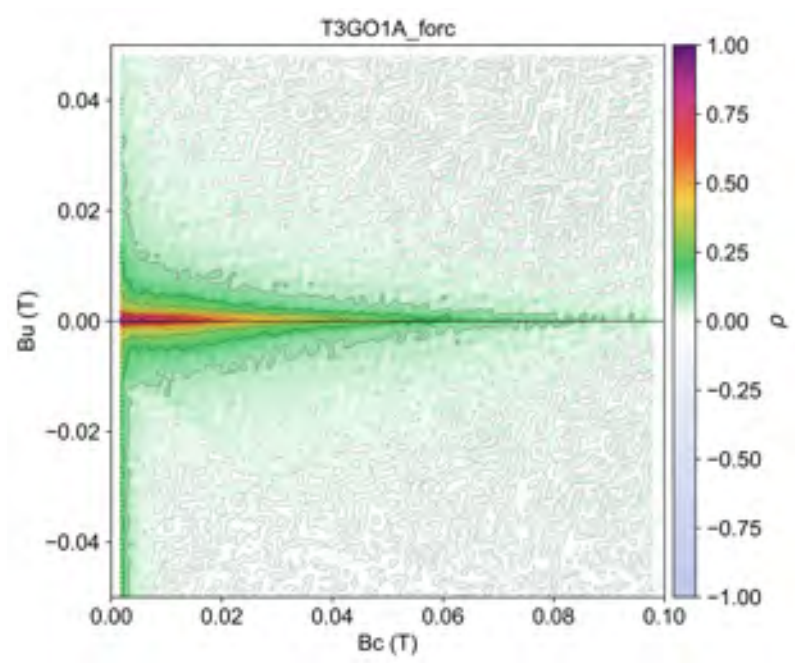

T3GO1A_forc_FORC.pdf

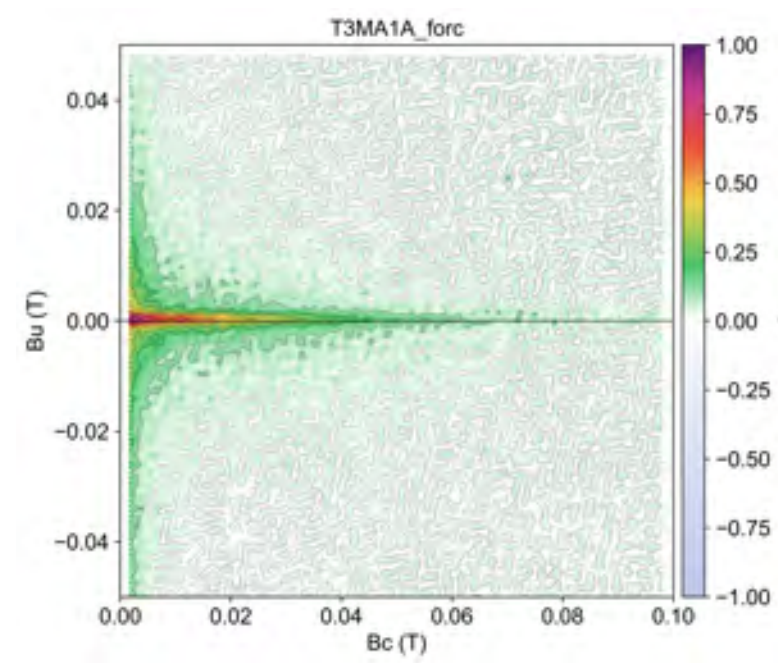

T3MA1A_forc_FORC.pdf

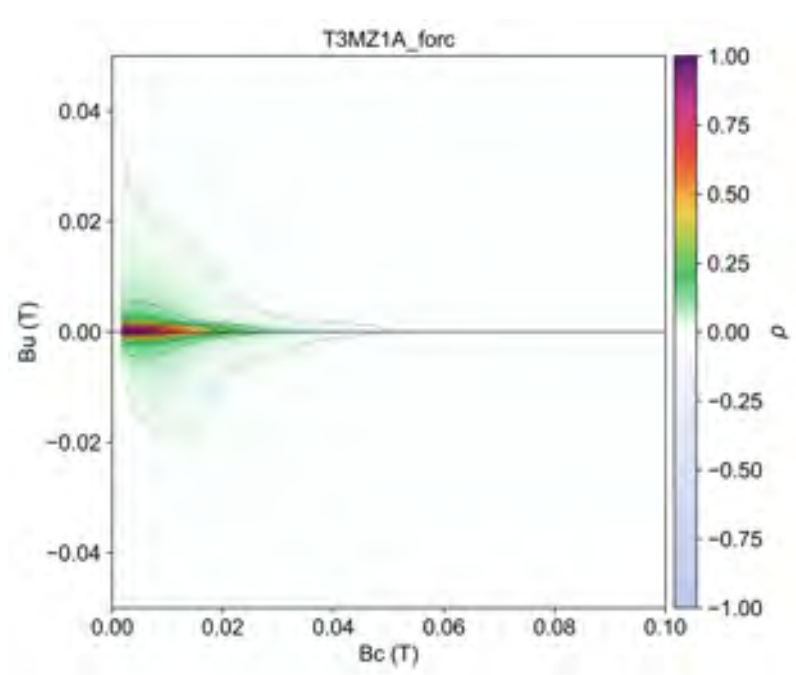

T3MZ1A_forc_FORC.pdf

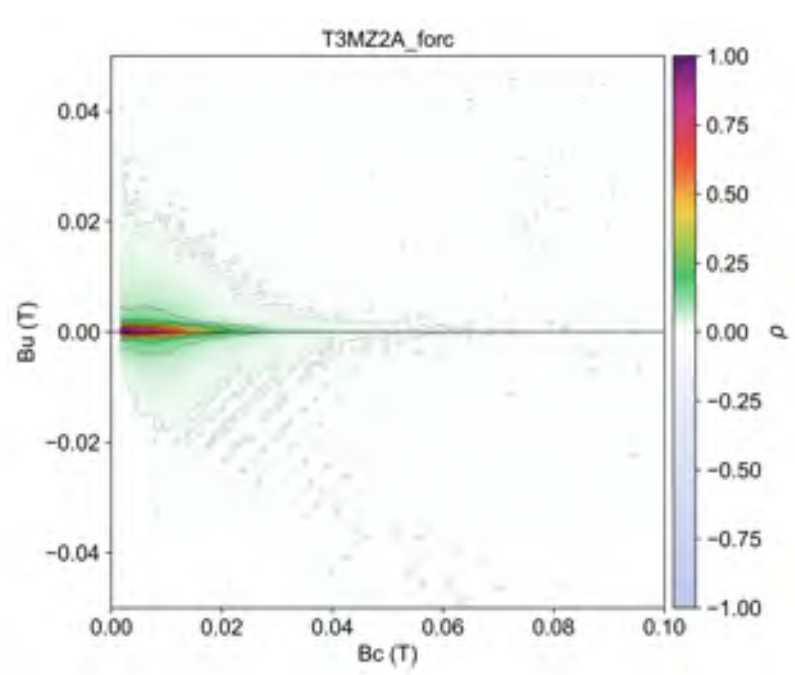

T3MZ2A_forc_FORC.pdf

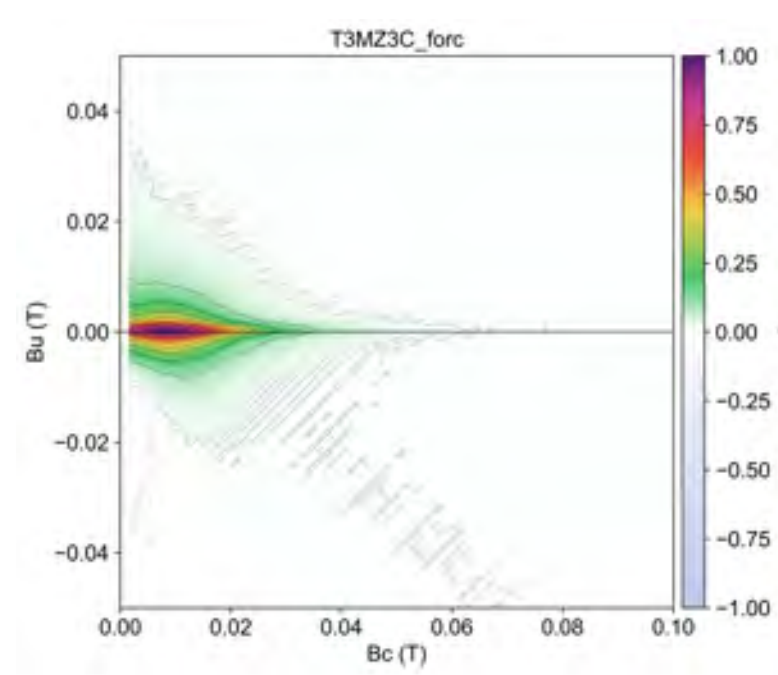

T3MZ3C_forc_FORC.pdf

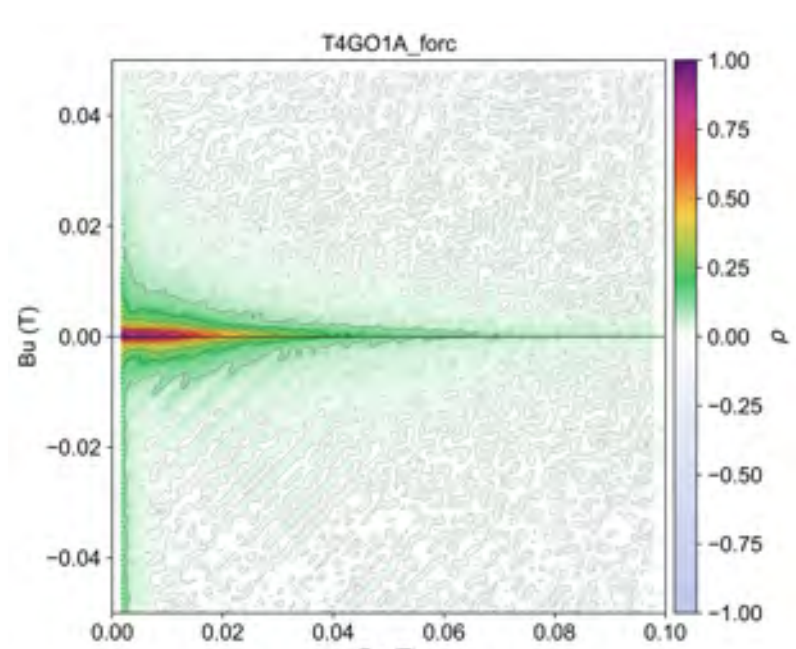

T4GO1A_forc_FORC.pdf

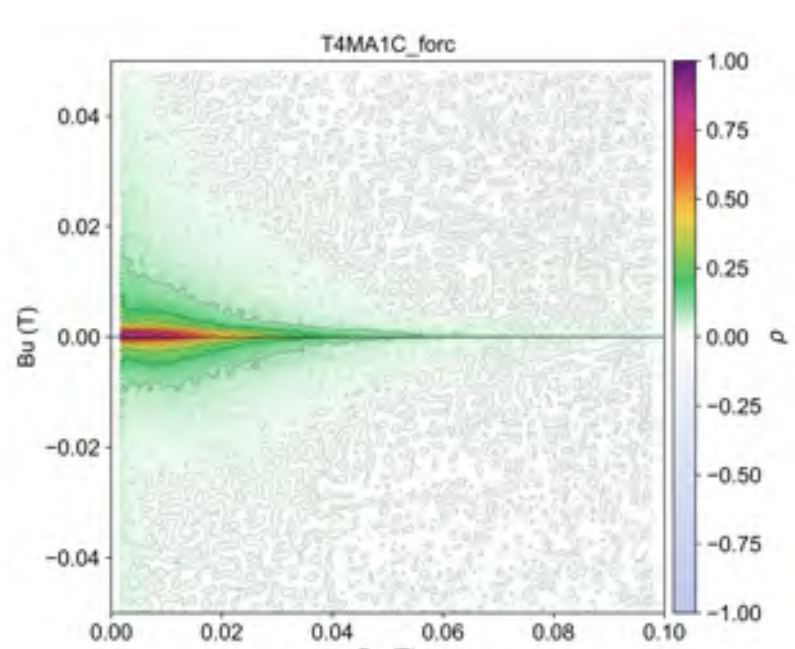

T4MA1C_forc_FORC.pdf

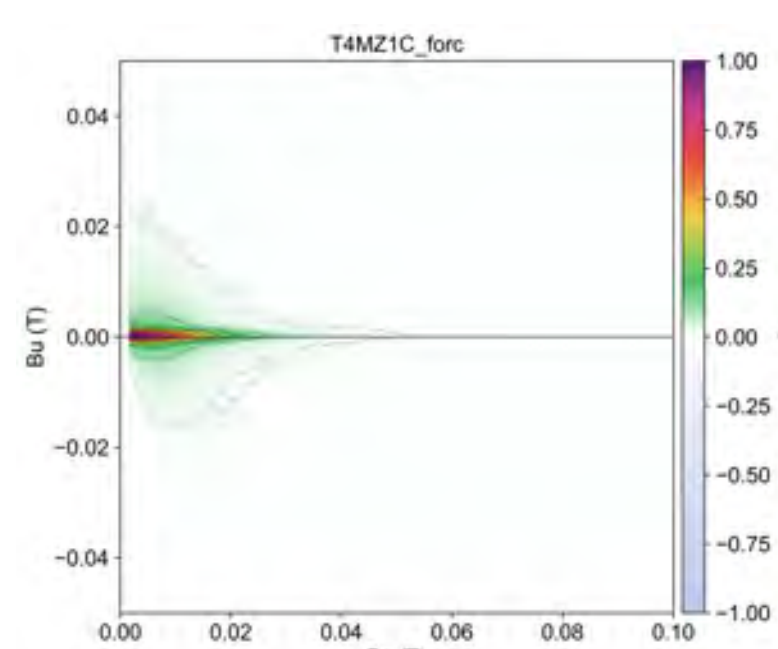

T4MZ1C_forc_FORC.pdf

Fig. S10. Same as in Fig.S8 but for the data shown in S6.

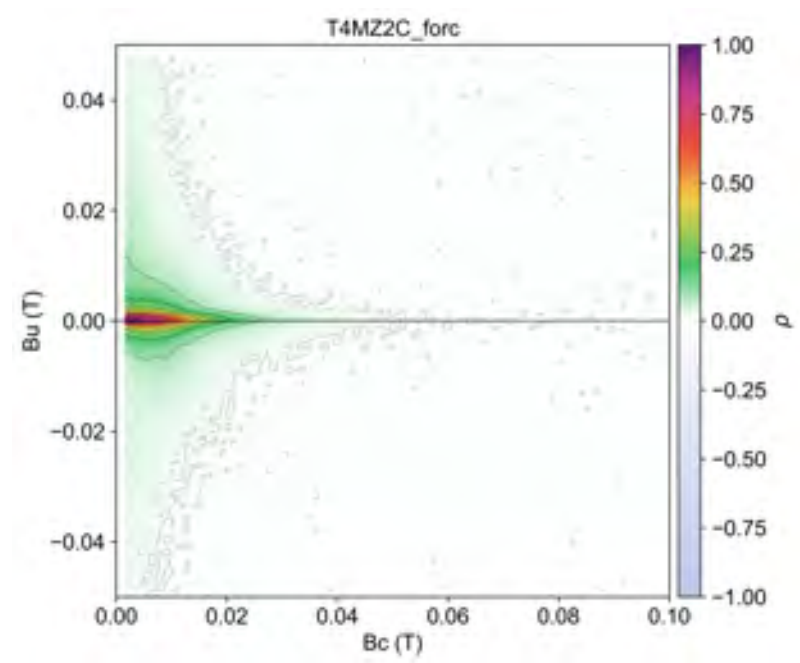

T4MZ2C_forc_FORC.pdf

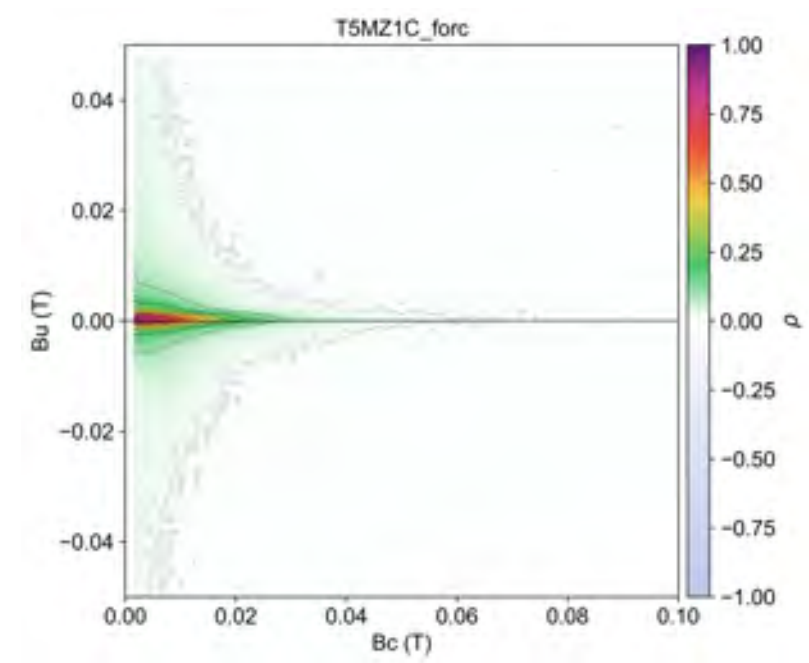

T5MZ1C_forc_FORC.pdf

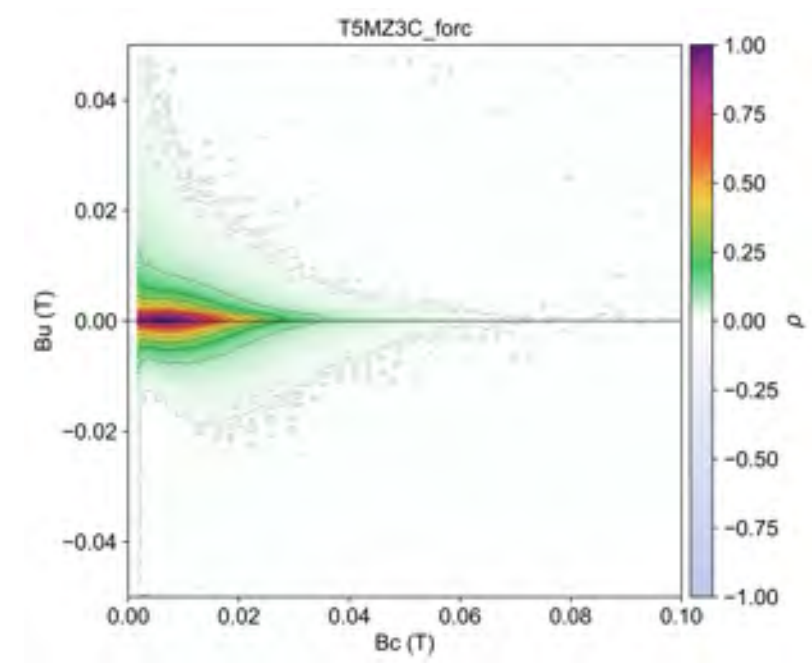

T5MZ3C_forc_FORC.pdf

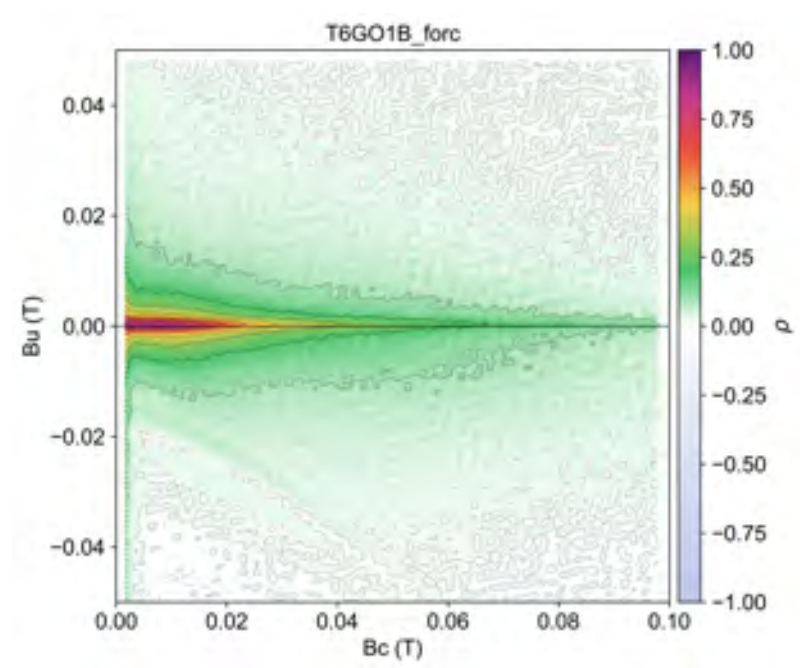

T6GO1B_forc_FORC.pdf

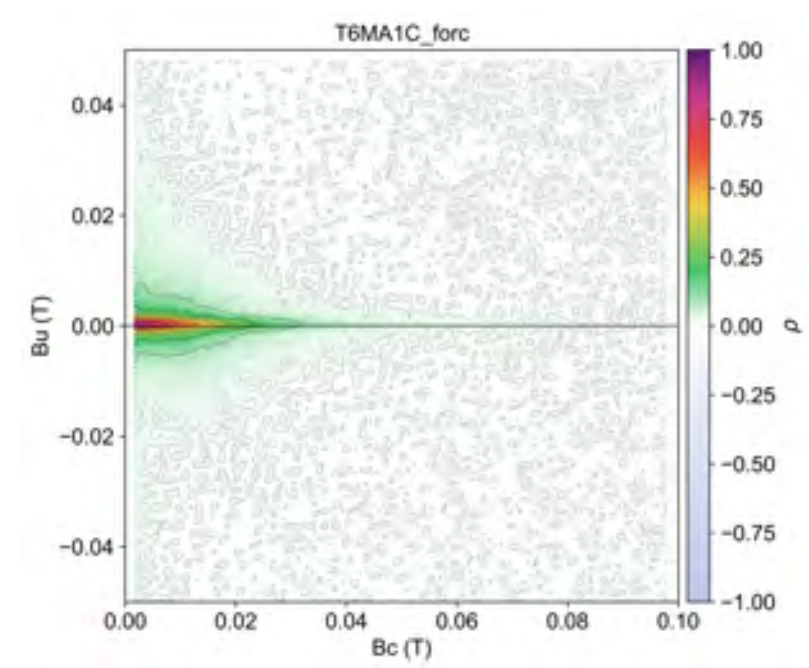

T6MA1C_forc_FORC.pdf

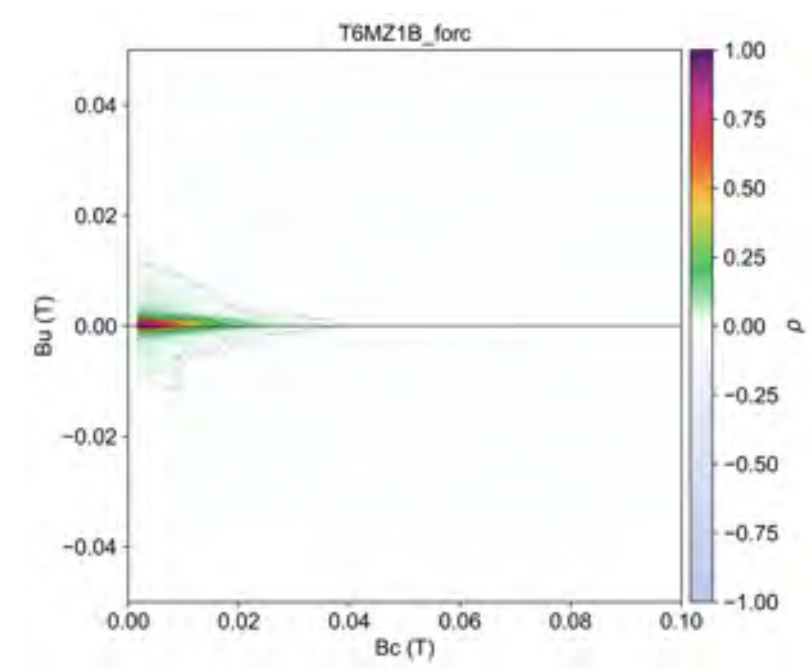

T6MZ1B_forc_FORC.pdf

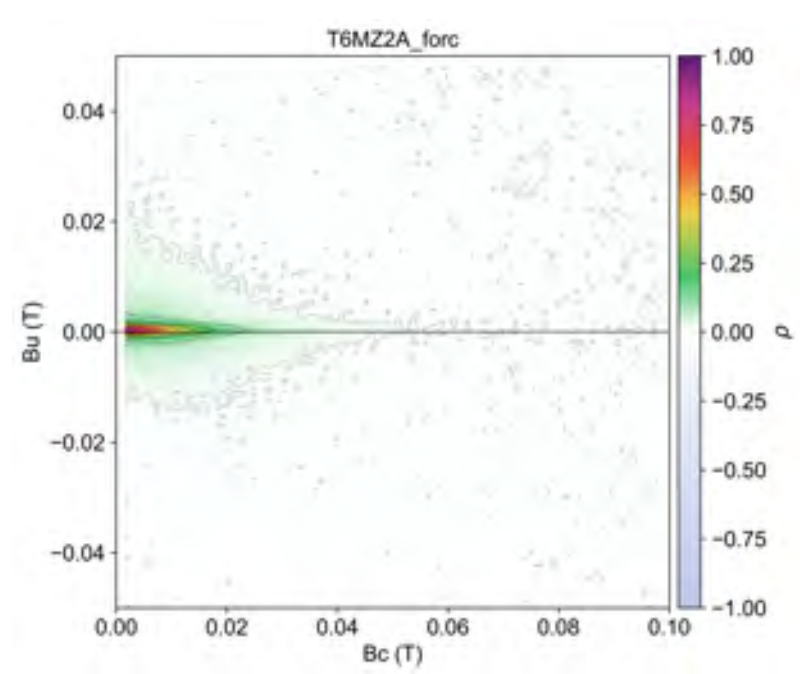

T6MZ2A_forc_FORC.pdf

Fig. S11. Same as in Fig.S8 but for the data shown in S7.

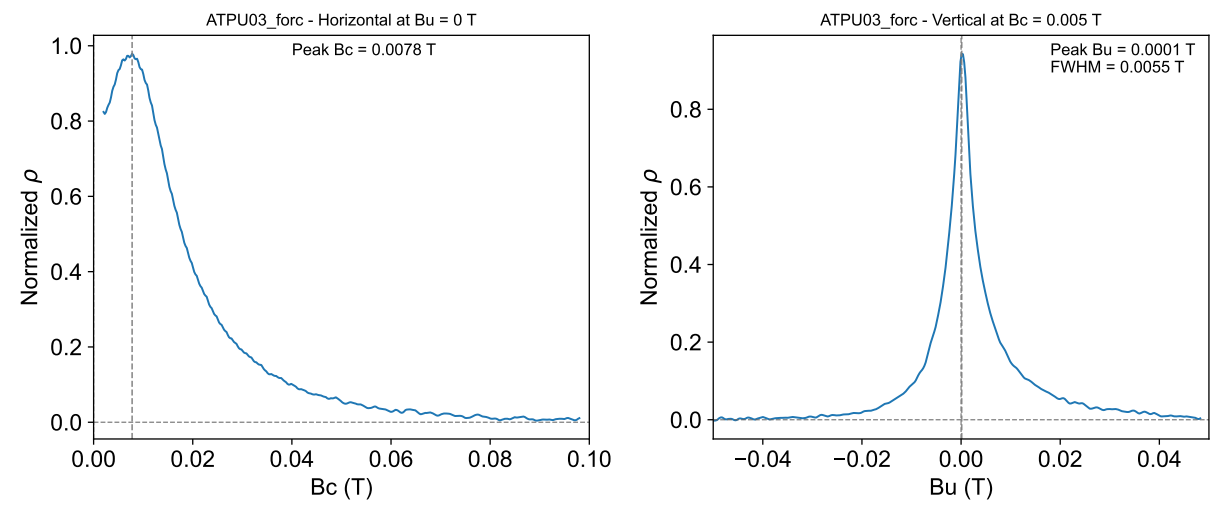

ATPU03_forc_custom_profiles.pdf

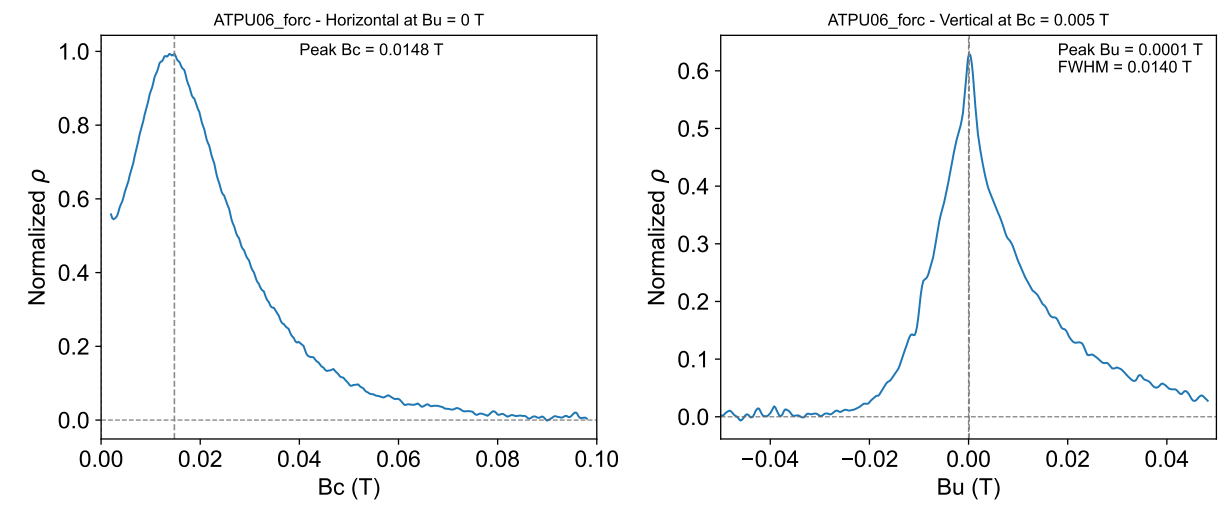

ATPU06_forc_custom_profiles.pdf

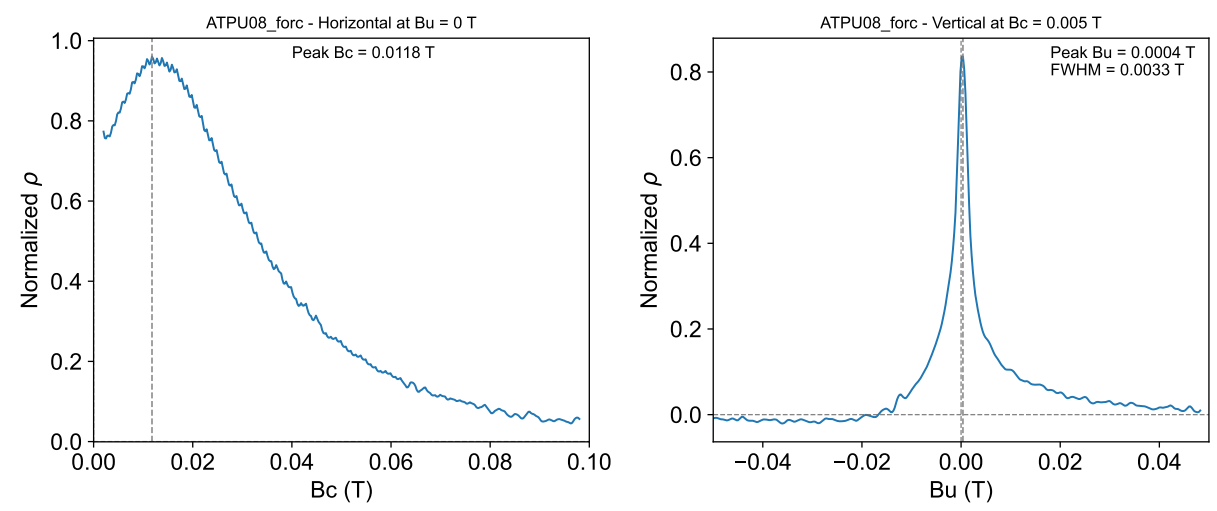

ATPU08_forc_custom_profiles.pdf

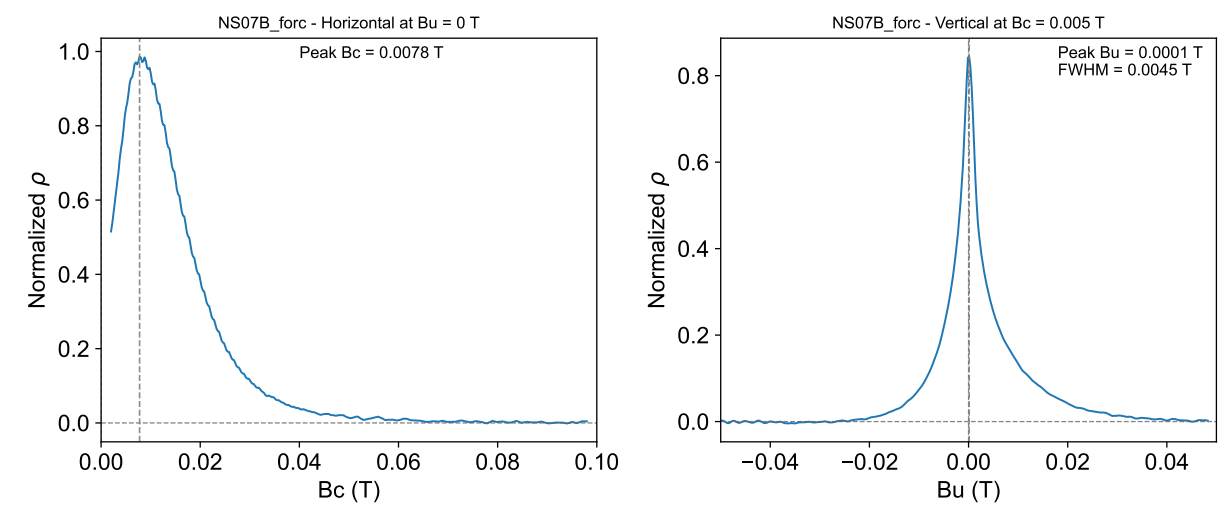

NS07B_forc_custom_profiles.pdf

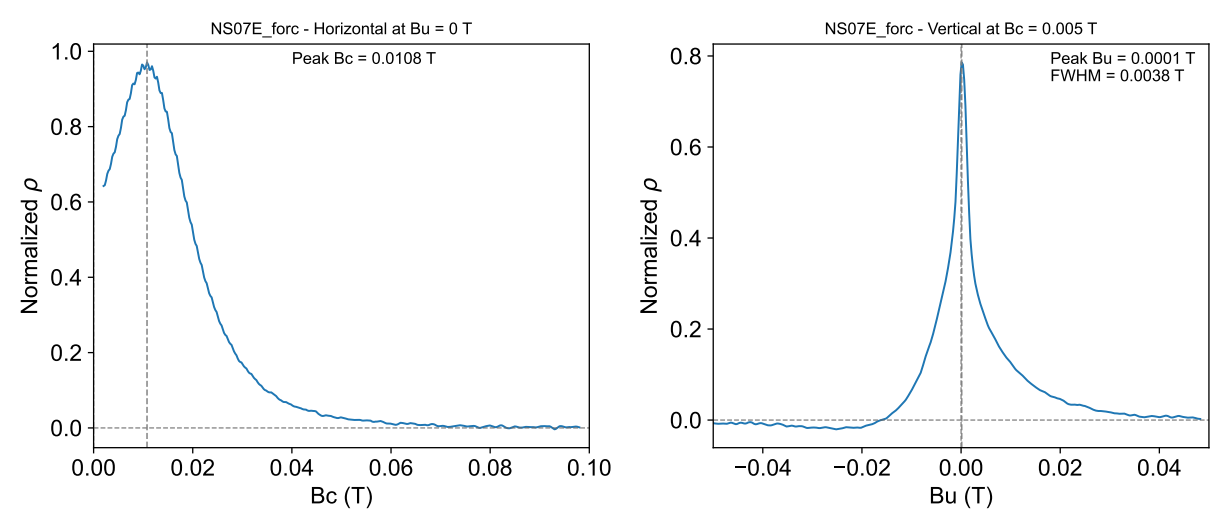

NS07E_forc_custom_profiles.pdf

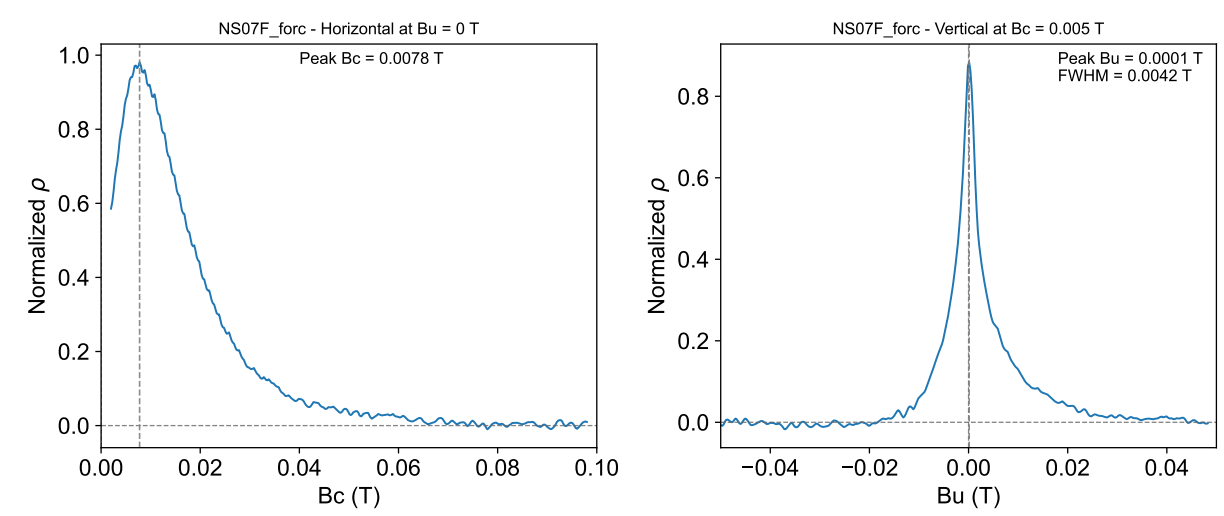

NS07F_forc_custom_profiles.pdf

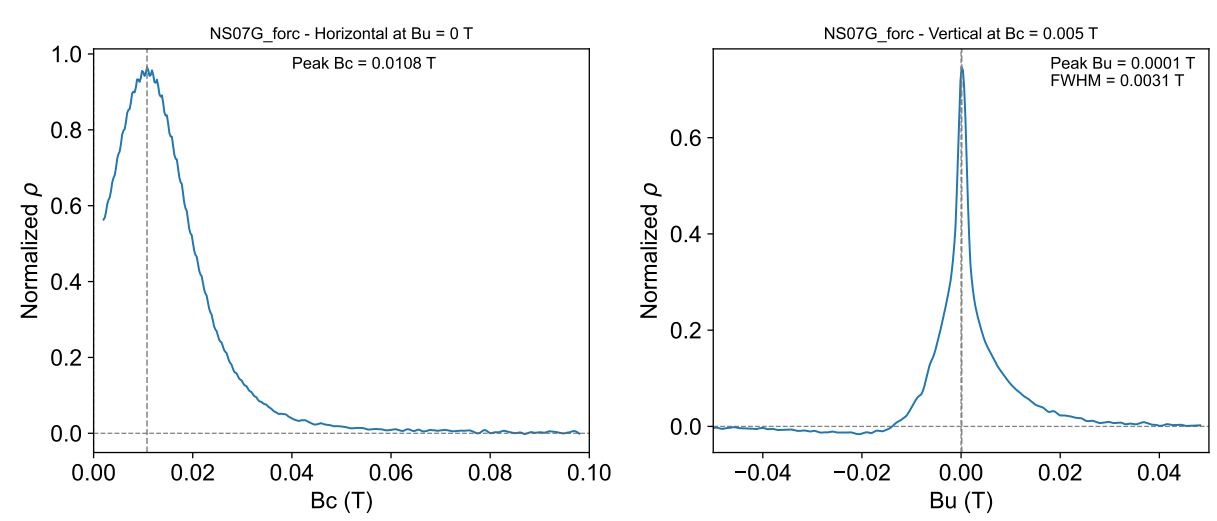

NS07G_forc_custom_profiles.pdf

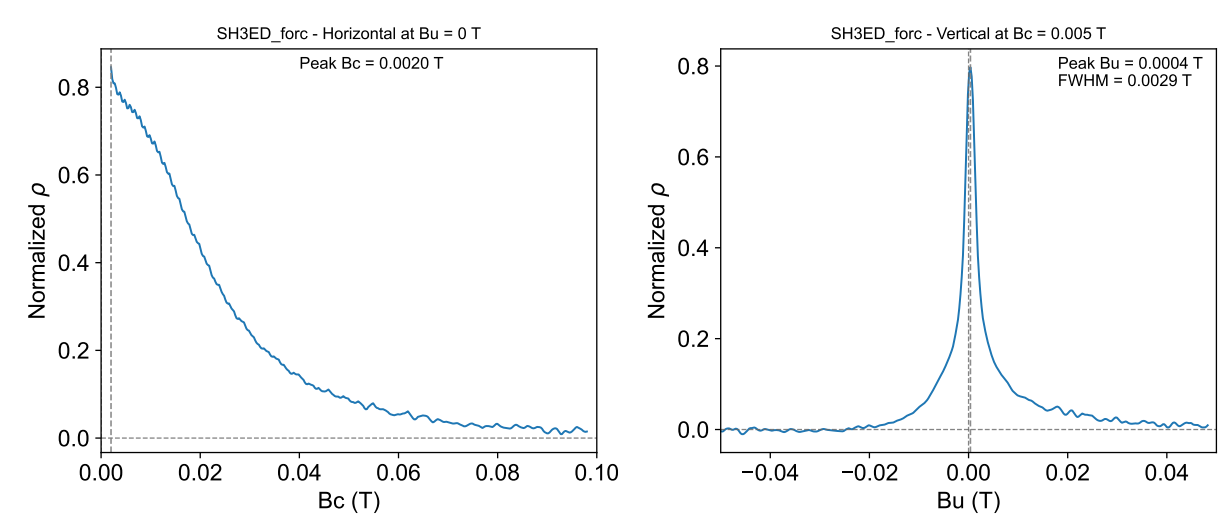

SH3ED_forc_custom_profiles.pdf

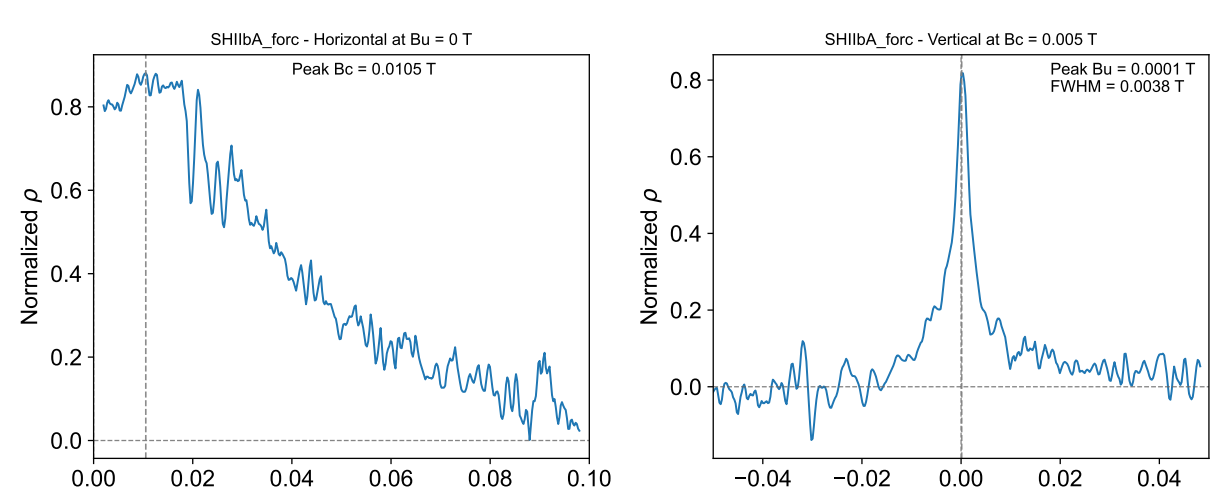

SHIIbA_forc_custom_profiles.pdf

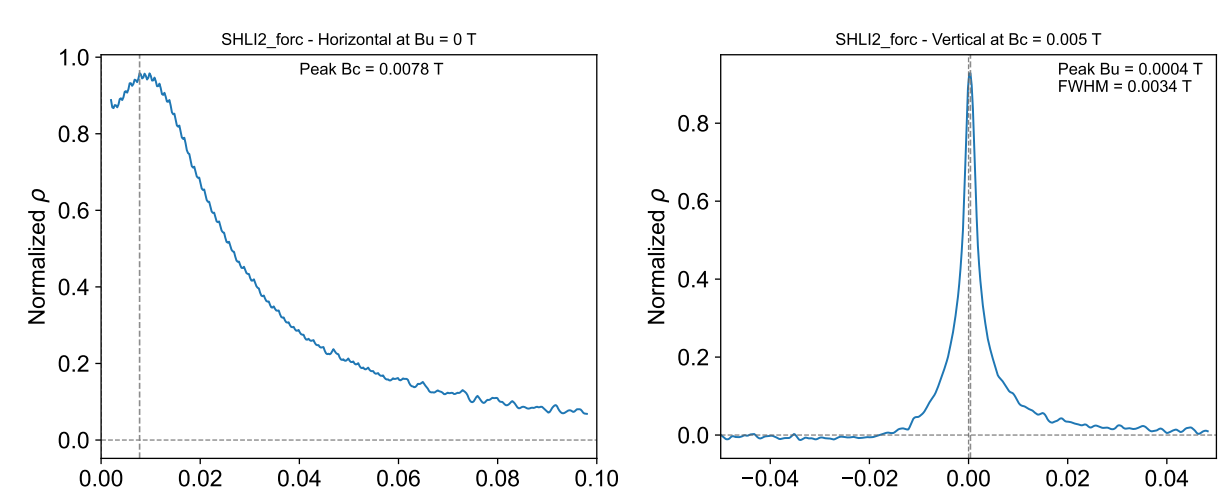

SHLI2_forc_custom_profiles.pdf

Fig. S12. FORC density profile plots for samples ATPU03 to SHLI2. Each sample has two plots: FORC density (ρ) against coercivity (Bc) in tesla and ρ against Bu. These are the FORC densities (ρ represented by colors) in the FORC diagrams near the peaks in Bc and Bu. These plots were generated with the FORCme code (7).

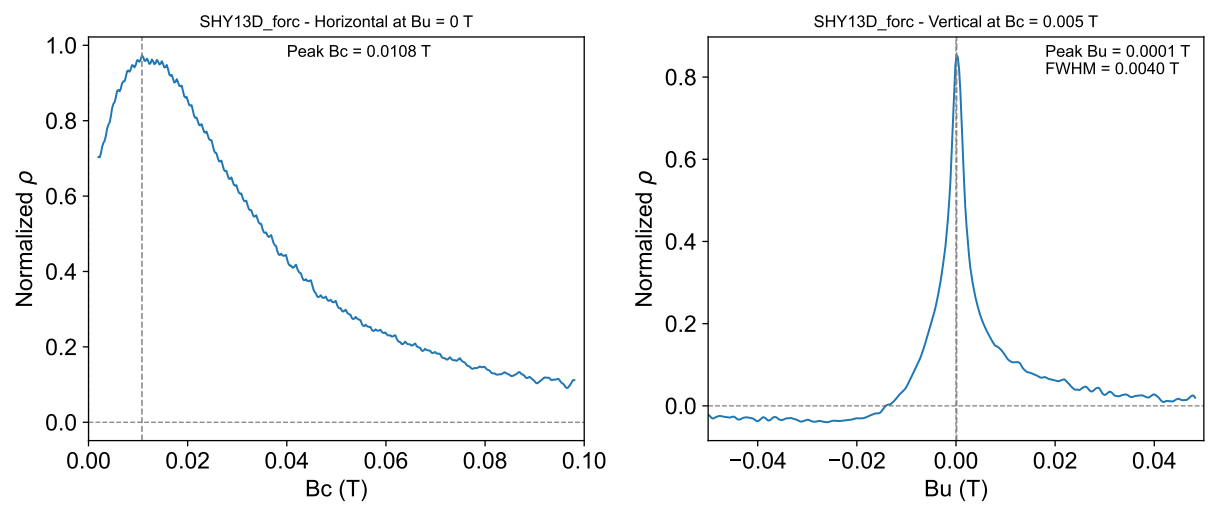

SHY13D_forc_custom_profiles.pdf

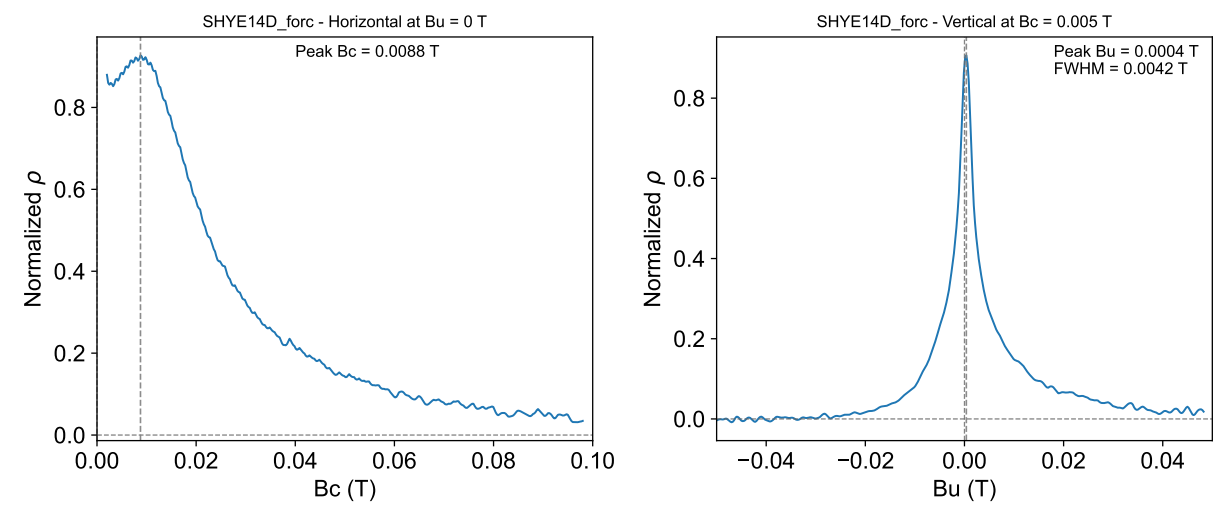

SHYE14D_forc_custom_profiles.pdf

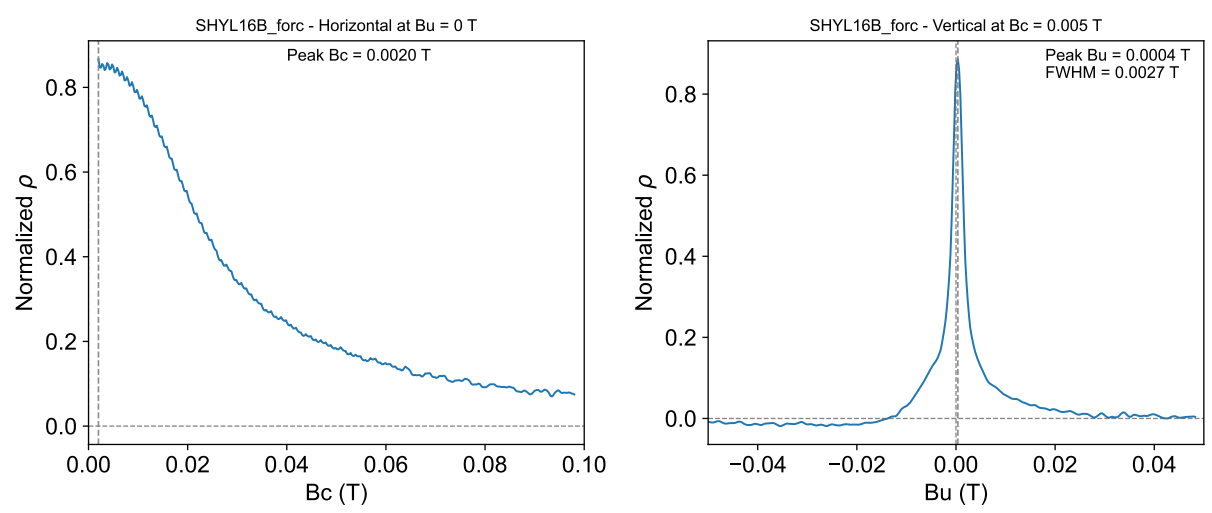

SHYL16B_forc_custom_profiles.pdf

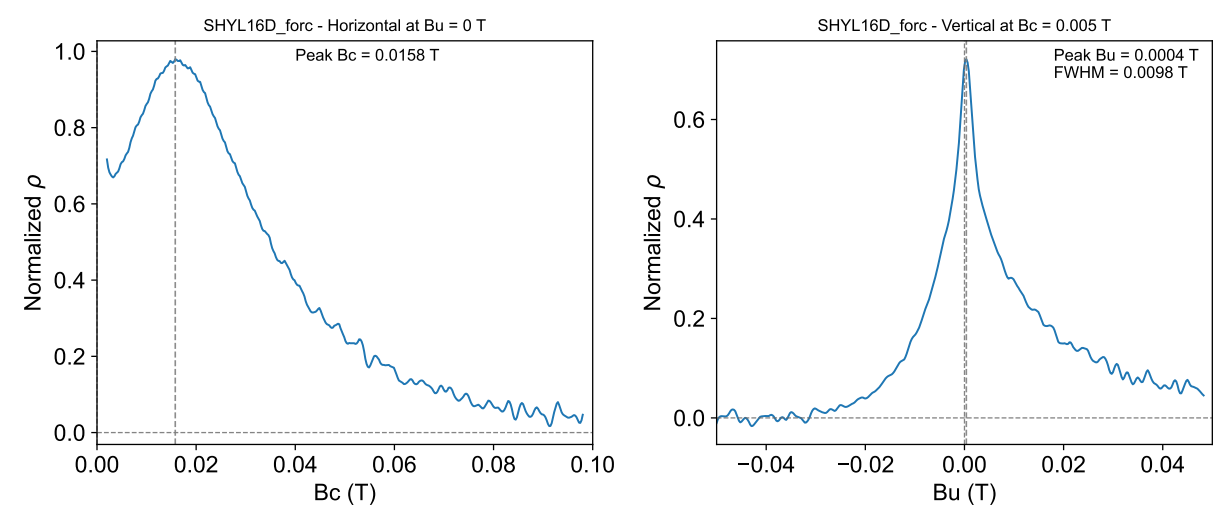

SHYL16D_forc_custom_profiles.pdf

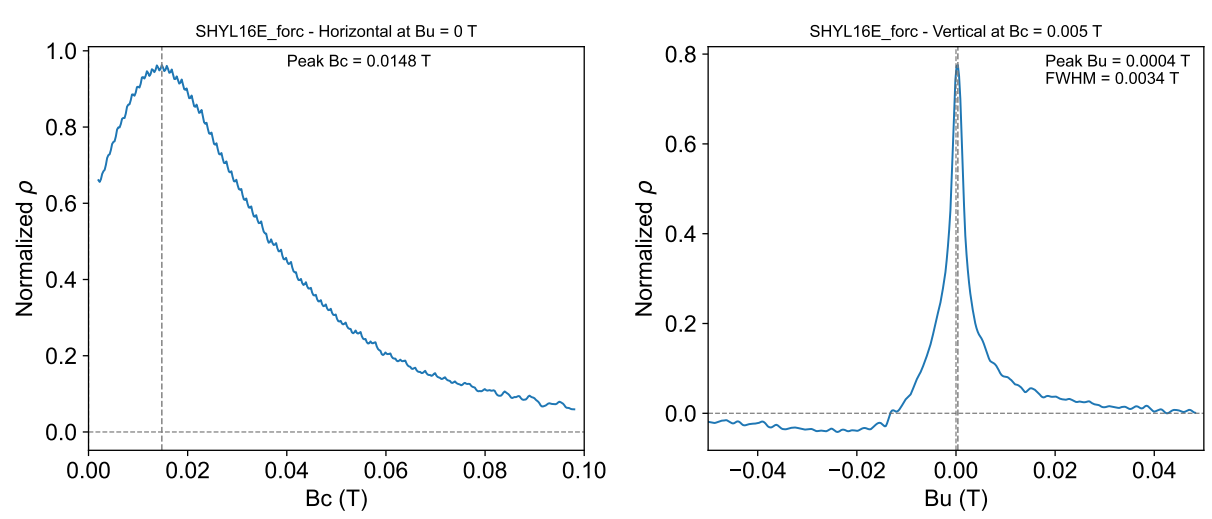

SHYL16E_forc_custom_profiles.pdf

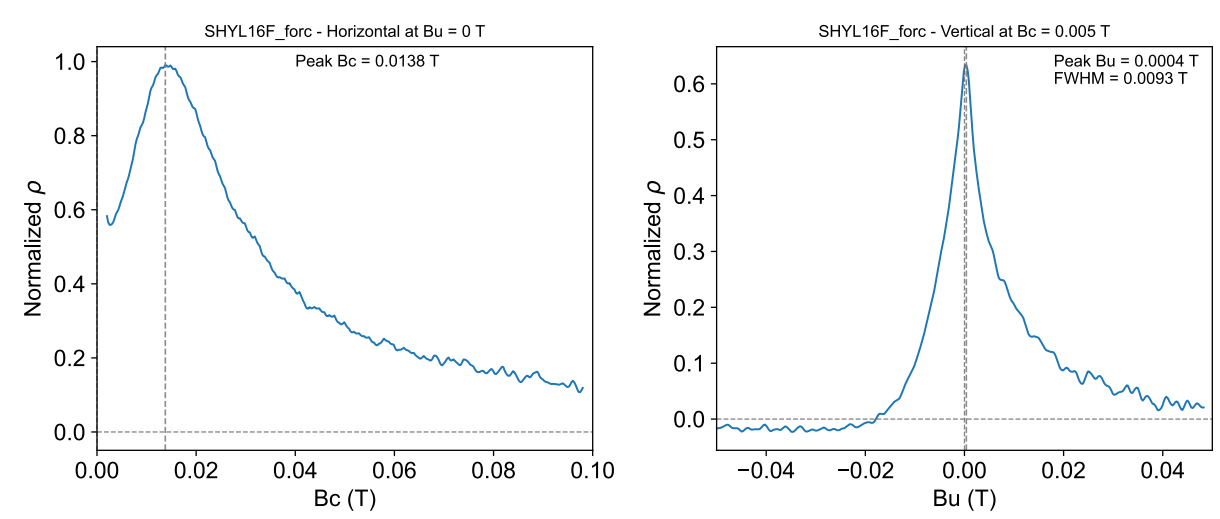

SHYL16F_forc_custom_profiles.pdf

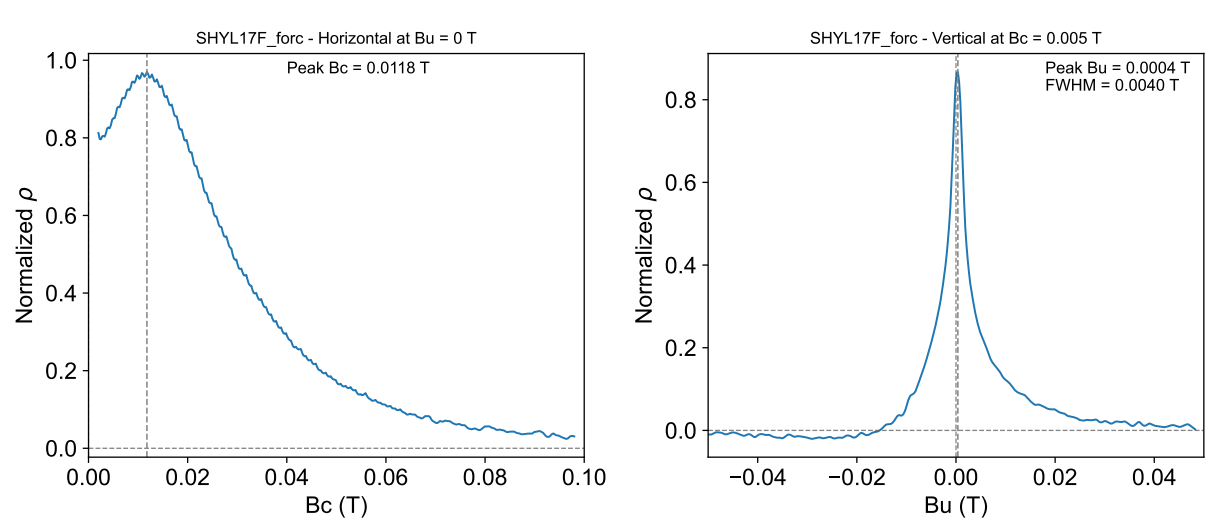

SHYL17F_forc_custom_profiles.pdf

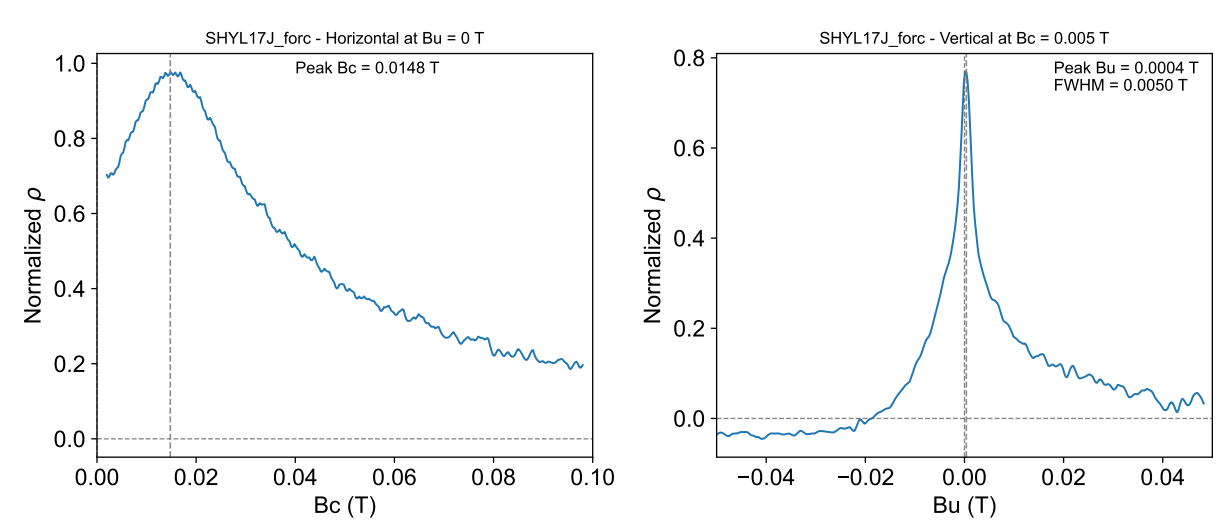

SHYL17J_forc_custom_profiles.pdf

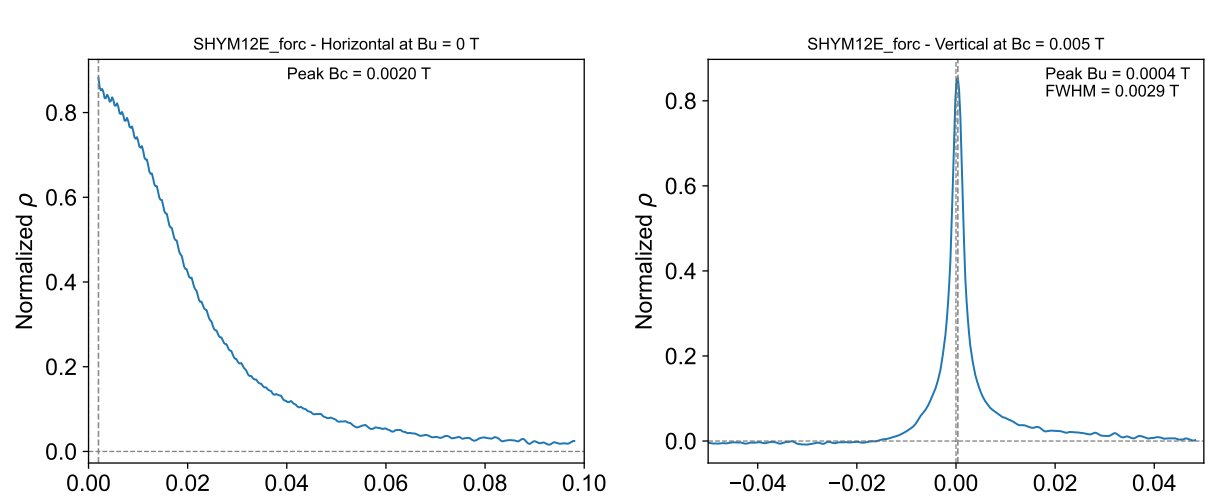

SHYM12E_forc_custom_profiles.pdf

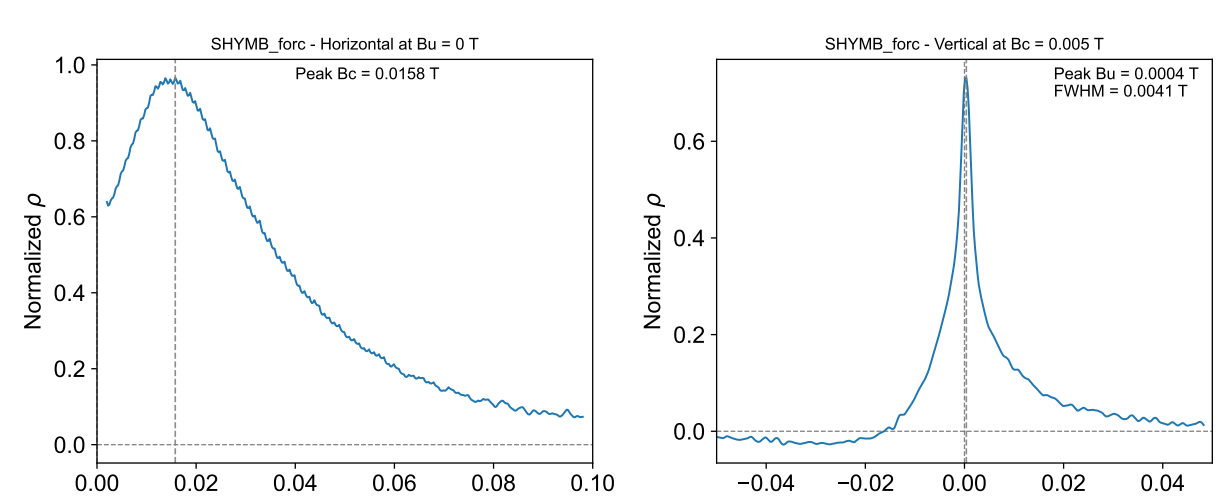

SHYMB_forc_custom_profiles.pdf

Fig. S13. Same as S12 but for samples SHY13D to SHYMB.

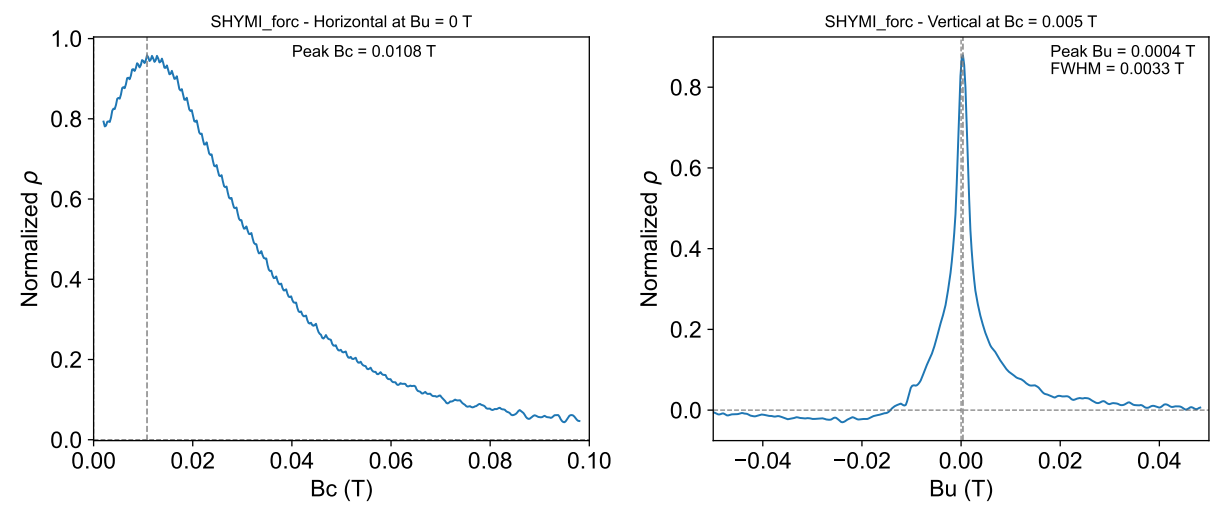

SHYMI_forc_custom_profiles.pdf

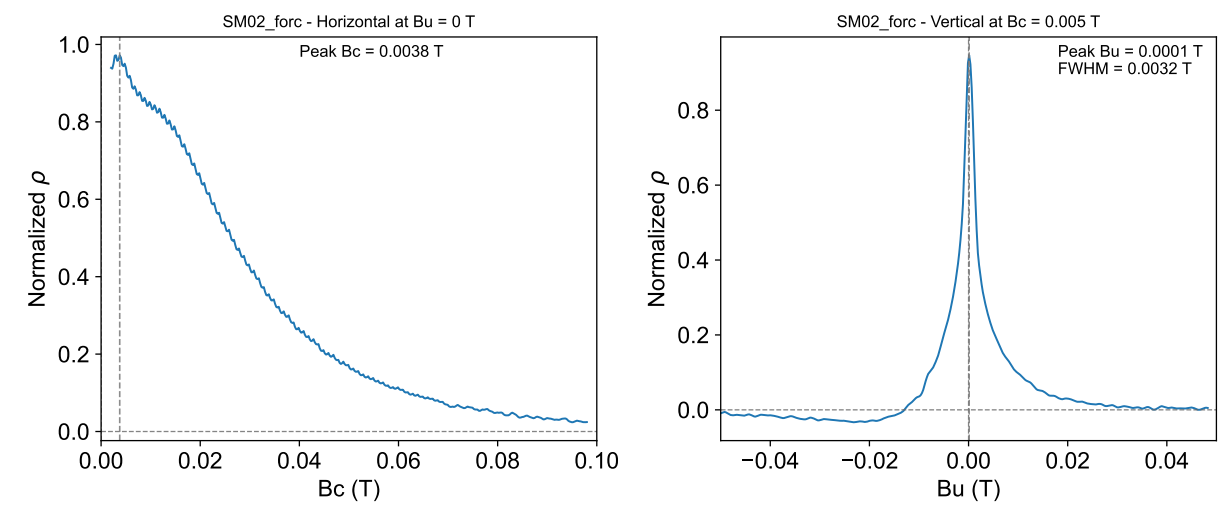

SM02_forc_custom_profiles.pdf

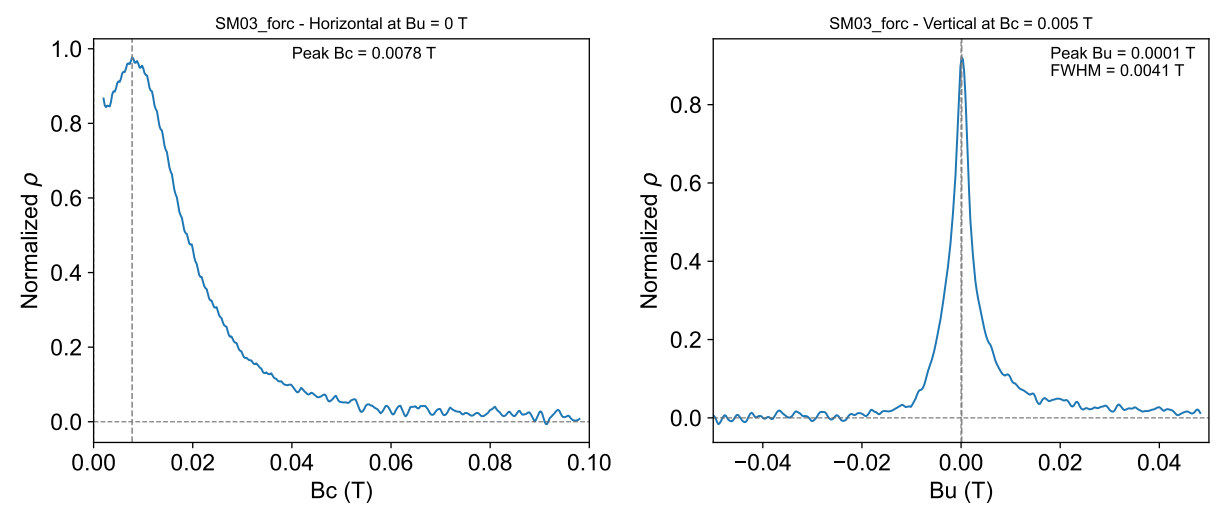

SM03_forc_custom_profiles.pdf

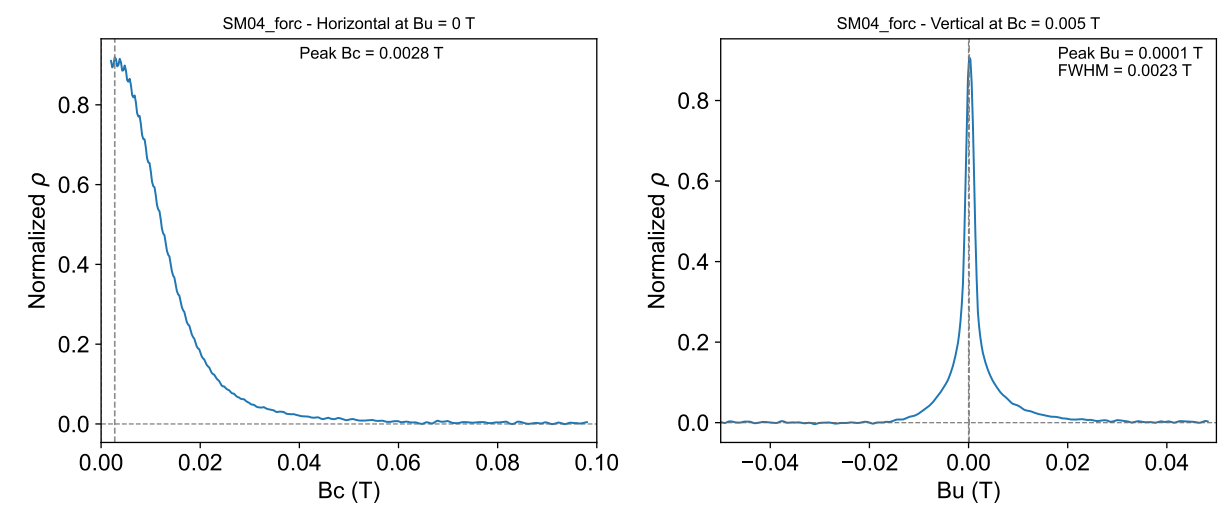

SM04_forc_custom_profiles.pdf

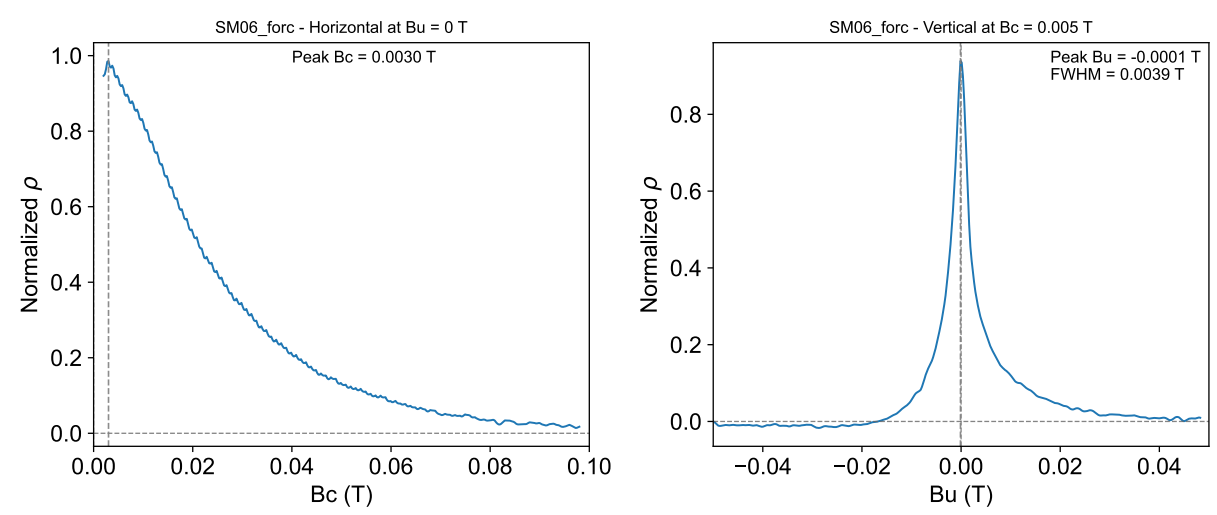

SM06_forc_custom_profiles.pdf

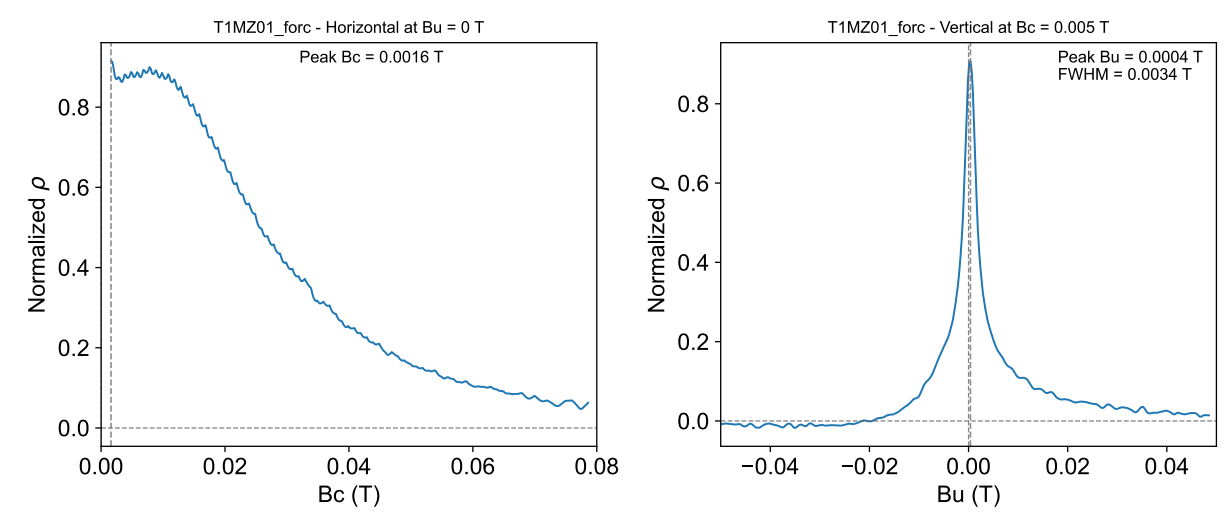

T1MZ01_forc_custom_profiles.pdf

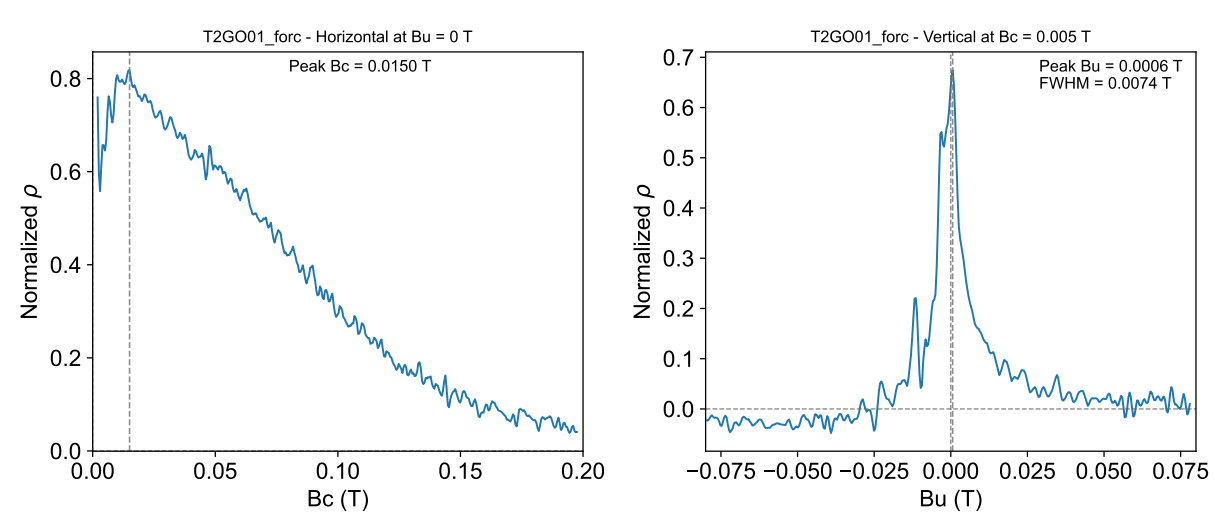

T2GO01_forc_custom_profiles.pdf

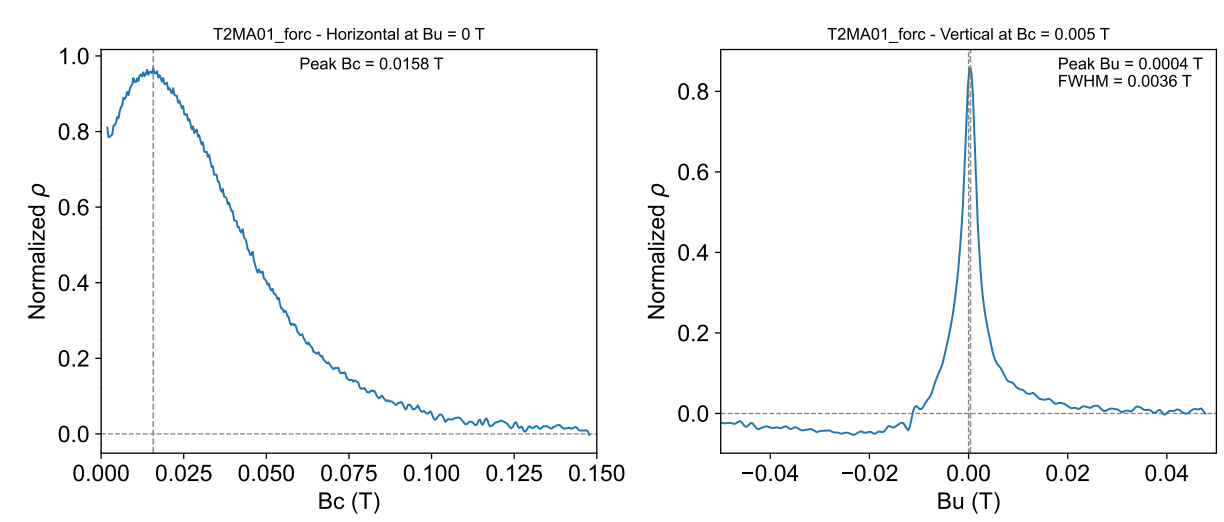

T2MA01_forc_custom_profiles.pdf

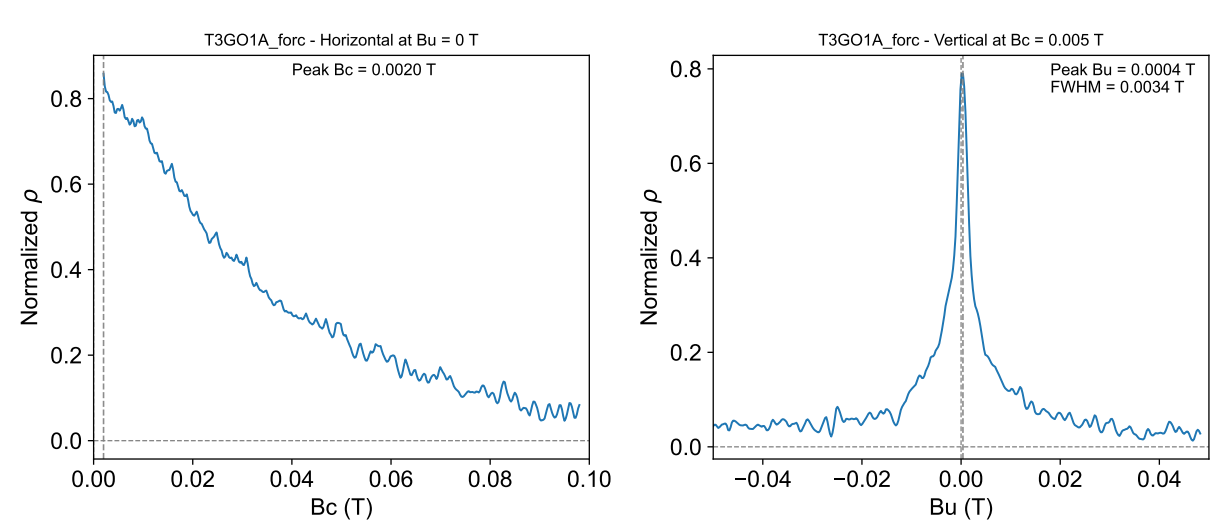

T3GO1A_forc_custom_profiles.pdf

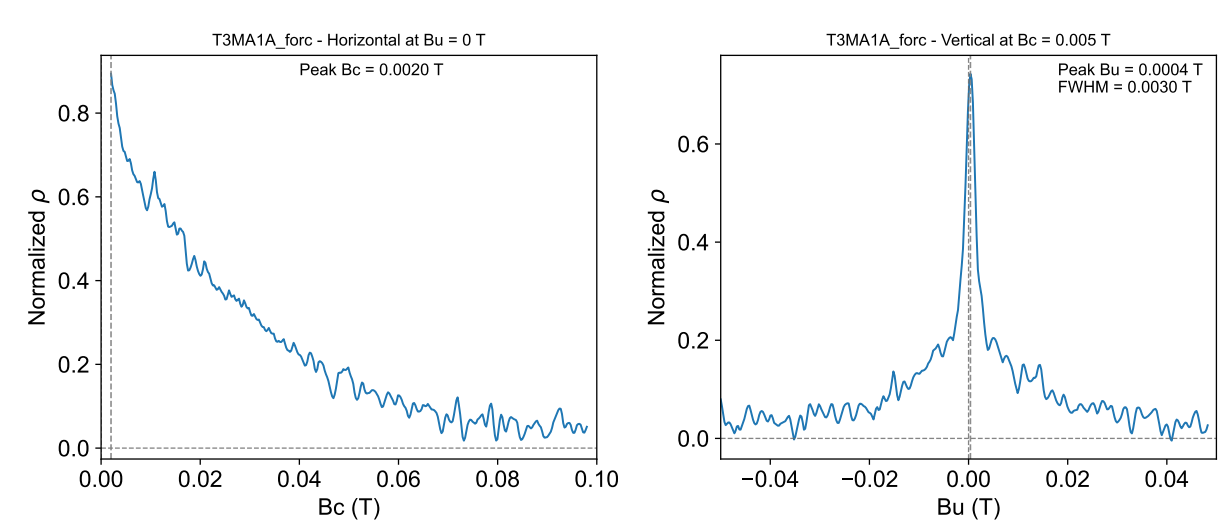

T3MA1A_forc_custom_profiles.pdf

Fig. S14. Same as S12 but for samples SHYMI to T3MA1A.

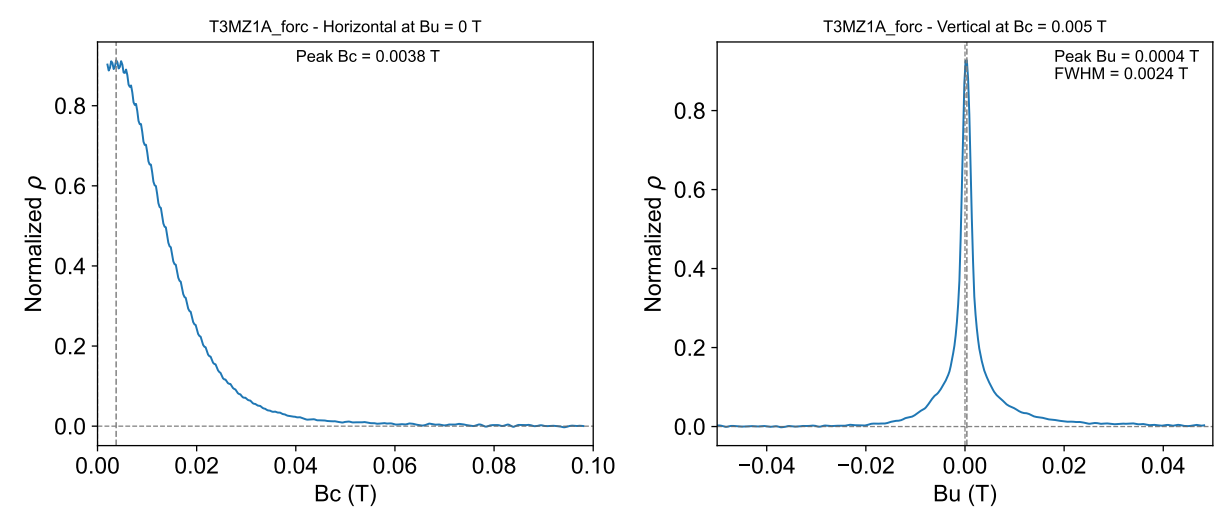

T3MZ1A_forc_custom_profiles.pdf

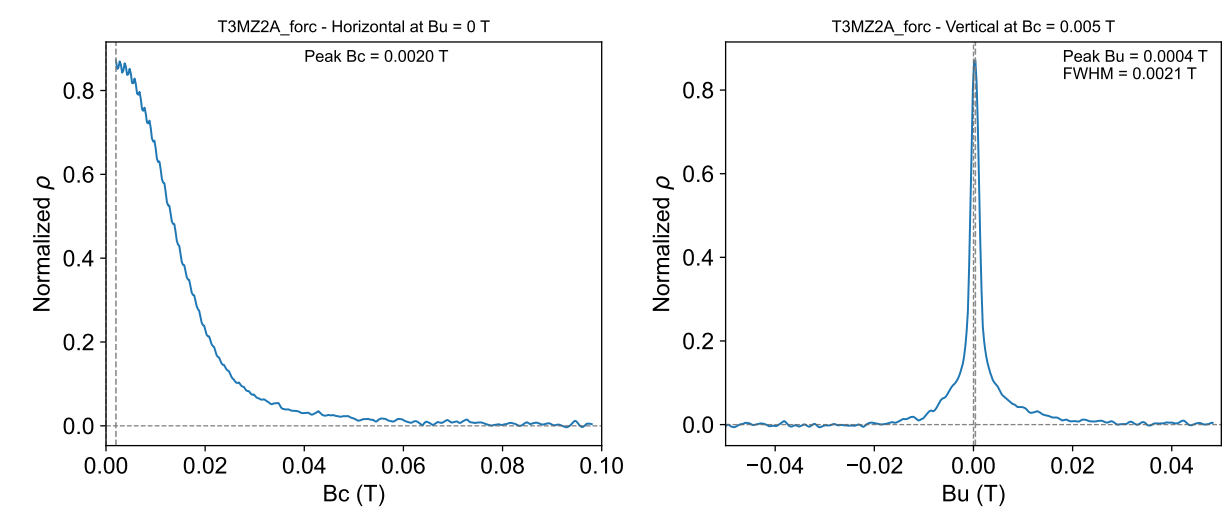

T3MZ2A_forc_custom_profiles.pdf

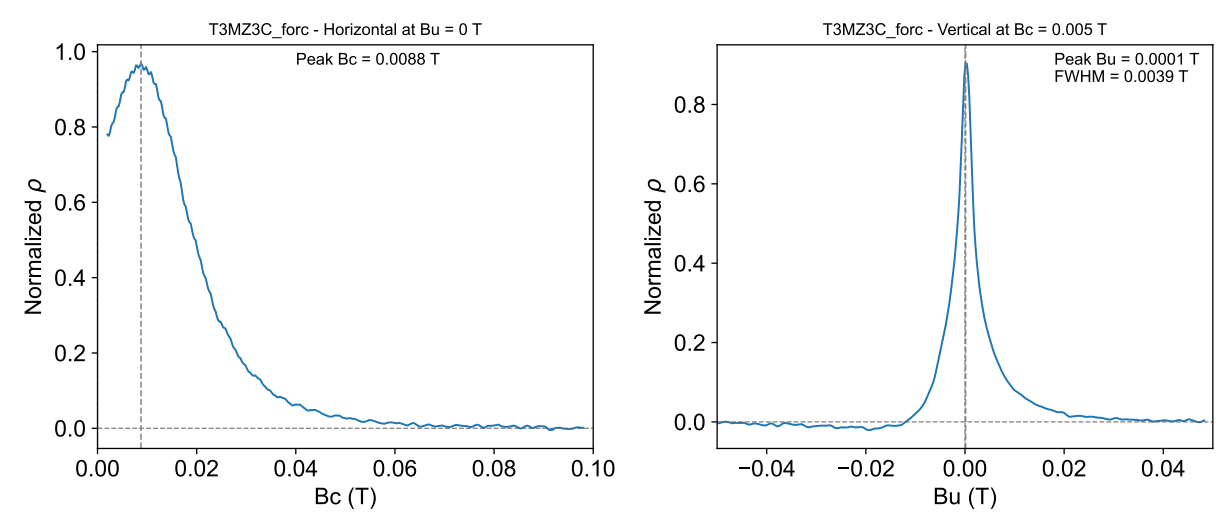

T3MZ3C_forc_custom_profiles.pdf

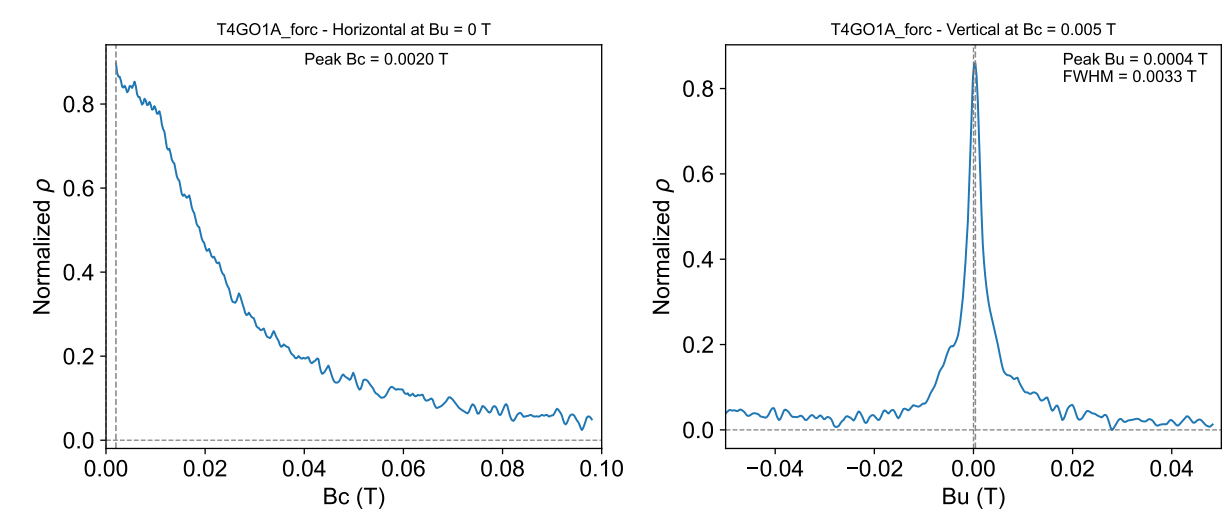

T4GO1A_forc_custom_profiles.pdf

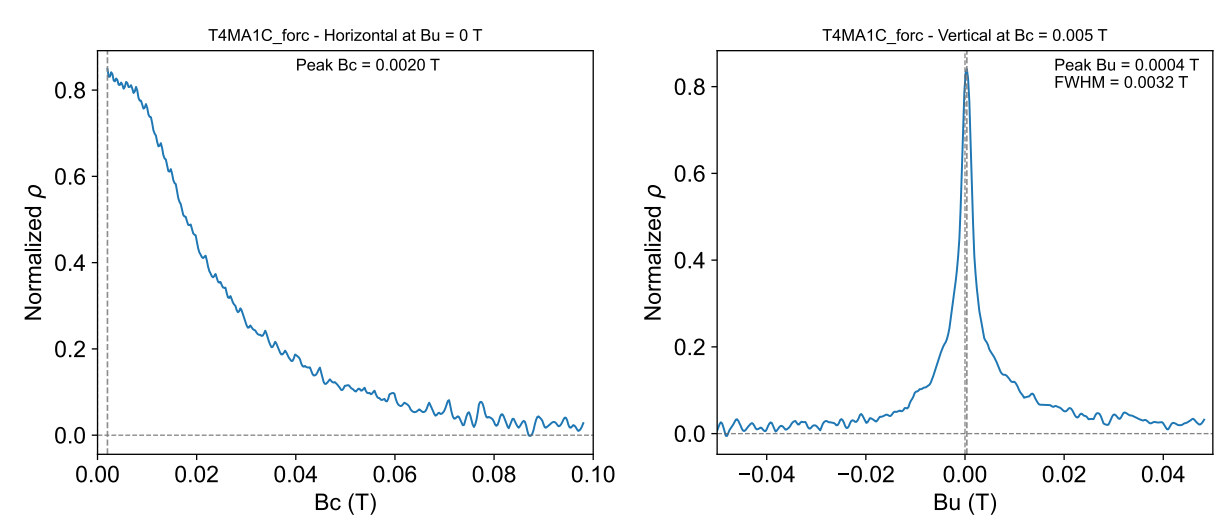

T4MA1C_forc_custom_profiles.pdf

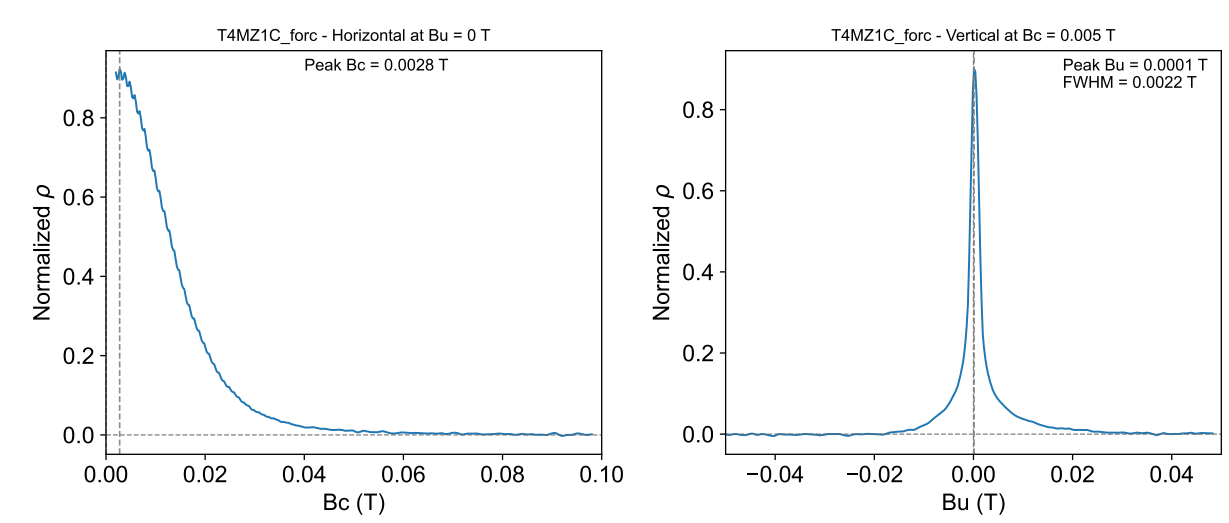

T4MZ1C_forc_custom_profiles.pdf

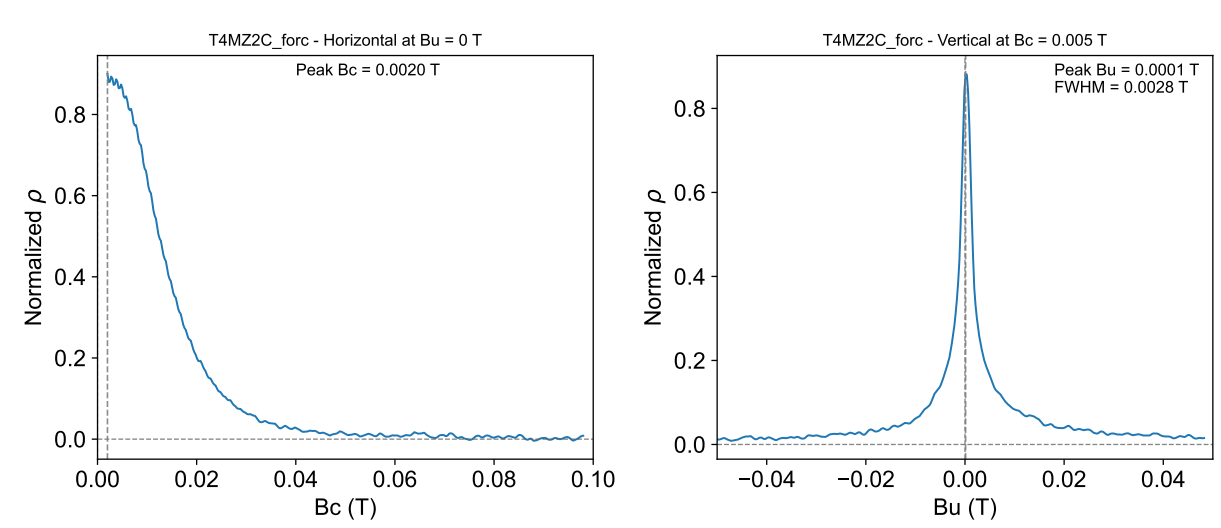

T4MZ2C_forc_custom_profiles.pdf

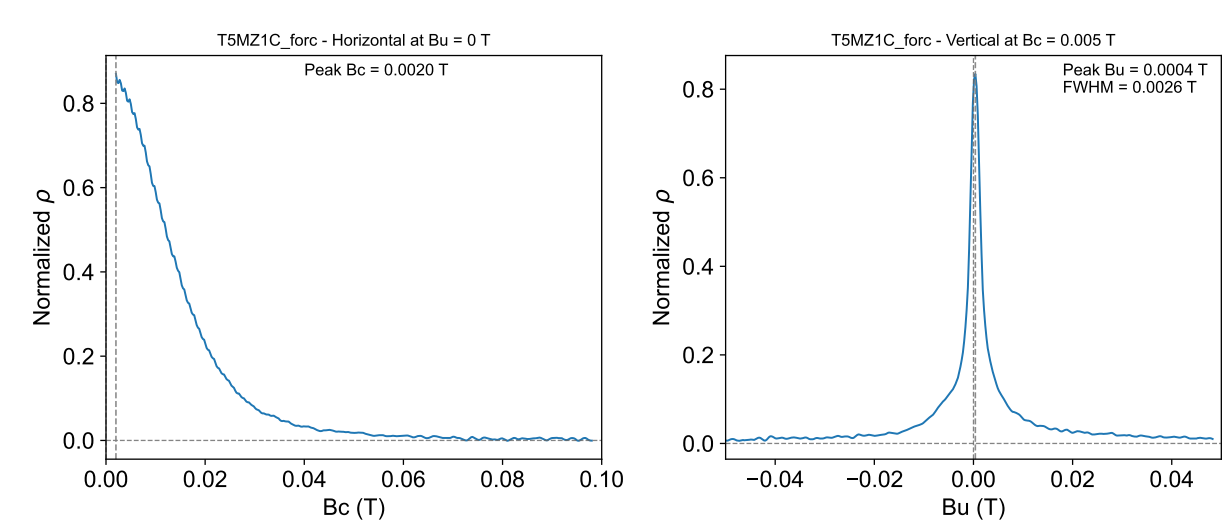

T5MZ1C_forc_custom_profiles.pdf

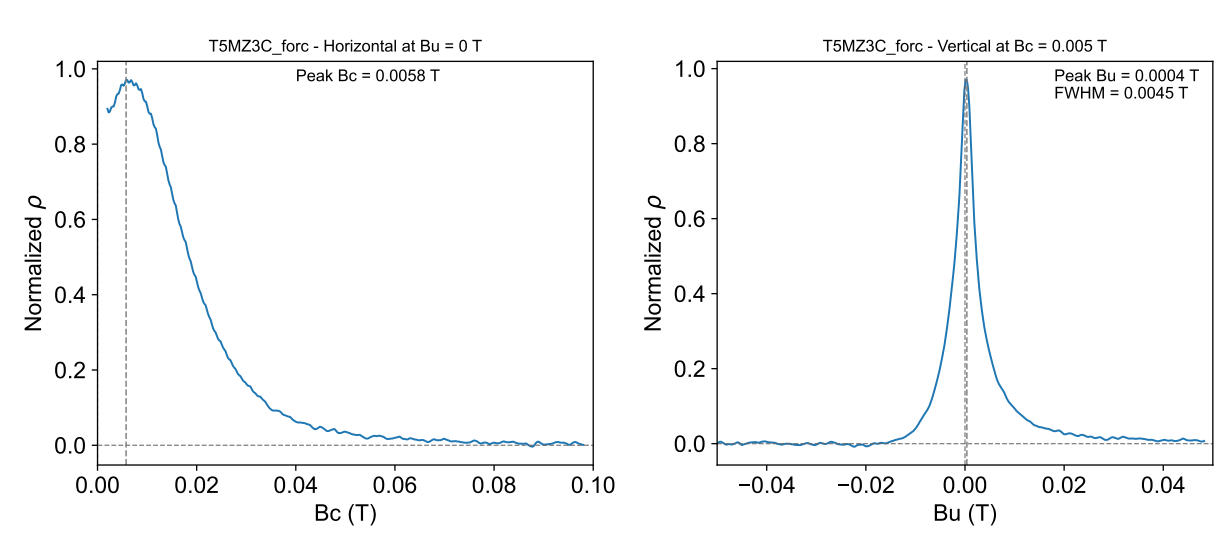

T5MZ3C_forc_custom_profiles.pdf

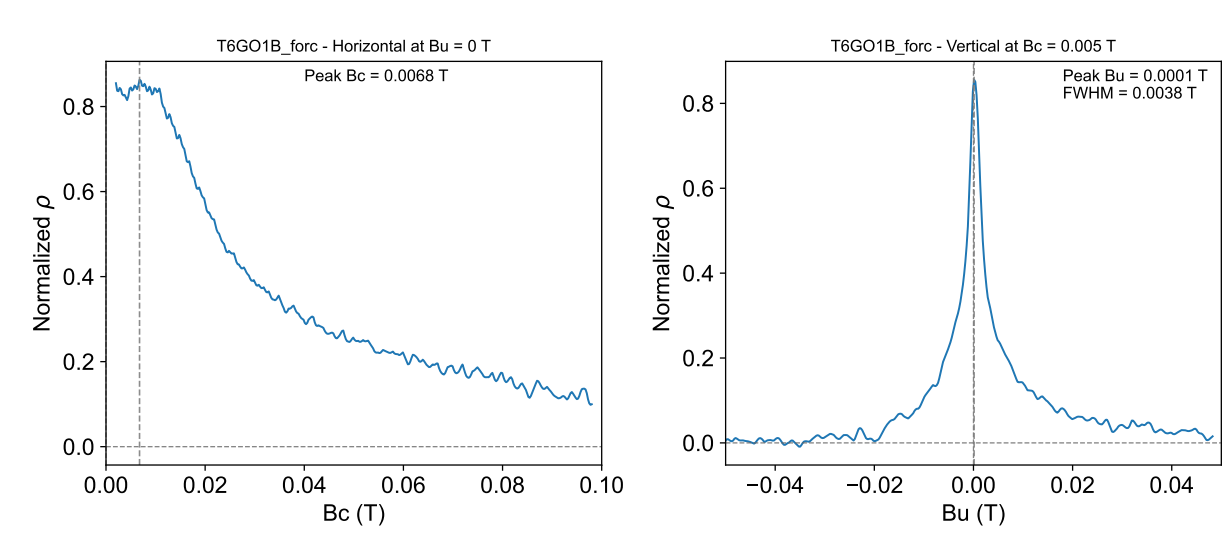

T6GO1B_forc_custom_profiles.pdf

Fig. S15. Same as S12 but for samples T3MZ1A to T6GO1B.

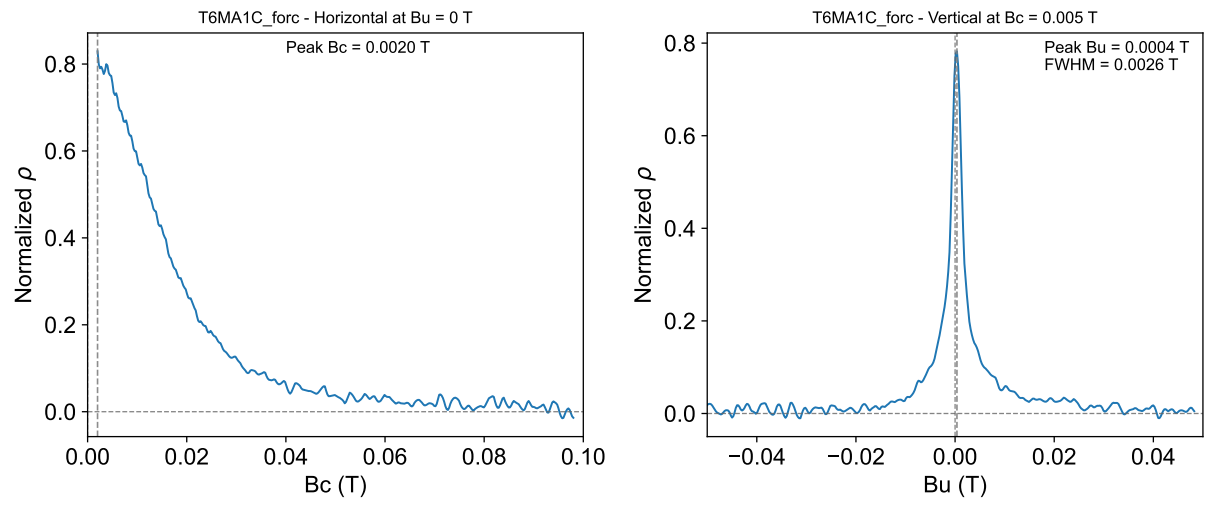

T6MA1C_forc_custom_profiles.pdf

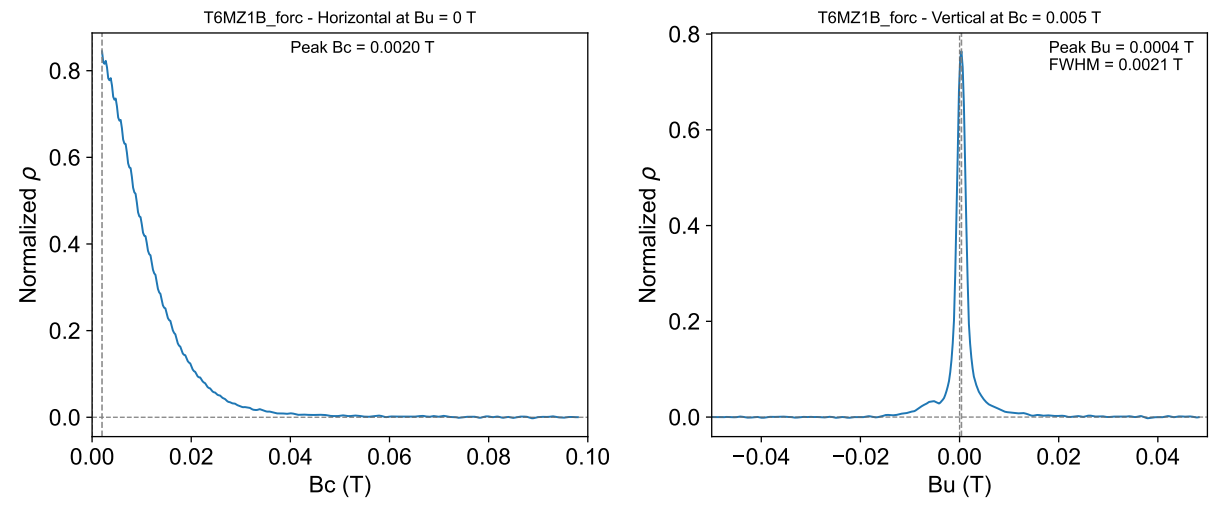

T6MZ1B_forc_custom_profiles.pdf

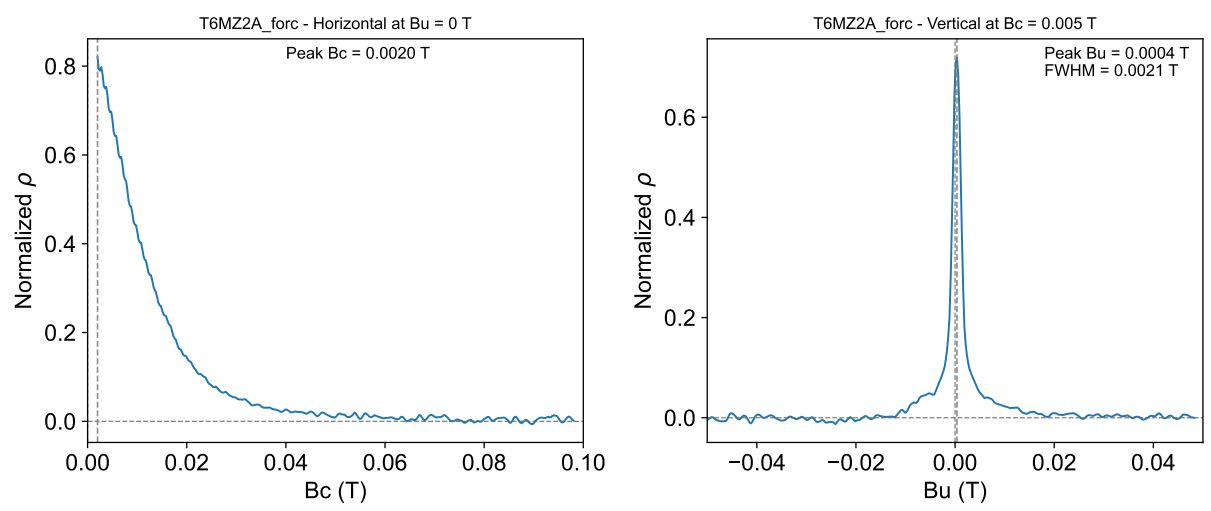

T6MZ2A_forc_custom_profiles.pdf

Fig. S16. Same as S12 but for samples T6MA1C to T6MZ2A.

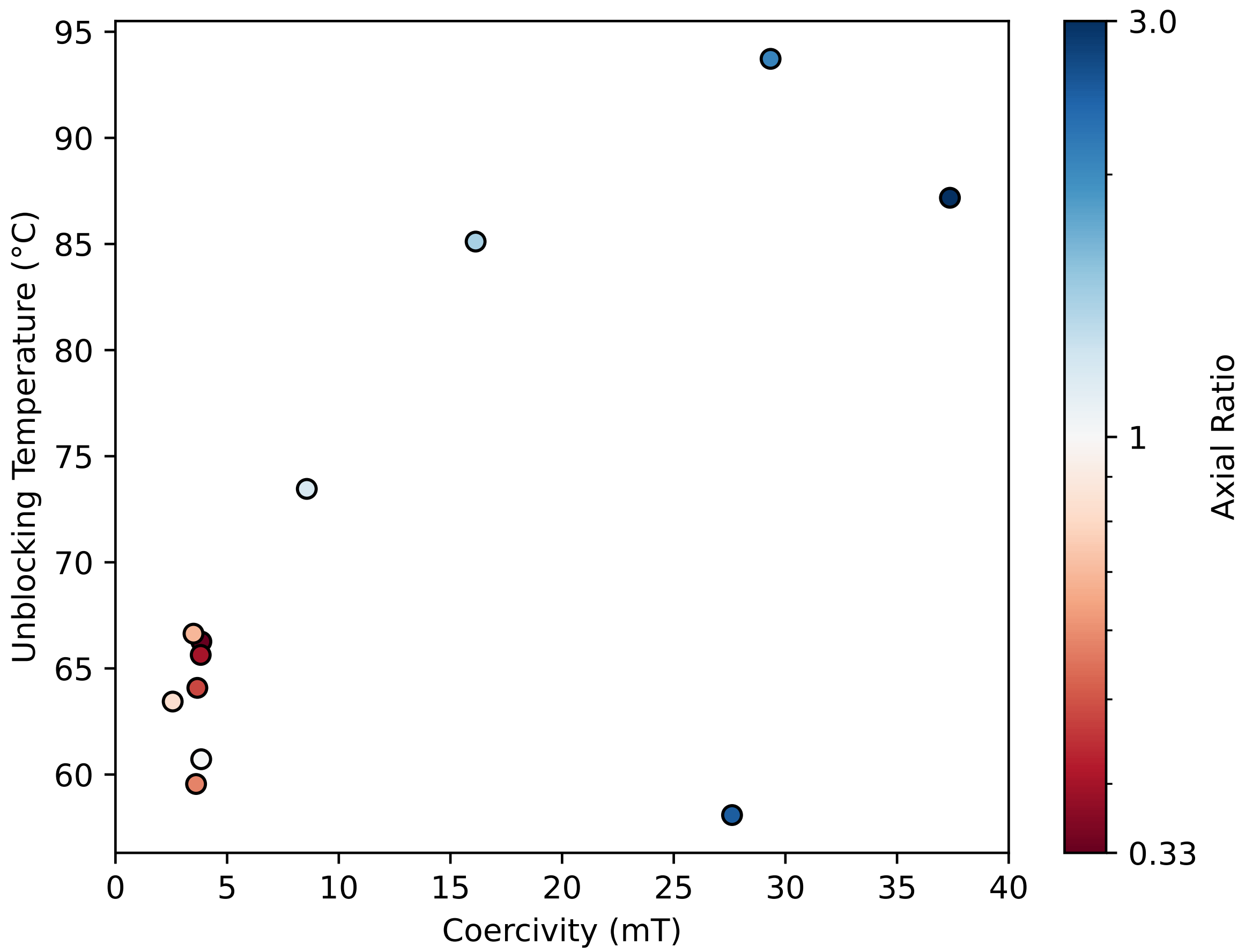

Fig. S17. Simulated coercivity vs unblocking temperatures for an assortment of particles with different shapes and sizes that acquired a VRM in the "modern" SDCC simulations (the largest such particles for each shape category). Coercivities range from 0 - 40 mT and unblocking temperatures range from 60 - 90 °C, consistent with both the demagnetization and FORC experiments. Colors denote particle shape.

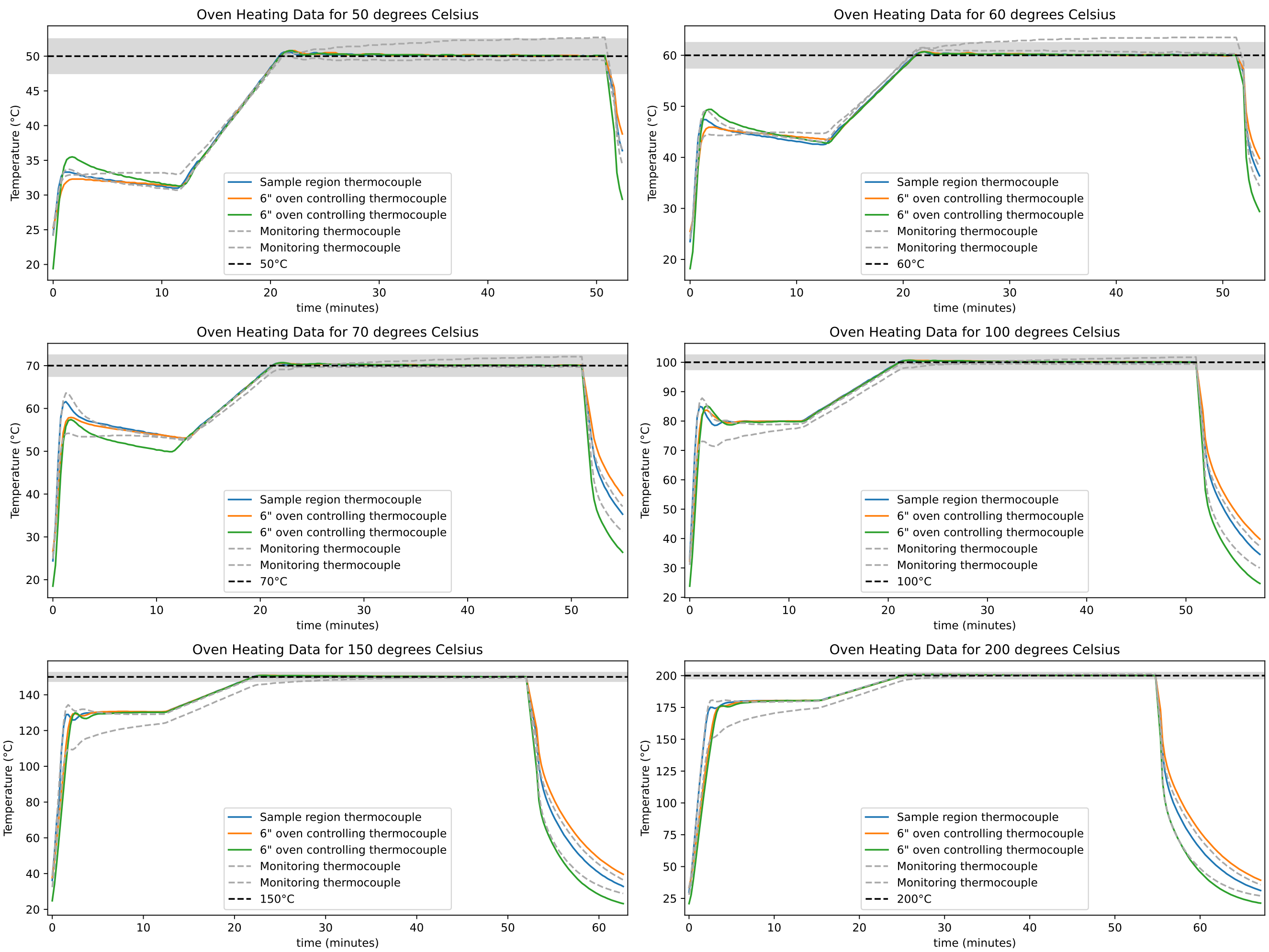

Fig. S18. Representative oven heating data. In every sub-figure, the black dashed line represents the target temperature and the gray area around it represents $\pm 2.5^{\circ}$C from the target temperature. The two gray dashed lines represent the temperatures measured by two monitoring thermocouples which are placed about 1.5" from the ends of the 18" oven. Since only the middle 12" were used for the samples, the readings of these two thermocouples do not reflect the temperature of the samples. The colored lines represent the temperatures recorded by the three controlling thermocouples: flanking 6" oven farthest away from the fan (orange) middle of the 18" oven (blue) and flanking 6" oven closest to the fan (green). The middle one is placed in the middle of the 12" portion of the 18" oven, where the samples were placed. Thus, its readings best represent the temperatures of the samples.

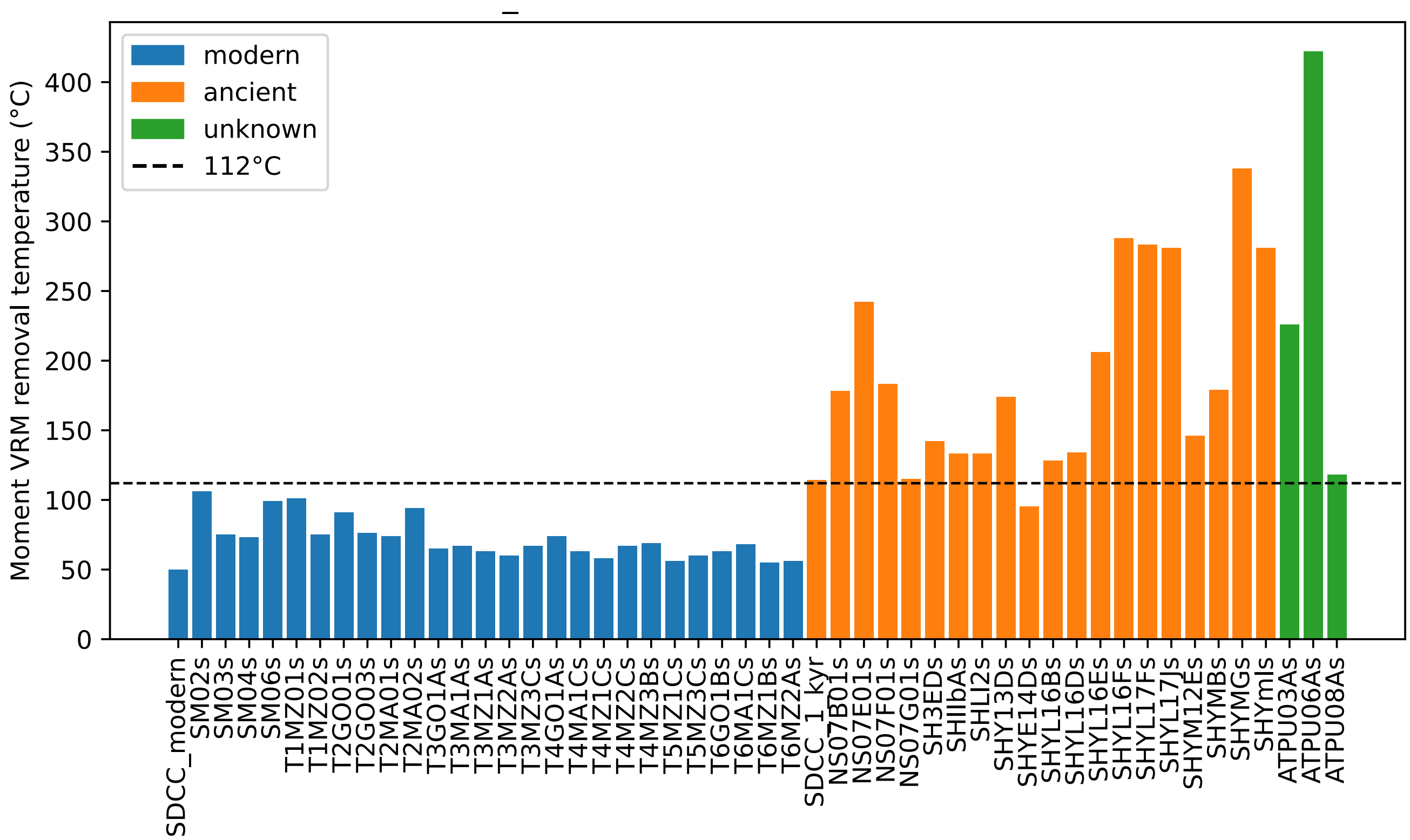

Fig. S19. VRM Removal Temperature (VRT) results of all 48 actual specimens and two simulated specimens according to the moment decay (VRT_M). The dashed black line marks the 112°C threshold.

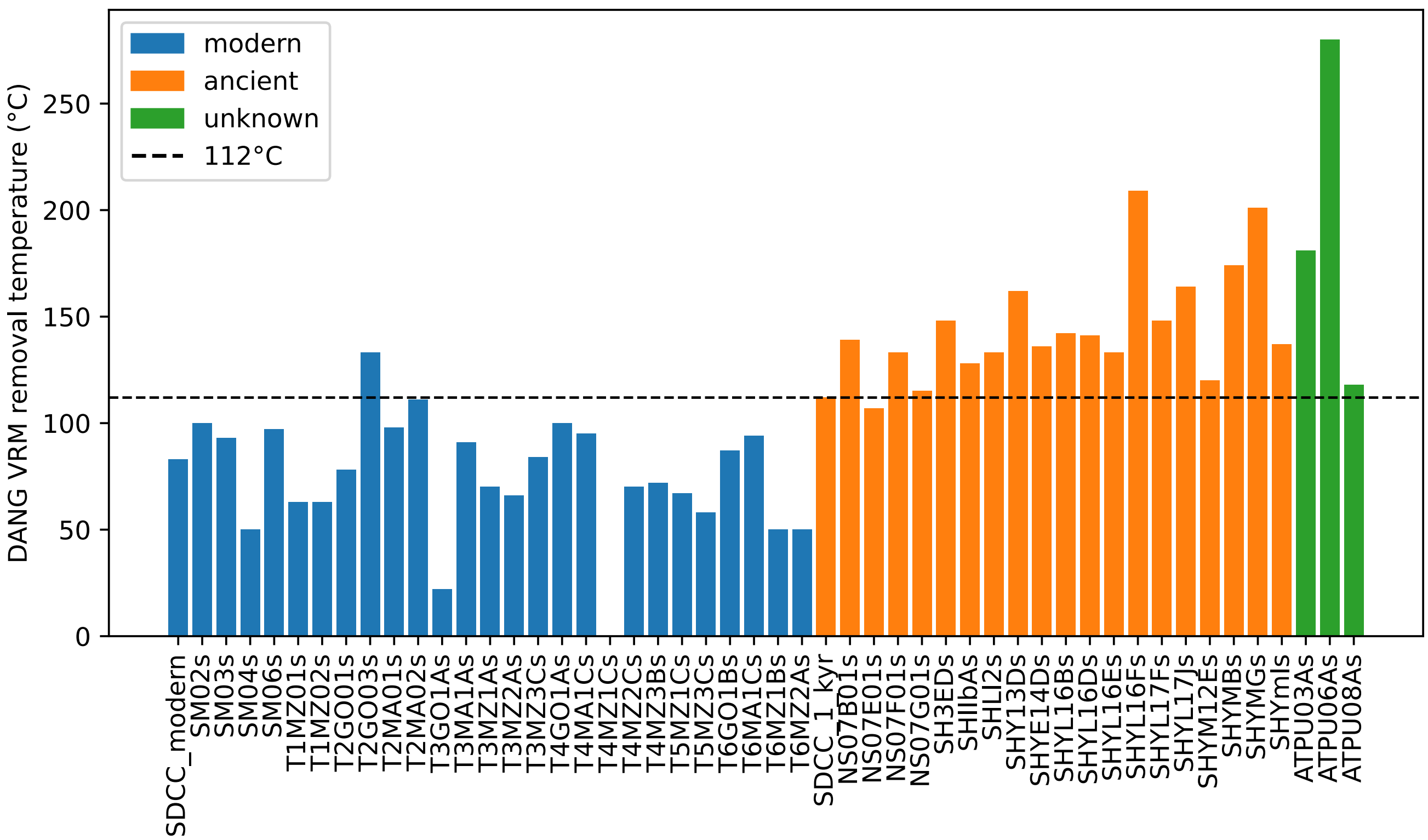

Fig. S20. VRM Removal Temperature (VRT) results of all 48 actual specimens and two simulated specimens according to the DANG (VRT_D). The dashed black line marks the 112°C threshold, which is the VRT_D of the 1000-year-old simulated specimen.

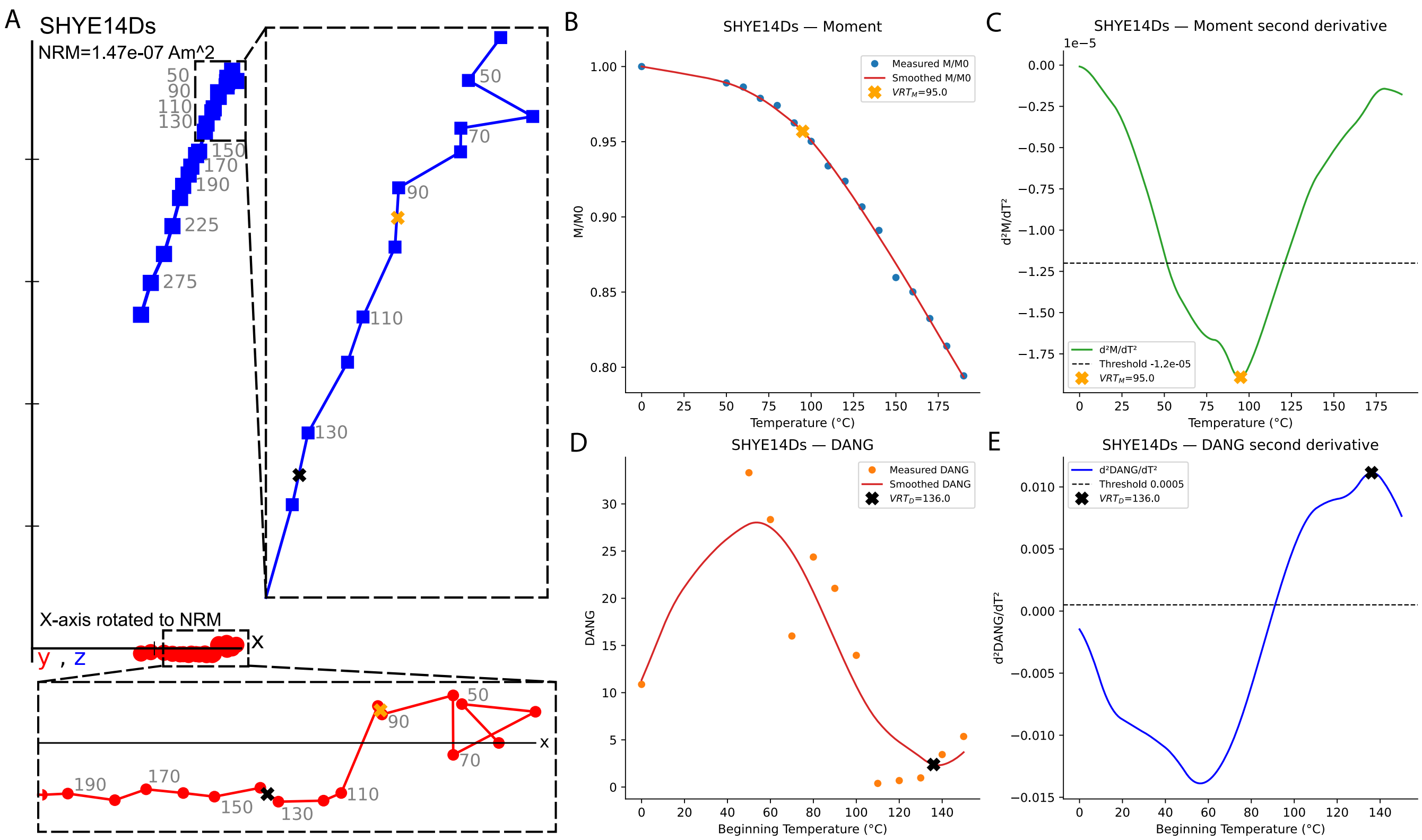

Fig. S21. Results of an ancient object which yielded a VRT of $115.5°$C but displayed a contradiction between the VRT_M ($95°$C) and the VRT_D ($136°$C). We suggest that in this case the VRM was recorded in an orientation similar to that of the TRM. (A) Zijderveld (21) diagram presenting thermal demagnetization with zoom-in views of each of the two components. Notice the point where the red line becomes roughly straight converging towards the origin at $110 - 120°$C). (B) The magnetic moment, normalized to the initial moment, remaining after every thermal demagnetization step (blue dots) and a curve representing the data after smoothing by a Savitzky–Golay (11) filter (red line). (C) The second derivative of the curve presented in (B). The VRM removal temperature according to the moment (VRT_M) is marked by the orange X here and in (A-B). (D) The DANG calculated from every starting point (orange dots- see main text for details) and a curve representing the data after smoothing by a Savitzky–Golay (11) filter (red line). (E) The second derivative of the curve presented in (D). The VRM removal temperature according to the DANG (VRT_D) is marked by the black X here and in (A) and (D).

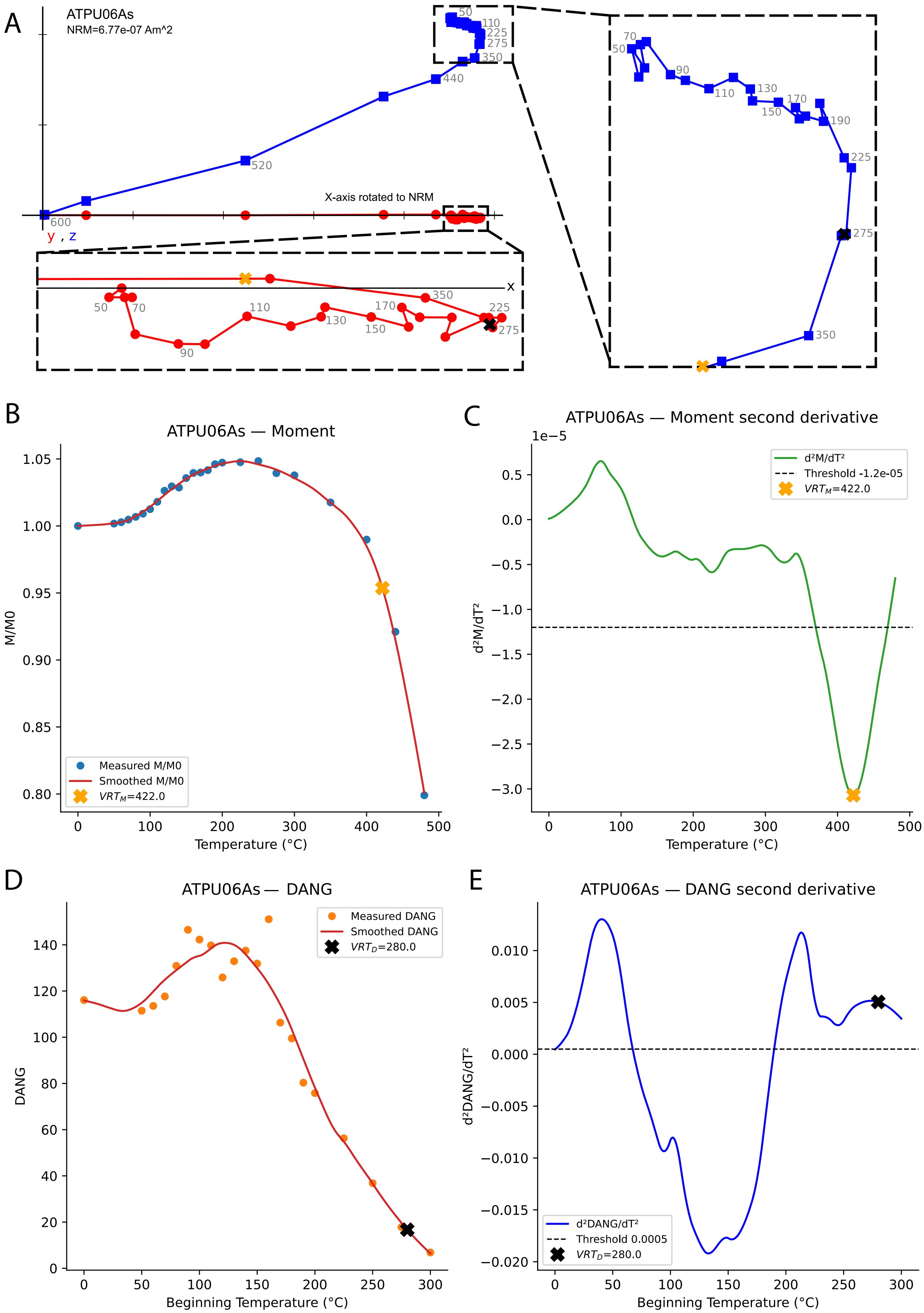

Fig. S22. Results of an ancient specimen which was most likely reheated to $350 - 400°C$ and thus contains two TRM components and at least one VRM component. Sub-figures same as in Figure S21.

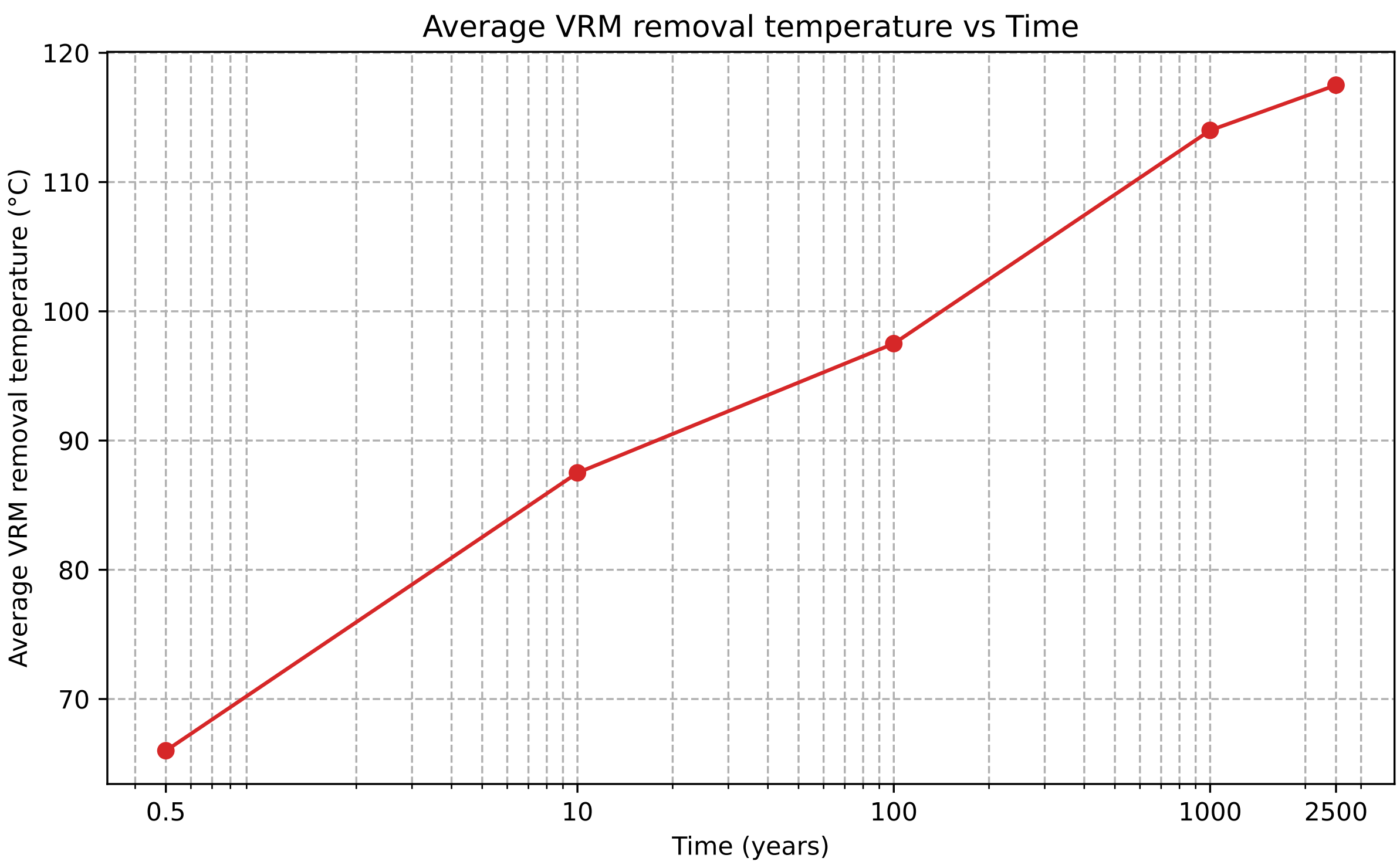

Fig. S23. SDCC simulation results presenting the VRT (average of VRT_M and VRT_D) for different VRM acquisition times. The time axis is logarithmic.

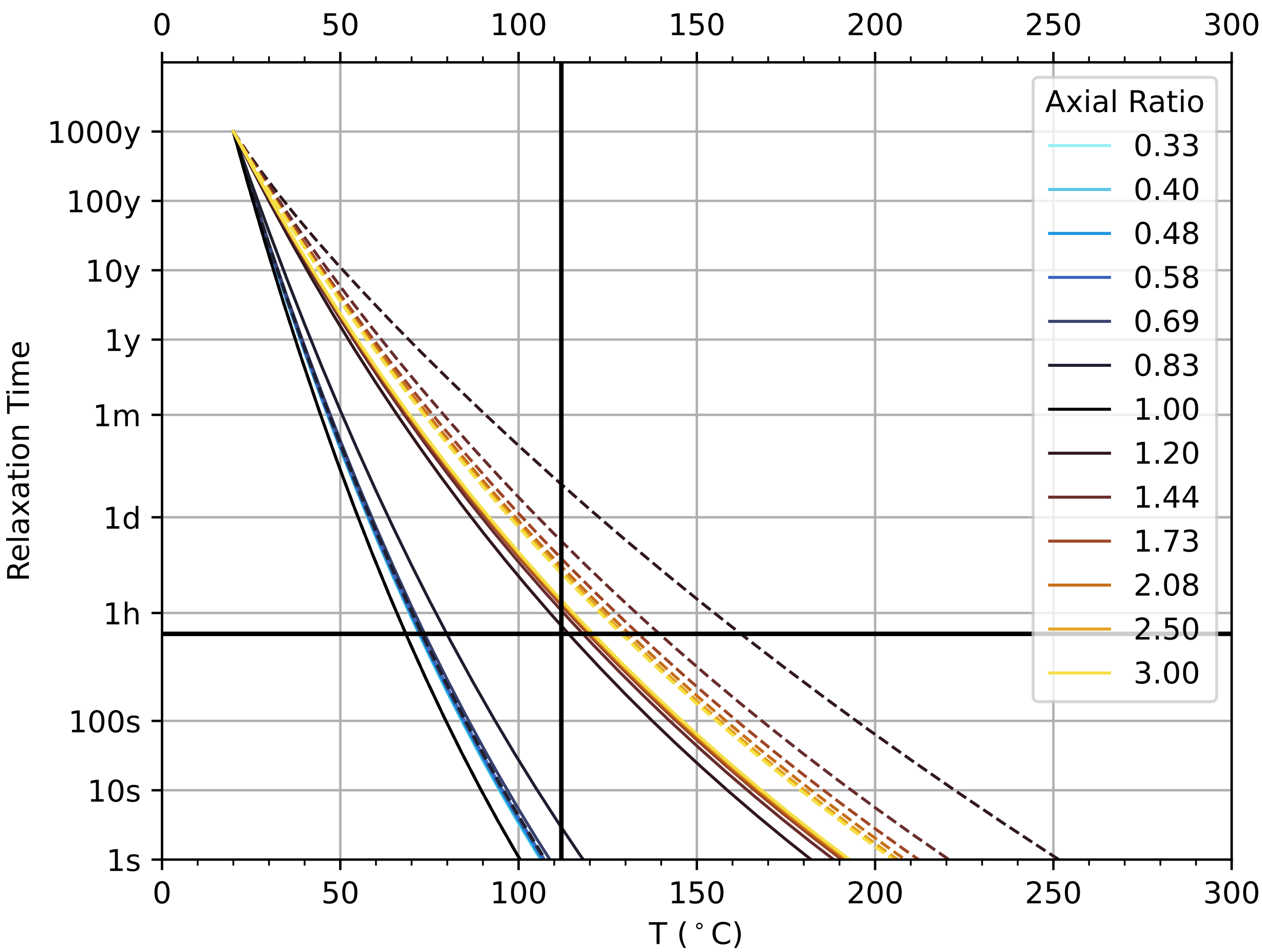

Fig. S24. The effect of particle shape on VRM removal temperature calculated with the SDCC (1, 2). Thermoviscous Stability Curves (following Pullaiah et al. 22), for particles with their easy (solid lines) or hard (dashed lines) axis aligned with the long axis. Red and yellow lines represent prolate particles, black lines represent spherical particles and blue lines represent oblate disks. The prolate trend will also dominate in particles with a small degree of triaxial shape. Black horizontal line represents the laboratory heating time, and black vertical line represents 112°C.

Table S1. Results: Modern Specimens

Specimen Name	Kiln Used for Firing	Clay Type	Moment VRM Removal Temp. (VRT_M)	DANG VRM Removal Temp. (VRT_D)
SM02s	Unknown	Unknown	$106°C$	$100°C$
SM03s	Unknown	Unknown	$75°C$	$93°C$
SM04s	Unknown	Unknown	$73°C$	$50°C$
SM06s	Unknown	Unknown	$99°C$	$97°C$
T1MZ01s	Traditional kiln- T1	Motza (MZ1)	$101°C$	$63°C$
T1MZ02s	Traditional kiln- T1	Motza (MZ1)	$75°C$	$63°C$
T2GO01s	Traditional kiln- T2	Golan (GO)	$91°C$	$78°C$
T2GO03s	Traditional kiln- T2	Golan (GO)	$76°C$	$133°C$
T2MA01s	Traditional kiln- T2	Mamshit (MA)	$74°C$	$98°C$
T2MA02s	Traditional kiln- T2	Mamshit (MA)	$94°C$	$111°C$
T3GO1As	Electric kiln- T3	Golan (GO)	$65°C$	$22°C$
T3MA1As	Electric kiln- T3	Mamshit (MA)	$67°C$	$91°C$
T3MZ1As	Electric kiln- T3	Motza (MZ1)	$63°C$	$70°C$
T3MZ2As	Electric kiln- T3	Motza (MZ2)	$60°C$	$66°C$
T3MZ3Cs	Electric kiln- T3	Motza (MZ3)	$67°C$	$84°C$
T4GO1As	Electric kiln- T4	Golan (GO)	$74°C$	$100°C$
T4MA1Cs	Electric kiln- T4	Mamshit (MA)	$63°C$	$95°C$
T4MZ1Cs	Electric kiln- T4	Motza (MZ1)	$58°C$	$0°C$
T4MZ2Cs	Electric kiln- T4	Motza (MZ2)	$67°C$	$70°C$
T4MZ3Bs	Electric kiln- T4	Motza (MZ3)	$69°C$	$72°C$
T5MZ1Cs	Electric kiln- T5	Motza (MZ1)	$56°C$	$67°C$
T5MZ3Cs	Electric kiln- T5	Motza (MZ3)	$60°C$	$58°C$
T6GO1Bs	Electric kiln- T6	Golan (GO)	$63°C$	$87°C$
T6MA1Cs	Electric kiln- T6	Mamshit (MA)	$68°C$	$94°C$
T6MZ1Bs	Electric kiln- T6	Motza (MZ1)	$55°C$	$50°C$
T6MZ2As	Electric kiln- T6	Motza (MZ2)	$56°C$	$50°C$

Table S2. Results: Ancient Specimens

Specimen Name	Moment VRM Removal Temp. (VRT_M)	DANG VRM Removal Temp. (VRT_D)
NS07B01s	$178°C$	$139°C$
NS07E01s	$242°C$	$107°C$
NS07F01s	$183°C$	$133°C$
NS07G01s	$115°C$	$115°C$
SH3EDs	$142°C$	$148°C$
SHIIbAs	$133°C$	$128°C$
SHLI2s	$133°C$	$133°C$
SHY13Ds	$174°C$	$162°C$
SHYE14Ds	$95°C$	$136°C$
SHYL16Bs	$128°C$	$142°C$
SHYL16Ds	$134°C$	$141°C$
SHYL16Es	$206°C$	$133°C$
SHYL16Fs	$288°C$	$209°C$
SHYL17Fs	$283°C$	$148°C$
SHYL17Js	$281°C$	$164°C$
SHYM12Es	$146°C$	$120°C$
SHYMBs	$179°C$	$174°C$
SHYMGs	$338°C$	$201°C$
SHYMIs	$281°C$	$137°C$

Table S3. Results: Unknown Specimens

Specimen Name	Moment VRM Removal Temp. (VRT_M)	DANG VRM Removal Temp. (VRT_D)
ATPU03As	$226°C$	$181°C$
ATPU06As	$422°C$	$280°C$
ATPU08As	$118°C$	$118°C$

Table S4. VRT (mean of VRT_M and VRT_D) for the five specimens whose classification is sensitive to the cutoff choice, at each threshold tested. The bold text marks a value that crosses the 112°C threshold relative to the specimen's known type (i.e., a misclassification). All other comparable specimens retain the same classification at every threshold tested.

Specimen Name	Known Type	VRT@80%	VRT@70%	VRT@60%	VRT@50%
SHYE14Ds	ancient	$115.5°C$	$\mathbf{102.0°C}$	$\mathbf{102.0°C}$	$\mathbf{102.0°C}$
SM03s	modern	$84.0°C$	$\mathbf{169.0°C}$	$\mathbf{166.5°C}$	$\mathbf{166.5°C}$
T6GO1Bs	modern	$75.0°C$	$75.0°C$	$\mathbf{200.0°C}$	$\mathbf{189.0°C}$
T6MA1Cs	modern	$81.0°C$	$73.0°C$	$\mathbf{134.0°C}$	$\mathbf{158.0°C}$
T4GO1As	modern	$87.0°C$	$87.0°C$	$87.0°C$	$\mathbf{251.0°C}$

References

1. B Cych, G Paterson, L Nagy, W Williams, First principles understanding of single domain magnetizations – Part i: the single domain comprehensive calculator (SDCC) open source library. *Geophysical Journal International* **241**, 2034–2048 (2025).
2. B Cych, GA Paterson, L Nagy, W Williams, L Tauxe, First principles understanding of single domain magnetizations - Part II: Non-ideal magnetic behavior in ideal single domain titanomagnetite. *Geophys. J. Int.* p. ggag090 (2026).
3. D Atkinson, JA King, Fine particle magnetic mineralogy of archaeological ceramics. *J. Physics: Conf. Ser.* **17**, 145 (2005).
4. M Kostadinova-Avramova, M Kovacheva, The magnetic properties of baked clays and their implications for past geomagnetic field intensity determinations. *Geophys. J. Int.* **195**, 1534–1550 (2013).
5. G Eramo, Ceramic technology: how to recognize clay processing. *Archaeol. Anthropol. Sci.* **12**, 1–24 (2020).
6. L Bar-Sovik, et al., Testing the accuracy of paleointensity estimates using experimental pottery assemblages. *J. Geophys. Res. Solid Earth* **131**, e2025JB033568 (2026) e2025JB033568 2025JB033568.
7. M Brown, FORCme: Application of machine code and adaptive smoothing within python for the rapid processing of First Order Reversal Curves, doi:10.5281/zenodo.19636066 (2026).
8. L Tauxe, H Staudigel, Strength of the geomagnetic field in the Cretaceous Normal Superchron: New data from submarine basaltic glass of the Troodos Ophiolite. *Geochem. Geophys. Geosystems* **5** (2004).
9. H Tanaka, T Kobayashi, Paleomagnetism of the late quaternary ontake volcano, japan: directions, intensities, and excursions. *Earth, Planets Space* **55**, 189–202 (2003).
10. L Tauxe, et al., PmagPy: software package for paleomagnetic data analysis and a bridge to the Magnetics Information Consortium (MagIC) Database. *Geochem. Geophys. Geosystems* **17**, 2450–2463 (2016).
11. A Savitzky, MJE Golay, Smoothing and differentiation of data by simplified least squares procedures. *Anal. Chem.* **36**, 1627–1639 (1964).
12. P Virtanen, et al., SciPy 1.0: fundamental algorithms for scientific computing in Python. *Nat. Methods* **17**, 261–272 (2020).
13. E Hassul, et al., Geomagnetic field intensity during the first millennium BCE From Royal Judean Storage Jars: Constraining the duration of the Levantine Iron Age Anomaly. *Geochem. Geophys. Geosystems* **25**, e2023GC011263 (2024) e2023GC011263 2023GC011263.
14. Y Vaknin, et al., Pre-excavation identification and dating of Iron Age Destruction Events. *Archaeol. Prospect.* **32**, 1021–1030 (2025).
15. AP Roberts, CR Pike, KL Verosub, First-order reversal curve diagrams: A new tool for characterizing the magnetic properties of natural samples. *J. Geophys. Res. Solid Earth* **105**, 28461–28475 (2000).
16. AP Roberts, D Heslop, X Zhao, CR Pike, Understanding fine magnetic particle systems through use of first-order reversal curve diagrams. *Rev. Geophys.* **52**, 557–602 (2014).
17. R Egli, AP Chen, M Winklhofer, KP Kodama, CS Horng, Detection of noninteracting single domain particles using first-order reversal curve diagrams. *Geochem. Geophys. Geosystems* **11** (2010).
18. AP Roberts, et al., Resolving the origin of pseudo-single domain magnetic behavior. *J. Geophys. Res. Solid Earth* **122**, 9534–9558 (2017).
19. AJ Newell, A high-precision model of first-order reversal curve (forc) functions for single-domain ferromagnets with uniaxial anisotropy. *Geochem. Geophys. Geosystems* **6** (2005).
20. H Goren, Y Goren, Moses wilhelm shamoabiticapira, hermann weser and the moab expedition: Provenance study of the moabitica. *Palest. Explor. Q.* **157**, 311–331 (2025).
21. JDA Zijderveld, *A.C. demagnetization of rocks: Analysis of results*, Methods in Paleomagnetism. (Chapman and Hall), (1967).
22. G Pullaiah, E Irving, K Buchan, D Dunlop, Magnetization changes caused by burial and uplift. *Earth Planet. Sci. Lett.* **28**, 133–143 (1975).
